# Equivalent T Cell Epitope Promiscuity in Ecologically Diverse Human Pathogens

**DOI:** 10.1371/journal.pone.0073124

**Published:** 2013-08-09

**Authors:** Kirsten E. Wiens, Harish Swaminathan, Richard Copin, Desmond S. Lun, Joel D. Ernst

**Affiliations:** 1 Department of Pathology, New York University School of Medicine, New York, New York, United States of America; 2 Department of Medicine, Division of Infectious Disease, New York University School of Medicine, New York, New York, United States of America; 3 Department of Microbiology, New York University School of Medicine, New York, New York, United States of America; 4 Department of Computer Science and Center for Computational and Integrative Biology, Rutgers University, Camden, New Jersey, United States of America; University of Cape Town, South Africa

## Abstract

**Background:**

The HLA (human leukocyte antigen) molecules that present pathogen-derived epitopes to T cells are highly diverse. Correspondingly, many pathogens such as HIV evolve epitope variants in order to evade immune recognition. In contrast, another persistent human pathogen, *Mycobacterium tuberculosis*, has highly conserved epitope sequences. This raises the question whether there is also a difference in the ability of these pathogens’ epitopes to bind diverse HLA alleles, referred to as an epitope’s binding promiscuity. To address this question, we compared the in silico HLA binding promiscuity of T cell epitopes from pathogens with distinct infection strategies and outcomes of human exposure.

**Methods:**

We used computer algorithms to predict the binding affinity of experimentally-verified microbial epitope peptides to diverse HLA-DR, HLA-A and HLA-B alleles. We then analyzed binding promiscuity of epitopes derived from HIV and *M. tuberculosis*. We also analyzed promiscuity of epitopes from *Streptococcus pyogenes*, which is known to exhibit epitope diversity, and epitopes of *Bacillus anthracis* and *Clostridium tetani* toxins, as these bacteria do not depend on human hosts for their survival or replication, and their toxin antigens are highly immunogenic human vaccines.

**Results:**

We found that *B. anthracis* and *C. tetani* epitopes were the most promiscuous of the group that we analyzed. However, there was no consistent difference or trend in promiscuity in epitopes contained in HIV, *M. tuberculosis*, and *S. pyogenes*.

**Conclusions:**

Our results show that human pathogens with distinct immune evasion strategies and epitope diversities exhibit equivalent levels of T cell epitope promiscuity. These results indicate that differences in epitope promiscuity do not account for the observed differences in epitope variation and conservation.

## Introduction

MHC (major histocompatibility complex) molecules recognize and bind epitopes derived from foreign and self proteins in order to initiate and maintain adaptive immune responses. The HLA (human leukocyte antigen) alleles that encode MHC molecules are extremely diverse: over 2000 HLA class I, and over 600 HLA class II alleles have been identified [1]. In a model of host–pathogen coevolution, this diversity is maintained through selection for individuals with heterozygous HLA alleles and/or for individuals with rare HLA alleles [2]. Individuals heterozygous for HLA alleles produce a greater diversity of HLA molecules, and therefore may develop an immune response against a greater breadth of pathogen epitopes [3,4]. In addition, pathogens are more likely to develop adaptations in response to the most common HLA alleles in a population, and therefore individuals with rare alleles may have a selective advantage [5]. A common adaptation used by pathogens to avoid immune recognition is antigenic variation, in which epitope variants that decrease the likelihood of HLA binding are selected [6]. Human immunodeficiency virus (HIV) is an extreme example; new variants appear at each successive generation, which eventually leads to immune evasion and disease progression, and has frustrated vaccine development efforts [7]. In this way, hosts and their pathogens can be locked in a never-ending “arms race” to diversify their HLA allele or epitope sequences, respectively. This is known as antagonistic coevolution, or the Red Queen model [5,6].

We recently reported that *Mycobacterium tuberculosis*, a highly successful persistent human pathogen, has highly conserved epitope sequences [8]. This apparently conflicts with the general model of a host–pathogen coevolution, and emphasizes that *M. tuberculosis* employs unique approaches to achieve success as a pathogen.

It has also been shown in a variety of pathogens that, even though the adaptive immune response is highly specific, individual HLA class II-restricted peptides [9-13] and HLA class I-restricted peptides [14-18] may bind many different HLA alleles; a trait termed epitope promiscuity. Given the extensive human HLA allele diversity and varied pathogen epitope diversity, we were interested in determining whether the extent of epitope promiscuity varies in pathogens with distinct ecological niches and interactions with human hosts.

We compared epitope promiscuity in *M. tuberculosis* and HIV, since these human-specific pathogens vary in their epitope diversity [7,19] yet both persist in the face of antigen-specific T cell responses. For contrast, we examined *Streptococcus pyogenes*, which undergoes antigenic variation [20], but is not able to persist within a host [21], and we examined three vaccine antigens from *Bacillus anthracis* and *Clostridium tetani.*

We used the computer algorithms NetMHCpan-2.0 and NetMHCIIpan-2.0 to predict the binding affinity of experimentally determined microbial epitopes to HLA-DR, HLA-A, and HLA-B alleles [22,23]. We then analyzed epitope promiscuity across the majority of human HLA alleles, across the most common HLA alleles found in different geographic regions, and across alleles grouped by similar characteristics. We find that epitopes from *B. anthracis* and *C. tetani* are consistently the most promiscuous, and that there is no consistent pattern of promiscuity between *M. tuberculosis*, HIV and *S. pyogenes*. Therefore we find similar levels of epitope promiscuity in very different pathogens, and we discuss the impact of promiscuity on host–pathogen coevolution.

## Methods

### Programs and databases

#### NetMHCpan-2.0

The NetMHCpan-2.0 algorithm (http://www.cbs.dtu.dk/services/NetMHCpan-2.0/) uses an artificial neural network (ANN) to predict the binding affinity of peptide-MHC (HLA)-I molecule interactions [22,23]. Peptide sequence and HLA sequence information were used as input and experimentally determined affinity data from the IEDB database covering 34 HLA-A and 32 HLA-B alleles were used as output to train the ANN. This method currently gives affinity estimates for peptides with 886 HLA-A alleles and 1,412 HLA-B alleles.

#### NetMHCIIpan-2.0

The NetMHCIIpan-2.0 algorithm (http://www.cbs.dtu.dk/services/NetMHCIIpan/) uses an ANN to predict the binding affinity of peptide-MHC (HLA)-II molecule interactions [22,23]. Peptide sequence and HLA sequence information were used as input and experimentally determined affinity data from the IEDB database covering 24 HLA-DR alleles were used as output to train the ANN. This method currently provides binding affinity data for 654 HLA-DR alleles.

#### IEDB

The IEDB (Immune Epitope Database, http://www.immuneepitope.org/) contains peptidic and non-peptidic antibody and T cell epitope data for humans and other animal species. The database contains HLA binding data from diverse antigenic sources, including allergens and multiple human pathogens. It also provides tools for epitope prediction and analysis.

#### HIV Molecular Immunology Database

The HIV Molecular Immunology Database (http://www.hiv.lanl.gov/content/immunology/index.html) contains data on HIV T cell epitopes, and provides access to tools for epitope prediction and analysis.

#### dbMHC

The dbMHC (http://www.ncbi.nlm.nih.gov/projects/gv/mhc/main.fcgi?cmd=init) contains DNA seqeuence and other data related to the human MHC, including anthropology resources regarding the distribution and frequency of specific HLA alleles in multiple populations.

### Alleles and epitopes used in the study

Epitopes were obtained from the IEDB for all bacterial species, and the HIV Molecular Immunology Database for HIV epitopes. All HLA alleles included in netMHCpan and netMHCIIpan were analyzed. The most common alleles in geographic regions were determined using the IEDB population coverage epitope analysis. Alleles were grouped by supertype as described [24]. The list of all alleles, alleles grouped by population region, alleles grouped by supertype, and all epitopes used in this study are provided in Tables S1, S2, S3, and S4.

### Analysis of promiscuity

Binding prediction values were obtained from NetMHCIIpan-2.0 for HLA-DR alleles, and NetMHCpan-2.0 for HLA-A and HLA-B alleles. The two methods give the output binding scores as 1 – log(*aff*), where *aff* is the IC50 value in nM. Thus the scores range from 0 to 1, with higher scores indicating higher affinity. We used a receiver operating characteristic (ROC) curve to obtain a threshold prediction value to specify which epitope/alleles combinations were predicted to interact. To construct the ROC curves we ran NetMHCpan-2.0 and NetMHCIIpan-2.0 against their published validation datasets. We plotted the true positive rate (TPR) against the false positive rate (FPR) at different thresholds of binding from 0 to 1. We chose a FPR of 0.05, which corresponded to thresholds of 0.29 for netMHCpan and 0.585 for netMHCIIpan, and TPRs of 0.89 for HLA class I and 0.24 for HLA class II.

We defined promiscuity as the percent of allotypes each epitope was predicted to bind to at each HLA locus. To analyze overall promiscuity of epitopes from each pathogen species, we calculated the mean promiscuity of each group, and compared them with a one-way ANOVA and Tukey post-test using GraphPad Prism 5. We only included epitopes predicted to bind at least one allele in the analyses. To complement this we performed kernel density estimation using reflection for boundary support [25] to estimate the probability density of epitope promiscuity using MATLAB version 7.13.0.564. This was based on a normal kernel function. The density was evaluated at 101 equally spaced points in the interval [0,100]. The probability distribution of points lying outside the relevant region of [0,100], specifically those in the intervals [-100,0] and [100, 200], was reflected onto the distribution between 0 and 100 to arrive at the complete probability distribution. A bandwidth parameter of 15 was used for smoothing the curves. Distributions shifted to the right of the graph indicate higher promiscuity. Epitope promiscuity was analyzed in this way across all HLA alleles, as well as across HLA alleles grouped by population region and by supertype. Analysis of HLA allele promiscuity was carried out in the same way, with promiscuity defined as the percent of epitopes each allotype was predicted to bind each epitope at each HLA locus.

## Results

### Validation of the netMHCpan and netMHCIIpan methods

The accuracy of the netMHCpan and netMHCIIpan prediction methods has been extensively validated by several previous studies. One study compared experimentally determined peptide-MHC binding affinities with affinities predicted by three different algorithm prediction methods [26]. The authors found a positive correlation between predicted and experimentally determined affinities, and found that this correlation was strongest for the netMHCpan method. Another study evaluated the prediction methods for a larger dataset including alleles that were not included in the set used to train the programs, using a comparison with experimental data from the Immune Epitope Database (IEDB), and showed that the netMHCpan ANN method was top ranking for both affinity and ligand data [27]. In this analysis the authors removed peptides used in program training as well as peptides in the IEDB that had been identified using netMHCpan methods, in order to remove any bias in favor of the netMHCpan methods. An additional study conducted similar, independent evaluations of MHC class I [28] and MHC class II [29] prediction methods, and also found that netMHCpan and netMHCIIpan were consistently the most accurate method when compared with experimental results.

We conducted our own validation studies of the netMHCpan and netMHCIIpan methods by running them against experimental validation datasets. We constructed receiver operating characteristic (ROC) curves for both the methods and confirmed that both methods performed significantly better than random guessing (see Methods). Based on the ROC curve we chose threshold levels for netMHCpan and netMHCIIpan such that the false positive rate (FPR) of detecting epitope binding was 0.05 (Figure S1). This corresponded to a true positive rate (TPR) of 0.89 for HLA class I and 0.24 for HLA class II.

#### HLA alleles and microbe-derived epitopes

We analyzed all HLA-A and HLA-B, and HLA-DR alleles covered by netMHCpan and netMHCIIpan. We analyzed microbe-derived epitopes in four groups: *Bacillus anthracis* and *Clostridium tetani*, *Streptococcus pyogenes*, *Mycobacterium tuberculosis* complex, and Human immunodeficiency virus (HIV). *B. anthracis* and *C. tetani* were grouped together in all analyses because we found no significant differences between them in mean epitope promiscuity across all alleles (data not shown). These groups were selected for their biological diversity as indicated above, and because epitope information was readily available for each in the IEDB. We included all epitopes available for each pathogen in each database at the time of analysis. Supplementary Tables 1 and 2 provide lists of the alleles and epitopes included in this study.

#### Promiscuity across all HLA-DR, -A, -B alleles and epitopes

**Figure 1:**
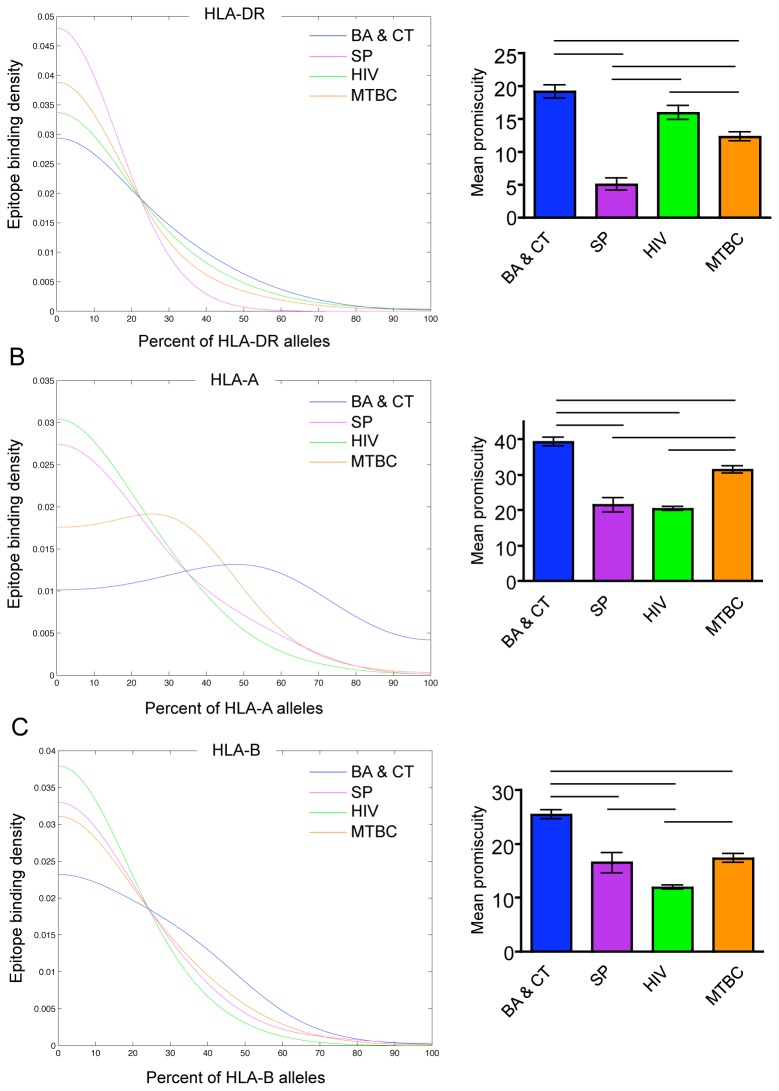
T cell epitope promiscuity across HLA-DR, -A, -B alleles. Kernel density estimates of epitope promiscuity and graphs of mean epitope promiscuity (see Methods) of *B. anthracis* and *C. tetani* (BA & CT; blue), *S. pyogenes* and (SP; purple), HIV (green) and *M. tuberculosis* complex (MTBC; orange) across HLA-DR (A), HLA-A (B) and HLA-B (C) alleles. Significant differences in mean promiscuity between groups are indicated with black bars (Tukey’s post-test, p < 0.05). Error bars represent the standard error of the mean.

To compare promiscuity between groups, we first determined the percent of alleles that each epitope was predicted to bind and computed mean promiscuity of epitopes from each group. This revealed that epitopes in *B. anthracis* and *C. tetani* were most promiscuous across all human HLA-DR, HLA-A and HLA-B alleles (Figure 1), though there was no significant difference in mean promiscuity between *B. anthracis* and *C. tetani* and HIV across HLA-DR alleles (Figure 1A). In addition we plotted distributions of the probability density of epitope binding to increasing percentages of HLA alleles using kernel density estimation (see Methods). Each distribution shifted to the right for *B. anthracis* and *C. tetani*, indicating that these epitopes were more likely to bind a greater breadth of HLA alleles, which was consistent with the mean promiscuity analysis (Figure 1). HIV epitopes were more promiscuous than *M. tuberculosis* and *S. pyogenes* epitopes across HLA-DR alleles (Figure 1A)*. M. tuberculosis* epitopes were more promiscuous than HIV and *S. pyogenes* epitopes across HLA-A alleles by both analyses, and there was no difference between *S. pyogenes* and HIV (Figure 1B). HIV epitopes were less promiscuous than all groups across HLA-B alleles, and there was no significant difference between *M. tuberculosis* and *S. pyogenes* (Figure 1C). We found that using higher or lower threshold values did not alter the results (data not shown). Therefore, while *B. anthracis* and *C. tetani* epitopes were most promiscuous, there was no consistent trend in epitope promiscuity between the other pathogens that extended to multiple HLA loci.

Although we were primarily interested in epitope promiscuity, the study could also have been framed in terms of the ability of HLA molecules to bind multiple epitopes [10]. Therefore we performed the above analyses for predicted HLA allele promiscuity. We found nearly identical trends to those we found for epitope promiscuity using mean promiscuity analysis (Figure S2). Kernel density estimations were also similar (Figure S2). Thus, given the available data we cannot clearly distinguish the effect of epitope promiscuity from HLA allele promiscuity. However, for simplicity, we chose to focus only on epitope promiscuity in all subsequent analyses.

#### Promiscuity across the most common HLA-DR, -A, -B alleles in each geographic region

The majority of the alleles used in the above analysis are found at very low frequencies in the human population. Thus we were interested in whether patterns would be more distinct, or if new patterns would emerge, if we focused on only the alleles most prevalent in specific geographic regions. We grouped populations into Europe and the Americas, East Asia, Sub-Saharan Africa, India and East Africa, and Oceania (Table S3) and analyzed the most prevalent alleles in these regions that comprised 60% of the population. We chose these groups because they follow human migration patterns [30] and, correspondingly, HLA alleles are grouped this way in the dbMHC database. Interestingly, the spread and divergence of pathogens like *M. tuberculosis* also follow these migration patterns [31].

**Figure 2:**
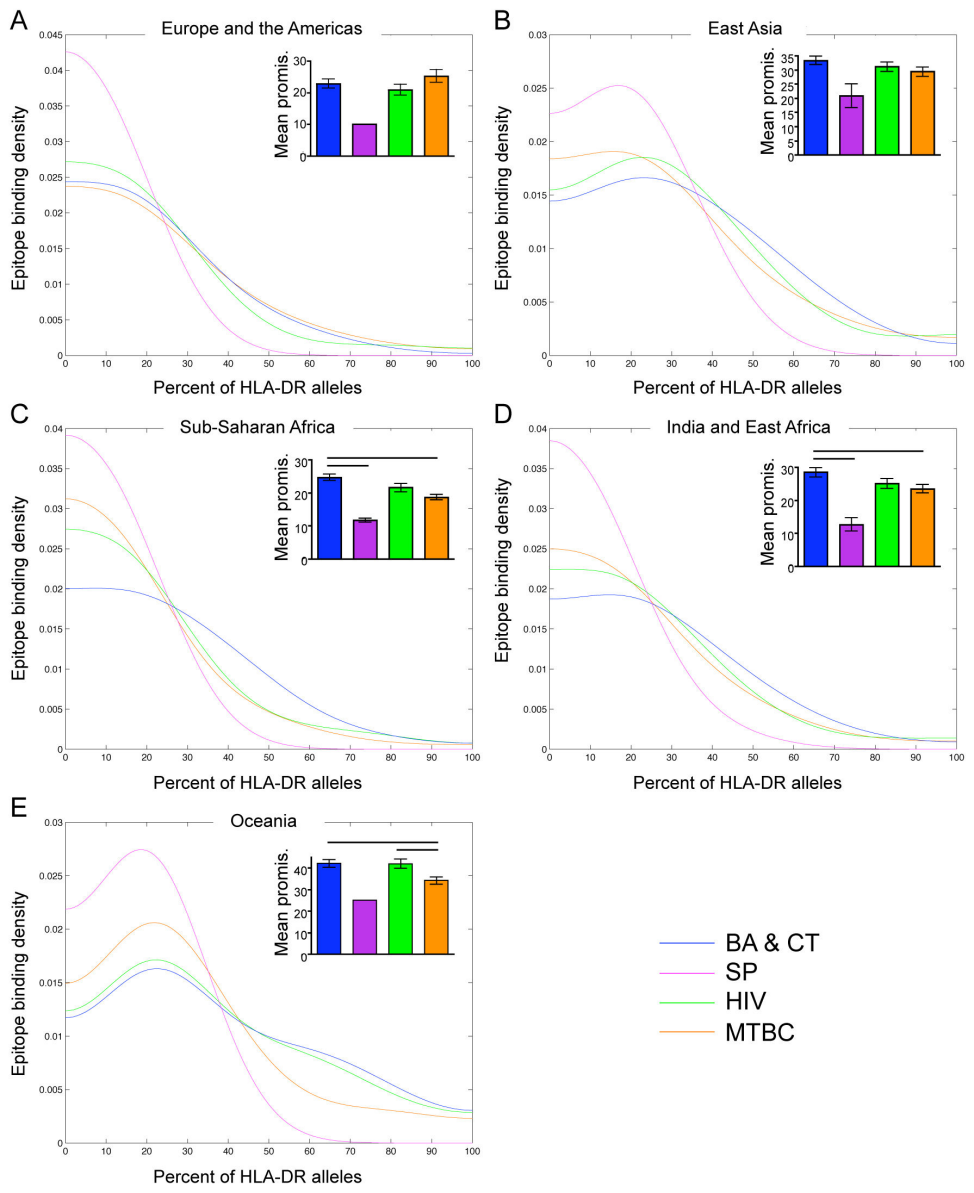
T cell epitope promiscuity across the most common HLA-DR alleles in geographic regions. Kernel density estimates of epitope promiscuity and graphs of mean epitope promiscuity (see Methods) of *B. anthracis* and *C. tetani* (BA & CT; blue), *S. pyogenes* and (SP; purple), HIV (green) and *M. tuberculosis* complex (MTBC; orange) across the most common HLA-DR alleles in Europe and the Americas (A), East Asia (B), Sub-Saharan Africa (C), India and East Africa (D) and Oceania (E). Significant differences in mean promiscuity between groups are indicated with black bars (Tukey’s post-test, p < 0.05). Error bars represent the standard error of the mean.

**Figure 3:**
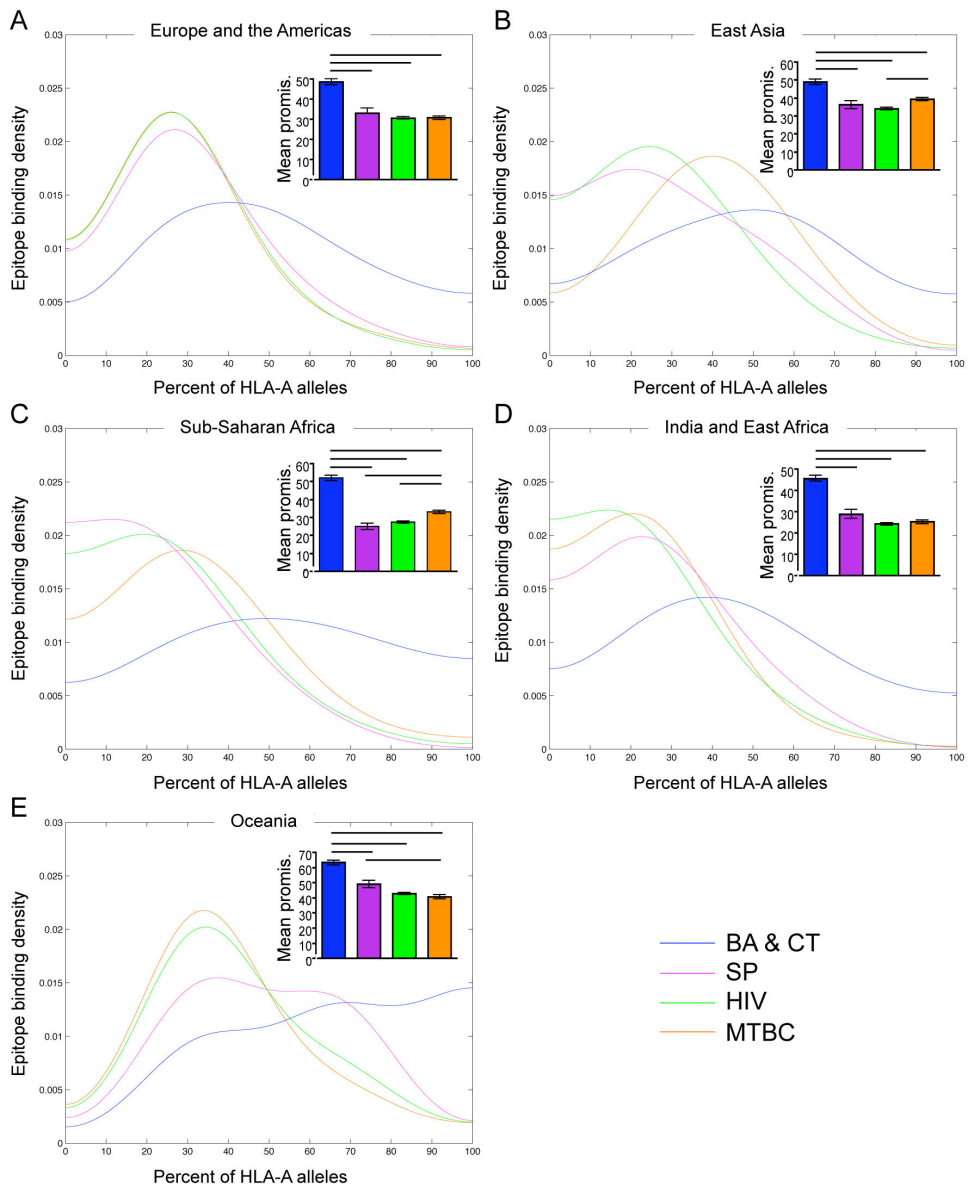
T cell epitope promiscuity across the most common HLA-A alleles in geographic regions. Kernel density estimates of epitope promiscuity and graphs of mean epitope promiscuity (see Methods) of *B. anthracis* and *C. tetani* (BA & CT; blue), *S. pyogenes* and (SP; purple), HIV (green) and *M. tuberculosis* complex (MTBC; orange) across the most common HLA-A alleles in Europe and the Americas (A), East Asia (B), Sub-Saharan Africa (C), India and East Africa (D) and Oceania (E). Significant differences in mean promiscuity between groups are indicated with black bars (Tukey’s post-test, p < 0.05). Error bars represent the standard error of the mean.

**Figure 4:**
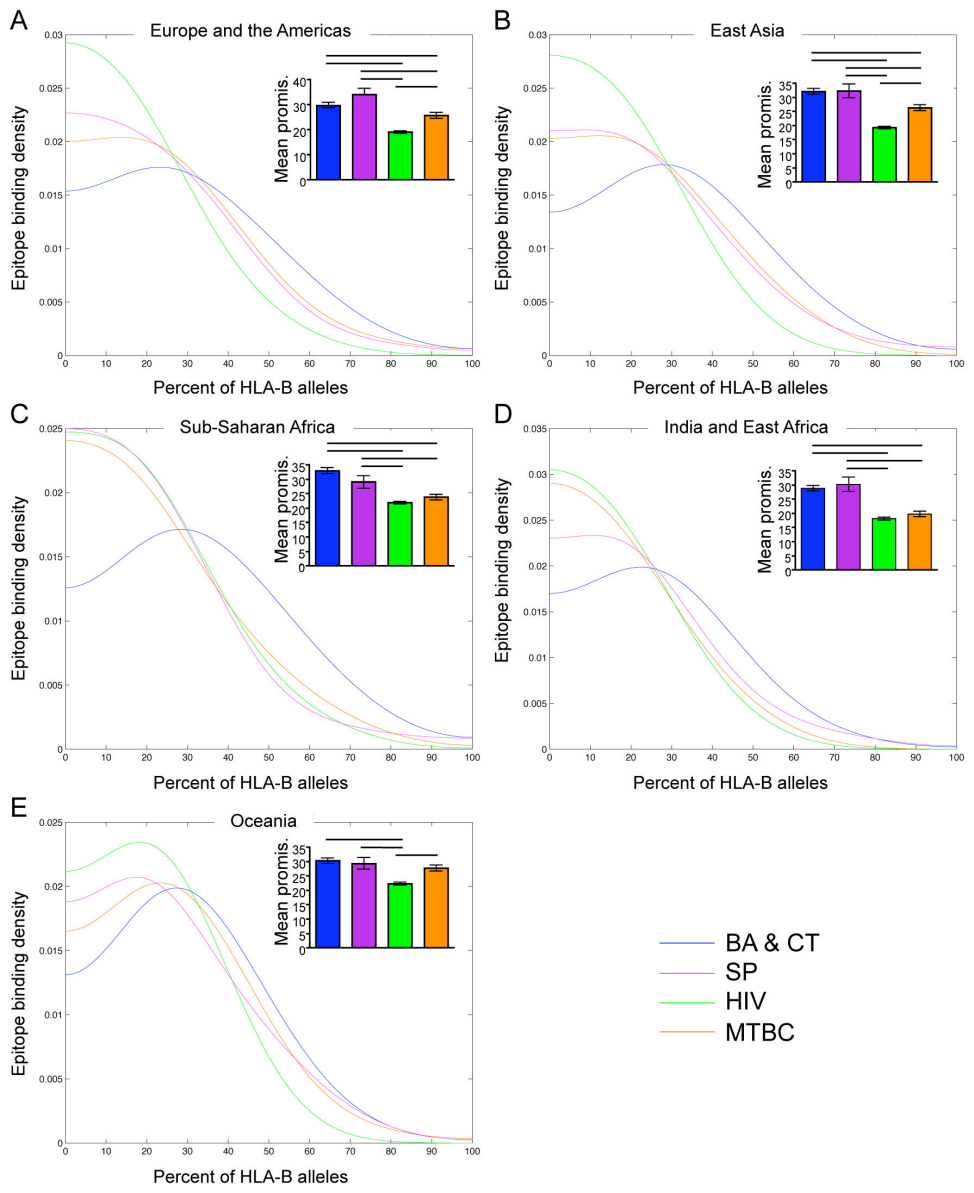
T cell epitope promiscuity across the most common HLA-B alleles in geographic regions. Kernel density estimates of epitope promiscuity and graphs of mean epitope promiscuity (see Methods) of *B. anthracis* and *C. tetani* (BA & CT; blue), *S. pyogenes* (SP; purple), HIV (green) and *M. tuberculosis* complex (MTBC; orange) across the most common HLA-B alleles in Europe and the Americas (A), East Asia (B), Sub-Saharan Africa (C), India and East Africa (D) and Oceania (E). Significant differences in mean promiscuity between groups are indicated with black bars (Tukey’s post-test, p < 0.05). Error bars represent the standard error of the mean.

We found that patterns of promiscuity within geographic regions (Figures 2-4) were similar, though not identical, to those found across all alleles (Figure 1)*. B. anthracis* and *C. tetani* epitopes were most promiscuous across HLA-DR alleles by both analyses in Sub-Saharan African and India and East Africa (Figure 2C-D)*. S. pyogenes* epitopes were least promiscuous in all regions, although the differences in mean promiscuity were not statistically significant for all pair wise comparisons (Figure 2A–E). There was no striking difference in epitope promiscuity across HLA-DR alleles between *M. tuberculosis* and HIV in all regions except Oceania, where HIV epitopes were more promiscuous (Figure 2A–E). We found that *B. anthracis* and *C. tetani* epitopes were most promiscuous across HLA-A alleles in all geographic regions (Figure 3A–E). The other three groups had similar levels of promiscuity in all regions. *M. tuberculosis* epitopes were more promiscuous in East Asia and Sub-Saharan Africa (Figure 3B,C), and *S. pyogenes* epitopes were more promiscuous in Oceania (Figure 3E). *B. anthracis* and *C. tetani* and *S. pyogenes* epitopes were most promiscuous across HLA-B alleles in all regions, though this trend was not distinct for *S. pyogenes* by the Kernel density estimation (Figure 4A–E). *M. tuberculosis* epitopes were more promiscuous than HIV epitopes in Europe and the Americas, East Asia, and Oceania (Figure 4A,B,E), however there was no striking difference between *M. tuberculosis* and HIV in Sub-Saharan Africa and India and East Africa (Figure 4C,D).

#### Promiscuity within allele supertypes

Despite being highly polymorphic, HLA alleles can be organized into groups that retain similar characteristics with overlapping peptide-binding repertoires, known as supertypes [24]. Supertypes were not specific to population region, as we found a diverse collection of supertypes in each of the allele groups used in the population analysis (data not shown). Thus, we looked at whether there would be trends in epitope promiscuity within groups of alleles with similar characteristics. Supertypes have been best defined for HLA class I alleles [24], therefore for this analysis we focused on HLA-A and HLA-B (Table S4). Using mean promiscuity analysis, we found that *B. anthracis* and *C. tetani* were most promiscuous within three of four HLA-A supertypes, and that there was no trend between *S. pyogenes*, HIV and *M. tuberculosis* (Figure S3). There was also no striking trend between any of the pathogen groups within HLA-B supertypes (Figure S3).

## Discussion

In this study we compared T cell epitope promiscuity between ecologically diverse pathogens with different antigenic diversities. Epitope promiscuity has been shown both experimentally and computationally for peptides derived from different pathogens, including the human papillomavirus [16], HIV [11,15,17,18], and *M. tuberculosis* [14,32], among others [9,10,12,13]. However, to our knowledge, this is the first study that directly compares promiscuity in multiple pathogens. We found that *B. anthracis* and *C. tetani* were the most promiscuous epitope group in the majority of the analyses, and we found no trend in epitope promiscuity between HIV, *M. tuberculosis* and *S. pyogenes*. These latter pathogens have extremely different infection strategies and life cycles; thus if there were marked differences in promiscuity between species they should have been detected by our analyses. Importantly, there is no established standard for epitope promiscuity that allows determination whether groups of epitopes are highly promiscuous or not.

The impact of epitope promiscuity on the outcomes of host–pathogen interactions remains to be determined. Promiscuity could benefit the host by increasing the number of antigenic peptides that HLA molecules can present to T cells. This large pool of peptides could also allow for selection of immunodominant peptides that elicit the strongest T cell responses [33]. If this is the case, vaccines containing promiscuous epitopes should be especially effective; and this has been suggested by several studies [34-38]. Our results also showed that epitopes derived from successful vaccine targets, *B. anthracis* and *C. tetani* epitopes, were the most promiscuous. Alternatively, peptides recognized by many HLA molecules in a population could predispose to immune exhaustion and transmission of pathogens pre-adapted to the immune response. In this context, promiscuity would be harmful to the host. A recent study of HIV found that HLA molecules associated with slow disease progression also had the lowest levels of promiscuity [39]. The authors propose that carrying HLA molecules with promiscuous binding repertoires makes an individual “functionally homozygous” at the HLA locus, and therefore decreases the heterozygote advantage of having greater HLA allele diversity.

An unexpected finding in the present study is that conserved *M. tuberculosis* epitopes and variable HIV epitopes have similar breadths of HLA binding. Pathogens evolve variable epitopes because they are less likely to be recognized by one or more of the diverse HLA alleles [5,6]. Therefore, one explanation for the evolution of hyperconserved epitopes is that *M. tuberculosis* actually benefits from recognition [8]. If promiscuity enhances the host’s ability to recognize pathogens, then we might have expected *M. tuberculosis* epitopes to be more promiscuous than HIV or *S. pyogenes* epitopes. As this was not the case, we consider the idea that promiscuity does not globally enhance immune recognition because it diminishes the heterozygote advantage [39]. Whether pathogens benefit from recognition or aim to avoid recognition, they are evolving to exploit the host, presumably to the detriment of the host. Thus, regardless of infection strategy, antagonistic coevolution could emerge: the host benefits from generating new HLA allele combinations that the pathogen has not yet adapted to, and the pathogen benefits from evolving a way to diminish the new HLA diversity. In this manner *M. tuberculosis*, HIV, and *S. pyogenes* could have similar extents of promiscuity because each benefits in a similar manner from promiscuous epitopes.

Interestingly, although immune evasion through antigenic variation is the main strategy used by HIV, it has recently been shown that a subset of HIV epitopes is selectively hyperconserved [40]. Similarly, while the majority of identified *M. tuberculosis* epitopes are highly conserved, a small subset of *M. tuberculosis* epitopes is variable [8]. It has been proposed that recognition of specific subsets of epitopes may benefit the host or the pathogens during specific stages of the infection. Thus, it is tempting to speculate that epitope promiscuity is a characteristic associated with specific subsets of epitopes – e.g. the epitopes promoting pathogen virulence. Testing this hypothesis by focusing our analysis on these sub-groups would be the next step to determine whether promiscuity depends on the conservation of epitopes or on the virulence cycle of the pathogen.

The advantage of using algorithm-based methods was that we were able to generate data for almost any epitope-HLA combination. In this study we were primarily interested in the potential for different epitopes to bind a diverse array of HLA alleles, and any inconsistencies between the algorithm predictions and biological reality will have applied equally to each pathogen group. In vitro binding assays can be used to complement computational methods by directly testing the ability of epitopes to compete with other peptides known to bind HLA molecules, however there are also limits to computer-based and in vitro systems. Peptides that bind most strongly to HLA molecules do not necessarily elicit the strongest T cell responses [9,41], and thus these methods are not infallible for identifying immunodominant peptides. Techniques that facilitate further discovery of the determinants of peptide epitope generation and recognition, together with the kinetics of epitope generation and presentation during the course of infection [42], will have a major impact on understanding the impact of promiscuity on infection.

## Conclusions

We used computer-based algorithms to compare the ability of epitopes from a variety of pathogens to bind multiple of the diverse HLA alleles. We found similar levels of epitope promiscuity in HIV, *M. tuberculosis* and *S. pyogenes*, despite differences in biology and epitope diversity. We propose a model where promiscuity benefits a broad range of successful human pathogens because it decreases the functional diversity of HLA alleles in the human population. Studies that examine the binding repertoire of epitopes presented at different stages of human infection will be important to further our understanding of how promiscuity influences host–pathogen interaction, and how it could be incorporated into vaccine design or disease management.

## Supporting Information

Figure S1 Receiver operator characteristic (**ROC**) curves for netMHCpan and netMHCIIpan. ROC curves were generated by running NetMHCpan-2.0 and NetMHCIIpan-2.0 against their published validation datasets. The true positive rate (TPR) is plotted against the false positive rate (FPR) at different thresholds of binding from 0 to 1. For subsequent analyses we chose a FPR of 0.05, which corresponded to thresholds of 0.29 for netMHCpan and 0.585 for netMHCIIpan, and TPRs of 0.89 for HLA class I and 0.24 for HLA class II.
(TIF)

Figure S2 HLA-DR, -A, -B allele promiscuity across all epitopes. Kernel density estimates of epitope promiscuity and graphs of mean HLA allele promiscuity (see Methods) of *B. anthracis* and *C. tetani* (BA & CT; blue), *S. pyogenes* and (SP; purple), HIV (green) and *M. tuberculosis* complex (MTBC; orange) across HLA-DR (A), HLA-A (B) and HLA-B (C) alleles. Differences in mean promiscuity are indicated with black bars (Tukey’s post-test, p < 0.05). Error bars represent the standard error of the mean.
(TIF)

Figure S3 T cell epitope promiscuity across HLA class I alleles grouped by supertype. Graphs of mean epitope promiscuity (see Methods) of *B. anthracis* and *C. tetani* (BA and CT), *S. pyogenes* and (SP), HIV and *M. tuberculosis* complex (MTBC) across alleles within HLA-A supertypes (A01, A02, A03, A24) and within HLA-B supertypes (B07, B08, B27, B44, B58, B62). For simplicity, Tukey post-tests are not shown because no trend was found between the groups.
(TIF)

Table S1 All epitopes used in this study. The first column refers to the IEDB reference number for bacterial epitopes, and amino acid sequence for HIV epitopes. The second column refers to the microbe group from which the epitope was derived. The third column refers to the protein from which the epitope was derived. The fourth column refers to the class of HLA alleles that the epitopes have been shown to bind, and the class in which they were analyzed in this study.
(PDF)

Table S2 All HLA-DR, -A, -B alleles used in this study. (PDF)

Table S3 Alleles grouped by population region. (PDF)

Table S4 Alleles grouped by supertype. (PDF)
