# Supplementary figures and images for: Equivalent T Cell Epitope Promiscuity in Ecologically Diverse Human Pathogens
Source: PLoS One. 2013 Aug 9;8(8):e73124. doi: 10.1371/journal.pone.0073124 (PMC3739752; doi:10.1371/journal.pone.0073124)

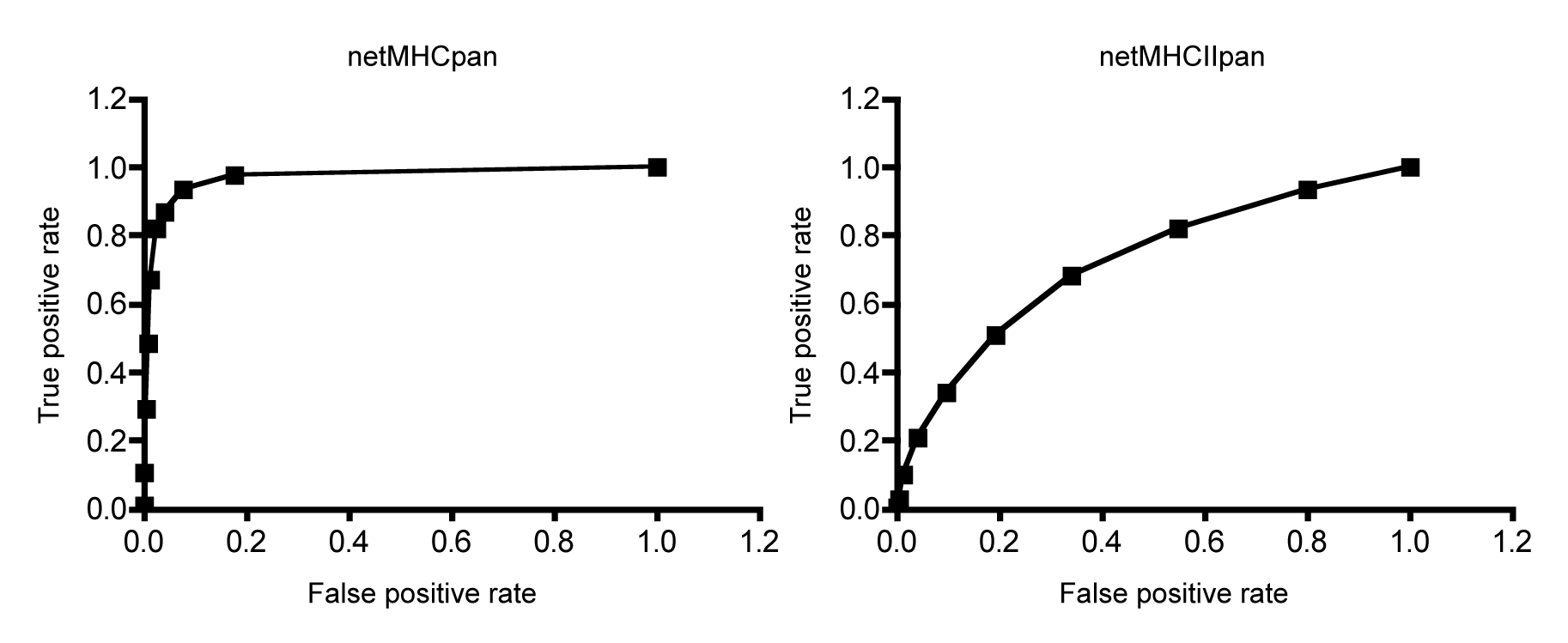

Supplement: Figure S1 — ROC curves were generated by running NetMHCpan-2.0 and NetMHCIIpan-2.0 against their published validation datasets. The true positive rate (TPR) is plotted against the false positive rate (FPR) at different thresholds of binding from 0 to 1. For subsequent analyses we chose a FPR of 0.05, which corresponded to thresholds of 0.29 for netMHCpan and 0.585 for netMHCIIpan, and TPRs of 0.89 for HLA class I and 0.24 for HLA class II. (TIF) [file pone.0073124.s001.tif]

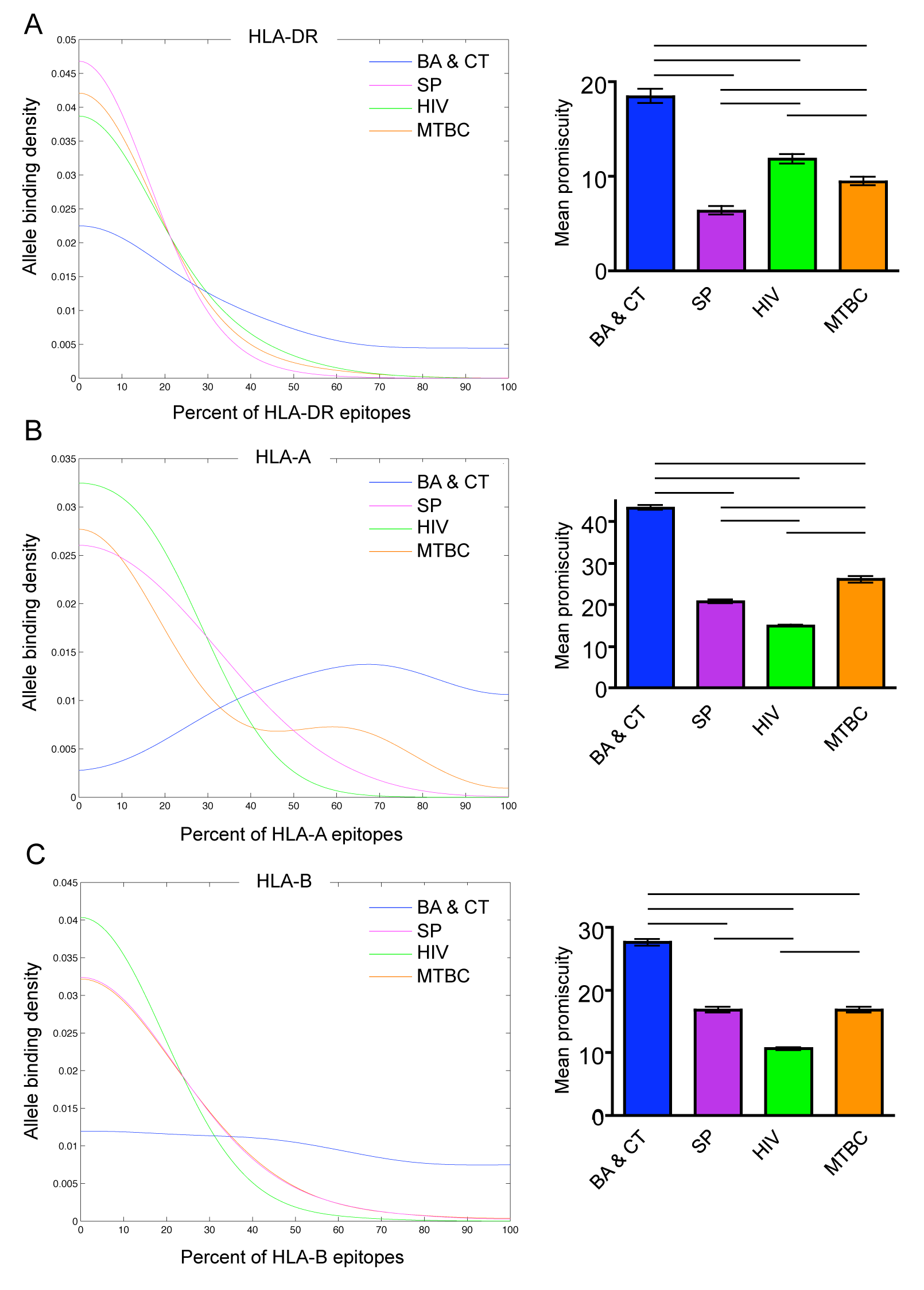

Supplement: Figure S2 — Kernel density estimates of epitope promiscuity and graphs of mean HLA allele promiscuity (see Methods) of B. anthracis and C. tetani (BA & CT; blue), S. pyogenes and (SP; purple), HIV (green) and M. tuberculosis complex (MTBC; orange) across HLA-DR (A), HLA-A (B) and HLA-B (C) alleles. Differences in mean promiscuity are indicated with black bars (Tukey’s post-test, p < 0.05). Error bars represent the standard error of the mean. (TIF) [file pone.0073124.s002.tif]

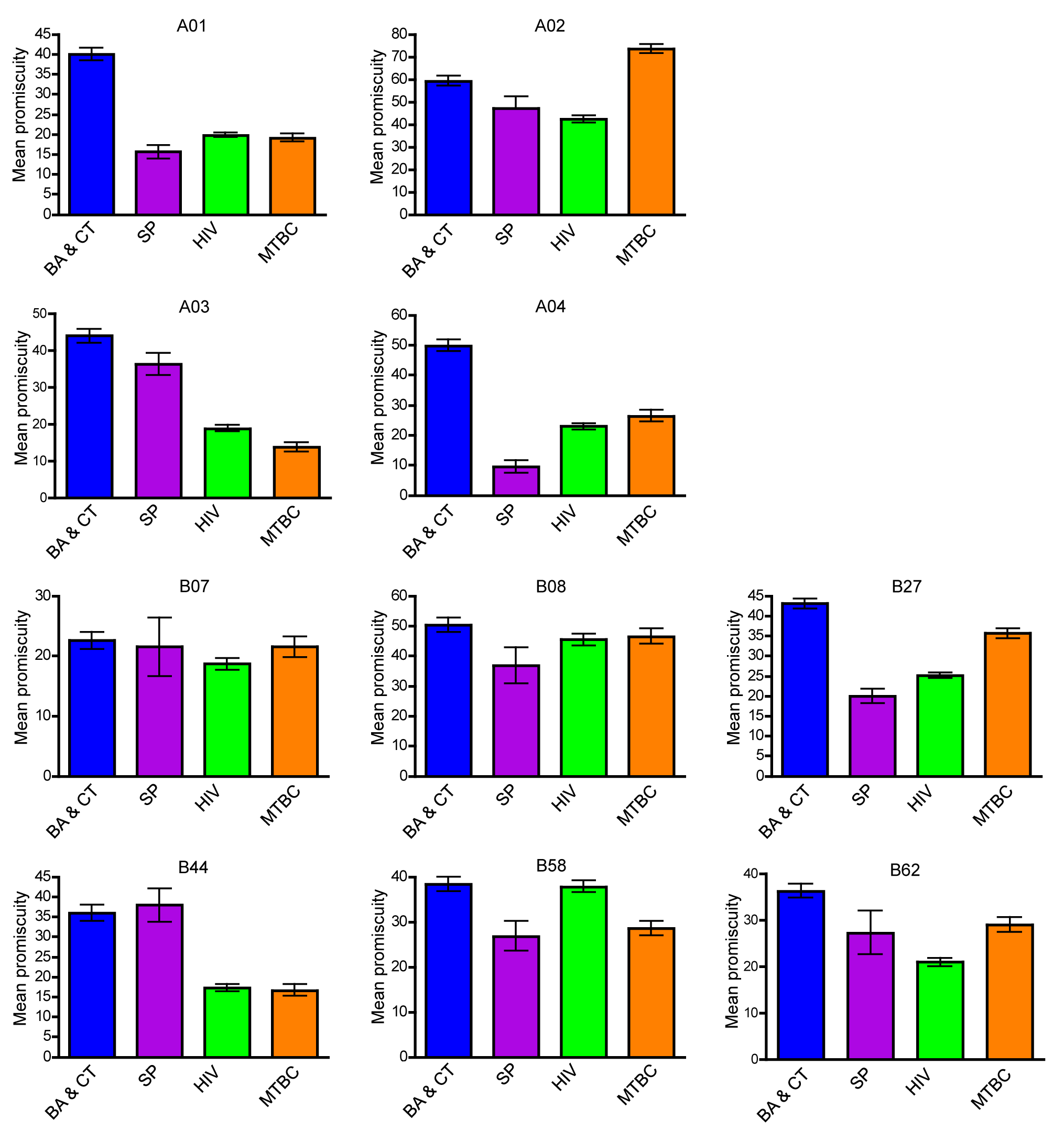

Supplement: Figure S3 — Graphs of mean epitope promiscuity (see Methods) of B. anthracis and C. tetani (BA and CT), S. pyogenes and (SP), HIV and M. tuberculosis complex (MTBC) across alleles within HLA-A supertypes (A01, A02, A03, A24) and within HLA-B supertypes (B07, B08, B27, B44, B58, B62). For simplicity, Tukey post-tests are not shown because no trend was found between the groups. (TIF) [file pone.0073124.s003.tif]
