# Supplementary material for: Equivalent T Cell Epitope Promiscuity in Ecologically Diverse Human Pathogens
Source: PLoS One. 2013 Aug 9;8(8):e73124. doi: 10.1371/journal.pone.0073124 (PMC3739752; doi:10.1371/journal.pone.0073124)
Supplement: Table S1 — The first column refers to the IEDB reference number for bacterial epitopes, and amino acid sequence for HIV epitopes. The second column refers to the microbe group from which the epitope was derived. The third column refers to the protein from which the epitope was derived. The fourth column refers to the class of HLA alleles that the epitopes have been shown to bind, and the class in which they were analyzed in this study. (PDF) [file pone.0073124.s004.pdf]

Epitope	Source microbe	Source protein	HLA class
19039	Bacillus anthracis	protective antigen	I, II
22071	Bacillus anthracis	protective antigen	I, II
23488	Bacillus anthracis	protective antigen	I, II
25594	Bacillus anthracis	protective antigen	I, II
37302	Bacillus anthracis	Lethal factor precursor	I, II
38653	Bacillus anthracis	protective antigen	I, II
43228	Bacillus anthracis	protective antigen	I, II
44523	Bacillus anthracis	protective antigen	I, II
48019	Bacillus anthracis	Protective antigen precursor	I, II
54845	Bacillus anthracis	Protective antigen precursor	I, II
57309	Bacillus anthracis	Lethal factor precursor	I, II
57667	Bacillus anthracis	protective antigen	I, II
59556	Bacillus anthracis	Protective antigen precursor	I, II
62947	Bacillus anthracis	protective antigen	I, II
68329	Bacillus anthracis	protective antigen	I, II
74023	Bacillus anthracis	Lethal factor precursor	I, II
75137	Bacillus anthracis	protective antigen	I, II
76444	Bacillus anthracis	Lethal factor precursor	I, II
76497	Bacillus anthracis	Lethal factor precursor	I, II
79759	Bacillus anthracis	protective antigen precursor	I, II
79762	Bacillus anthracis	protective antigen precursor	I, II
79791	Bacillus anthracis	protective antigen precursor	I, II
79824	Bacillus anthracis	protective antigen precursor	I, II
79832	Bacillus anthracis	protective antigen precursor	I, II
79862	Bacillus anthracis	protective antigen precursor	I, II
79865	Bacillus anthracis	protective antigen precursor	I, II
79872	Bacillus anthracis	protective antigen precursor	I, II
79882	Bacillus anthracis	protective antigen precursor	I, II
79884	Bacillus anthracis	protective antigen precursor	I, II
79897	Bacillus anthracis	protective antigen precursor	I, II
79902	Bacillus anthracis	protective antigen precursor	I, II
79907	Bacillus anthracis	protective antigen precursor	I, II
79944	Bacillus anthracis	protective antigen precursor	I, II
79951	Bacillus anthracis	protective antigen precursor	I, II
79956	Bacillus anthracis	protective antigen precursor	I, II
79973	Bacillus anthracis	protective antigen precursor	I, II
79990	Bacillus anthracis	protective antigen precursor	I, II
80026	Bacillus anthracis	protective antigen precursor	I, II
80038	Bacillus anthracis	protective antigen precursor	I, II
80046	Bacillus anthracis	protective antigen precursor	I, II
80053	Bacillus anthracis	protective antigen precursor	I, II
80107	Bacillus anthracis	protective antigen precursor	I, II
80549	Bacillus anthracis	Lethal factor precursor	I, II
81065	Bacillus anthracis	Lethal factor	I, II
81391	Bacillus anthracis	Lethal factor precursor	I, II
81557	Bacillus anthracis	Lethal factor precursor	I, II
81794	Bacillus anthracis	Lethal factor precursor	I, II
81917	Bacillus anthracis	Lethal factor precursor	I, II
83206	Bacillus anthracis	Lethal factor precursor	I, II
83606	Bacillus anthracis	Lethal factor	I, II
83769	Bacillus anthracis	Lethal factor precursor	I, II
84202	Bacillus anthracis	Lethal factor	I, II
84412	Bacillus anthracis	Lethal factor precursor	I, II
84479	Bacillus anthracis	Lethal factor precursor	I, II
84775	Bacillus anthracis	Lethal factor precursor	I, II
85064	Bacillus anthracis	Lethal factor precursor	I, II
85110	Bacillus anthracis	Lethal factor	I, II
85311	Bacillus anthracis	Lethal factor precursor	I, II
86386	Bacillus anthracis	lethal factor precursor	I, II
86694	Bacillus anthracis	lethal factor precursor	I, II
86808	Bacillus anthracis	Lethal factor precursor	I, II
87309	Bacillus anthracis	Lethal factor precursor	I, II
87624	Bacillus anthracis	Lethal factor precursor	I, II
87648	Bacillus anthracis	Lethal factor precursor	I, II
87698	Bacillus anthracis	Lethal factor precursor	I, II
89938	Bacillus anthracis	Lethal factor precursor	I, II
90078	Bacillus anthracis	Lethal factor precursor	I, II
90174	Bacillus anthracis	Lethal factor precursor	I, II
90856	Bacillus anthracis	Lethal factor precursor	I, II
96057	Bacillus anthracis	Lethal factor precursor	I, II
96073	Bacillus anthracis	protective antigen	I, II
96181	Bacillus anthracis	Lethal factor precursor	I, II

96187	Bacillus anthracis	protective antigen	I, II
96196	Bacillus anthracis	Lethal factor precursor	I, II
96218	Bacillus anthracis	Lethal factor precursor	I, II
96236	Bacillus anthracis	Lethal factor precursor	I, II
96252	Bacillus anthracis	protective antigen	I, II
96265	Bacillus anthracis	Lethal factor precursor	I, II
96266	Bacillus anthracis	Lethal factor precursor	I, II
96301	Bacillus anthracis	Lethal factor precursor	I, II
96326	Bacillus anthracis	protective antigen	I, II
96378	Bacillus anthracis	Lethal factor precursor	I, II
96397	Bacillus anthracis	protective antigen	I, II
96472	Bacillus anthracis	Lethal factor precursor	I, II
96604	Bacillus anthracis	protective antigen	I, II
96613	Bacillus anthracis	Lethal factor precursor	I, II
96644	Bacillus anthracis	Lethal factor precursor	I, II
96693	Bacillus anthracis	Lethal factor precursor	I, II
96732	Bacillus anthracis	protective antigen	I, II
96774	Bacillus anthracis	Lethal factor precursor	I, II
96794	Bacillus anthracis	protective antigen	I, II
96796	Bacillus anthracis	protective antigen	I, II
96810	Bacillus anthracis	protective antigen	I, II
96865	Bacillus anthracis	Lethal factor precursor	I, II
96883	Bacillus anthracis	Lethal factor precursor	I, II
96922	Bacillus anthracis	Lethal factor precursor	I, II
97027	Bacillus anthracis	protective antigen	I, II
97047	Bacillus anthracis	protective antigen	I, II
125850	Bacillus anthracis	Lethal factor precursor	I, II
125887	Bacillus anthracis	Lethal factor precursor	I, II
126055	Bacillus anthracis	Lethal factor precursor	I, II
126615	Bacillus anthracis	Lethal factor precursor	I, II
126814	Bacillus anthracis	Lethal factor precursor	I, II
126848	Bacillus anthracis	Lethal factor precursor	I, II
127230	Bacillus anthracis	Lethal factor precursor	I, II
127413	Bacillus anthracis	Lethal factor precursor	I, II
1270	Clostridium tetani	Tetanus toxin precursor	I, II
1389	Clostridium tetani	Tetanus toxin precursor	I, II
1501	Clostridium tetani	Tetanus toxin precursor	I, II
1929	Clostridium tetani	Tetanus toxin precursor	I, II
2219	Clostridium tetani	Tetanus toxin precursor	I, II
3156	Clostridium tetani	Tetanus toxin precursor	I, II
3418	Clostridium tetani	Tetanus toxin precursor	I, II
3832	Clostridium tetani	Tetanus toxin precursor	I, II
4449	Clostridium tetani	Tetanus toxin precursor	I, II
5407	Clostridium tetani	Tetanus toxin precursor	I, II
7603	Clostridium tetani	Tetanus toxin precursor	I, II
8658	Clostridium tetani	Tetanus toxin precursor	I, II
8659	Clostridium tetani	Tetanus toxin precursor	I, II
8734	Clostridium tetani	Tetanus toxin precursor	I, II
8778	Clostridium tetani	Tetanus toxin precursor	I, II
8895	Clostridium tetani	Tetanus toxin precursor	I, II
8903	Clostridium tetani	Tetanus toxin precursor	I, II
9297	Clostridium tetani	Tetanus toxin precursor	I, II
9595	Clostridium tetani	Tetanus toxin precursor	I, II
9710	Clostridium tetani	Tetanus toxin precursor	I, II
9716	Clostridium tetani	Tetanus toxin precursor	I, II
10365	Clostridium tetani	Tetanus toxin precursor	I, II
10472	Clostridium tetani	Tetanus toxin precursor	I, II
10743	Clostridium tetani	Tetanus toxin precursor	I, II
11710	Clostridium tetani	Tetanus toxin precursor	I, II
12717	Clostridium tetani	Tetanus toxin precursor	I, II
13078	Clostridium tetani	Tetanus toxin precursor	I, II
13095	Clostridium tetani	Tetanus toxin precursor	I, II
13125	Clostridium tetani	Tetanus toxin precursor	I, II
13813	Clostridium tetani	Tetanus toxin precursor	I, II
14276	Clostridium tetani	Tetanus toxin precursor	I, II
14562	Clostridium tetani	Tetanus toxin precursor	I, II
15411	Clostridium tetani	Tetanus toxin precursor	I, II
16873	Clostridium tetani	Tetanus toxin precursor	I, II
17134	Clostridium tetani	Tetanus toxin precursor	I, II
17193	Clostridium tetani	Tetanus toxin precursor	I, II
17205	Clostridium tetani	Tetanus toxin precursor	I, II
17206	Clostridium tetani	Tetanus toxin	I, II
17207	Clostridium tetani	Tetanus toxin precursor	I, II

17211	Clostridium tetani	Tetanus toxin precursor	I, II
17487	Clostridium tetani	Tetanus toxin precursor	I, II
18356	Clostridium tetani	Tetanus toxin precursor	I, II
18657	Clostridium tetani	Tetanus toxin precursor	I, II
20402	Clostridium tetani	Tetanus toxin precursor	I, II
20505	Clostridium tetani	Tetanus toxin precursor	I, II
21889	Clostridium tetani	Tetanus toxin precursor	I, II
22769	Clostridium tetani	Tetanus toxin precursor	I, II
24238	Clostridium tetani	Tetanus toxin precursor	I, II
25550	Clostridium tetani	Tetanus toxin precursor	I, II
25597	Clostridium tetani	Tetanus toxin precursor	I, II
25666	Clostridium tetani	Tetanus toxin precursor	I, II
26448	Clostridium tetani	Tetanus toxin precursor	I, II
26808	Clostridium tetani	Tetanus toxin precursor	I, II
26851	Clostridium tetani	Tetanus toxin precursor	I, II
27043	Clostridium tetani	Tetanus toxin precursor	I, II
27210	Clostridium tetani	Tetanus toxin precursor	I, II
27639	Clostridium tetani	Tetanus toxin precursor	I, II
27951	Clostridium tetani	Tetanus toxin precursor	I, II
28866	Clostridium tetani	Tetanus toxin precursor	I, II
28917	Clostridium tetani	Tetanus toxin precursor	I, II
28983	Clostridium tetani	Tetanus toxin precursor	I, II
29241	Clostridium tetani	Tetanus toxin precursor	I, II
29331	Clostridium tetani	Tetanus toxin precursor	I, II
29407	Clostridium tetani	Tetanus toxin precursor	I, II
29408	Clostridium tetani	Tetanus toxin precursor	I, II
29697	Clostridium tetani	Tetanus toxin precursor	I, II
29733	Clostridium tetani	Tetanus toxin precursor	I, II
29843	Clostridium tetani	Tetanus toxin precursor	I, II
30269	Clostridium tetani	Tetanus toxin precursor	I, II
30572	Clostridium tetani	Tetanus toxin precursor	I, II
30742	Clostridium tetani	Tetanus toxin precursor	I, II
31286	Clostridium tetani	Tetanus toxin precursor	I, II
31366	Clostridium tetani	Tetanus toxin precursor	I, II
31483	Clostridium tetani	Tetanus toxin precursor	I, II
31484	Clostridium tetani	Tetanus toxin precursor	I, II
31570	Clostridium tetani	Tetanus toxin precursor	I, II
32521	Clostridium tetani	Tetanus toxin precursor	I, II
33449	Clostridium tetani	Tetanus toxin precursor	I, II
34301	Clostridium tetani	Tetanus toxin precursor	I, II
35058	Clostridium tetani	Tetanus toxin precursor	I, II
35064	Clostridium tetani	Tetanus toxin precursor	I, II
35566	Clostridium tetani	Tetanus toxin precursor	I, II
35767	Clostridium tetani	Tetanus toxin precursor	I, II
35768	Clostridium tetani	Tetanus toxin precursor	I, II
35993	Clostridium tetani	Tetanus toxin precursor	I, II
36057	Clostridium tetani	Tetanus toxin precursor	I, II
36647	Clostridium tetani	Tetanus toxin precursor	I, II
36667	Clostridium tetani	Tetanus toxin precursor	I, II
36852	Clostridium tetani	Tetanus toxin precursor	I, II
37016	Clostridium tetani	Tetanus toxin precursor	I, II
37048	Clostridium tetani	Tetanus toxin precursor	I, II
37163	Clostridium tetani	Tetanus toxin precursor	I, II
38107	Clostridium tetani	Tetanus toxin precursor	I, II
38109	Clostridium tetani	Tetanus toxin precursor	I, II
38755	Clostridium tetani	Tetanus toxin precursor	I, II
38977	Clostridium tetani	Tetanus toxin precursor	I, II
39047	Clostridium tetani	Tetanus toxin precursor	I, II
39248	Clostridium tetani	Tetanus toxin precursor	I, II
40513	Clostridium tetani	Tetanus toxin precursor	I, II
40770	Clostridium tetani	Tetanus toxin precursor	I, II
41527	Clostridium tetani	Tetanus toxin precursor	I, II
41627	Clostridium tetani	Tetanus toxin precursor	I, II
42445	Clostridium tetani	Tetanus toxin precursor	I, II
42847	Clostridium tetani	Tetanus toxin precursor	I, II
43519	Clostridium tetani	Tetanus toxin precursor	I, II
43591	Clostridium tetani	Tetanus toxin precursor	I, II
43939	Clostridium tetani	Tetanus toxin precursor	I, II
43940	Clostridium tetani	Tetanus toxin precursor	I, II
44056	Clostridium tetani	Tetanus toxin precursor	I, II
44200	Clostridium tetani	Tetanus toxin precursor	I, II
44284	Clostridium tetani	Tetanus toxin precursor	I, II
44383	Clostridium tetani	Tetanus toxin precursor	I, II

44396	Clostridium tetani	Tetanus toxin precursor	I, II
44557	Clostridium tetani	Tetanus toxin precursor	I, II
44667	Clostridium tetani	Tetanus toxin precursor	I, II
45102	Clostridium tetani	Tetanus toxin precursor	I, II
45137	Clostridium tetani	Tetanus toxin precursor	I, II
45184	Clostridium tetani	Tetanus toxin precursor	I, II
45993	Clostridium tetani	Tetanus toxin precursor	I, II
46005	Clostridium tetani	Tetanus toxin precursor	I, II
46136	Clostridium tetani	Tetanus toxin precursor	I, II
46613	Clostridium tetani	Tetanus toxin precursor	I, II
46701	Clostridium tetani	Tetanus toxin precursor	I, II
46853	Clostridium tetani	Tetanus toxin precursor	I, II
46855	Clostridium tetani	Tetanus toxin precursor	I, II
47312	Clostridium tetani	Tetanus toxin precursor	I, II
47412	Clostridium tetani	Tetanus toxin precursor	I, II
47681	Clostridium tetani	Tetanus toxin precursor	I, II
47945	Clostridium tetani	Tetanus toxin precursor	I, II
47999	Clostridium tetani	Tetanus toxin precursor	I, II
48049	Clostridium tetani	Tetanus toxin precursor	I, II
48537	Clostridium tetani	Tetanus toxin precursor	I, II
49509	Clostridium tetani	Tetanus toxin precursor	I, II
50625	Clostridium tetani	Tetanus toxin precursor	I, II
50626	Clostridium tetani	Tetanus toxin precursor	I, II
52924	Clostridium tetani	Tetanus toxin precursor	I, II
52929	Clostridium tetani	Tetanus toxin precursor	I, II
52930	Clostridium tetani	Tetanus toxin precursor	I, II
53363	Clostridium tetani	Tetanus toxin precursor	I, II
53641	Clostridium tetani	Tetanus toxin precursor	I, II
56304	Clostridium tetani	Tetanus toxin precursor	I, II
56991	Clostridium tetani	Tetanus toxin precursor	I, II
57891	Clostridium tetani	Tetanus toxin precursor	I, II
57935	Clostridium tetani	Tetanus toxin precursor	I, II
57947	Clostridium tetani	Tetanus toxin precursor	I, II
58527	Clostridium tetani	Tetanus toxin precursor	I, II
59500	Clostridium tetani	Tetanus toxin precursor	I, II
59724	Clostridium tetani	Tetanus toxin precursor	I, II
60899	Clostridium tetani	Tetanus toxin precursor	I, II
61214	Clostridium tetani	Tetanus toxin precursor	I, II
61407	Clostridium tetani	Tetanus toxin precursor	I, II
61681	Clostridium tetani	Tetanus toxin precursor	I, II
62073	Clostridium tetani	Tetanus toxin precursor	I, II
62545	Clostridium tetani	Tetanus toxin precursor	I, II
62689	Clostridium tetani	Tetanus toxin precursor	I, II
63277	Clostridium tetani	Tetanus toxin precursor	I, II
63450	Clostridium tetani	Tetanus toxin precursor	I, II
64502	Clostridium tetani	Tetanus toxin precursor	I, II
64514	Clostridium tetani	Tetanus toxin precursor	I, II
64591	Clostridium tetani	Tetanus toxin precursor	I, II
64592	Clostridium tetani	Tetanus toxin precursor	I, II
64627	Clostridium tetani	Tetanus toxin precursor	I, II
65324	Clostridium tetani	Tetanus toxin precursor	I, II
67148	Clostridium tetani	Tetanus toxin precursor	I, II
67473	Clostridium tetani	Tetanus toxin precursor	I, II
68104	Clostridium tetani	Tetanus toxin precursor	I, II
69136	Clostridium tetani	Tetanus toxin precursor	I, II
69149	Clostridium tetani	Tetanus toxin precursor	I, II
69180	Clostridium tetani	Tetanus toxin precursor	I, II
69258	Clostridium tetani	Tetanus toxin precursor	I, II
70165	Clostridium tetani	Tetanus toxin precursor	I, II
70202	Clostridium tetani	Tetanus toxin precursor	I, II
70514	Clostridium tetani	Tetanus toxin precursor	I, II
70697	Clostridium tetani	Tetanus toxin precursor	I, II
70698	Clostridium tetani	Tetanus toxin precursor	I, II
70699	Clostridium tetani	Tetanus toxin precursor	I, II
70982	Clostridium tetani	Tetanus toxin precursor	I, II
71031	Clostridium tetani	Tetanus toxin precursor	I, II
71154	Clostridium tetani	Tetanus toxin precursor	I, II
71155	Clostridium tetani	Tetanus toxin precursor	I, II
73592	Clostridium tetani	Tetanus toxin precursor	I, II
74260	Clostridium tetani	Tetanus toxin precursor	I, II
74904	Clostridium tetani	Tetanus toxin precursor	I, II
75156	Clostridium tetani	Tetanus toxin precursor	I, II
75794	Clostridium tetani	Tetanus toxin precursor	I, II

75930	Clostridium tetani	Tetanus toxin precursor	I, II
76411	Clostridium tetani	Tetanus toxin precursor	I, II
76537	Clostridium tetani	Tetanus toxin precursor	I, II
113407	Clostridium tetani	Tetanus toxin precursor	I, II
127968	Clostridium tetani	Tetanus toxin precursor	I, II
128003	Clostridium tetani	Tetanus toxin precursor	I, II
128013	Clostridium tetani	Tetanus toxin precursor	I, II
128081	Clostridium tetani	Tetanus toxin precursor	I, II
79786	Clostridium tetani E88	tetanus toxin tetX	I, II
79793	Clostridium tetani E88	tetanus toxin tetX	I, II
79808	Clostridium tetani E88	tetanus toxin tetX	I, II
79816	Clostridium tetani E88	tetanus toxin tetX	I, II
79870	Clostridium tetani E88	tetanus toxin tetX	I, II
79876	Clostridium tetani E88	tetanus toxin tetX	I, II
79883	Clostridium tetani E88	tetanus toxin tetX	I, II
79915	Clostridium tetani E88	tetanus toxin tetX	I, II
79941	Clostridium tetani E88	tetanus toxin tetX	I, II
79972	Clostridium tetani E88	tetanus toxin tetX	I, II
79984	Clostridium tetani E88	tetanus toxin tetX	I, II
79985	Clostridium tetani E88	tetanus toxin tetX	I, II
79996	Clostridium tetani E88	tetanus toxin tetX	I, II
80030	Clostridium tetani E88	tetanus toxin tetX	I, II
80048	Clostridium tetani E88	tetanus toxin tetX	I, II
80054	Clostridium tetani E88	tetanus toxin tetX	I, II
2213	Streptococcus pyogenes	M protein	I, II
2222	Streptococcus pyogenes	M protein, serotype 12 precursor	I, II
2506	Streptococcus pyogenes	M protein, serotype 5 precursor	I, II
3420	Streptococcus pyogenes	M protein	I, II
4740	Streptococcus pyogenes	M protein	I, II
8930	Streptococcus pyogenes	M5	I, II
12645	Streptococcus pyogenes	M protein, serotype 12 precursor	I, II
12945	Streptococcus pyogenes	M protein	I, II
13865	Streptococcus pyogenes	M protein, serotype 12 precursor	I, II
14426	Streptococcus pyogenes	M protein	I, II
22026	Streptococcus pyogenes	M protein, serotype 12 precursor	I, II
29866	Streptococcus pyogenes	M protein	I, II
30176	Streptococcus pyogenes	M protein, serotype 12 precursor	I, II
31797	Streptococcus pyogenes	M protein, serotype 12 precursor	I, II
31798	Streptococcus pyogenes	Plasminogen-binding group A steptococcal M-like protein PAM precursor	I, II
33019	Streptococcus pyogenes	M protein, serotype 12 precursor	I, II
33057	Streptococcus pyogenes	M5	I, II
33459	Streptococcus pyogenes	M protein, serotype 12 precursor	I, II
35679	Streptococcus pyogenes	M protein	I, II
39183	Streptococcus pyogenes	M protein	I, II
44894	Streptococcus pyogenes	Plasminogen-binding group A steptococcal M-like protein PAM precursor	I, II
45666	Streptococcus pyogenes	M protein, serotype 12 precursor	I, II
52184	Streptococcus pyogenes	M protein, serotype 5 precursor	I, II
60642	Streptococcus pyogenes	M protein, serotype 12 precursor	I, II
61850	Streptococcus pyogenes	M5 protein	I, II
63417	Streptococcus pyogenes	Plasminogen-binding group A steptococcal M-like protein PAM precursor	I, II
66083	Streptococcus pyogenes	M protein	I, II
67183	Streptococcus pyogenes	M protein	I, II
122211	Streptococcus pyogenes	M5	I, II
129727	Streptococcus pyogenes	PTS system, lactose-specific IIBC component	I, II
131873	Streptococcus pyogenes	M protein	I, II
131948	Streptococcus pyogenes	M5	I, II
132974	Streptococcus pyogenes	M6 protein - Streptococcus pyogenes	I, II
132986	Streptococcus pyogenes	M6 protein - Streptococcus pyogenes	I, II
132989	Streptococcus pyogenes	M6 protein - Streptococcus pyogenes	I, II
132992	Streptococcus pyogenes	M6 protein - Streptococcus pyogenes	I, II
133042	Streptococcus pyogenes	M6 protein - Streptococcus pyogenes	I, II
133060	Streptococcus pyogenes	M6 protein - Streptococcus pyogenes	I, II
133111	Streptococcus pyogenes	M6 protein - Streptococcus pyogenes	I, II
133162	Streptococcus pyogenes	M6 protein - Streptococcus pyogenes	I, II
133376	Streptococcus pyogenes	M6 protein - Streptococcus pyogenes	I, II
133381	Streptococcus pyogenes	M6 protein - Streptococcus pyogenes	I, II
134497	Streptococcus pyogenes	M protein	I, II
135552	Streptococcus pyogenes	RopA	I, II
135605	Streptococcus pyogenes	ScnM	I, II
142116	Streptococcus pyogenes	M protein	I, II
142125	Streptococcus pyogenes	M protein, serotype 5 precursor	I, II
142166	Streptococcus pyogenes	M protein, serotype 5 precursor	I, II
142167	Streptococcus pyogenes	M protein	I, II

142200	Streptococcus pyogenes	M protein	I, II
142201	Streptococcus pyogenes	M protein	I, II
142202	Streptococcus pyogenes	M protein	I, II
142204	Streptococcus pyogenes	M protein	I, II
142217	Streptococcus pyogenes	M protein, serotype 5 precursor	I, II
142226	Streptococcus pyogenes	M protein, serotype 5 precursor	I, II
142282	Streptococcus pyogenes	M protein, serotype 5 precursor	I, II
142288	Streptococcus pyogenes	M protein	I, II
67779	Streptococcus pyogenes serotype M24	M protein, serotype 24 precursor	I, II
2220	Streptococcus pyogenes serotype M5	M protein, serotype 5 precursor	I, II
3710	Streptococcus pyogenes serotype M5	M protein, serotype 5 precursor	I, II
5586	Streptococcus pyogenes serotype M5	M protein	I, II
8920	Streptococcus pyogenes serotype M5	M protein, serotype 5 precursor	I, II
8931	Streptococcus pyogenes serotype M5	M protein, serotype 5 precursor	I, II
11150	Streptococcus pyogenes serotype M5	M protein, serotype 5 precursor	I, II
13866	Streptococcus pyogenes serotype M5	M protein, serotype 5 precursor	I, II
21453	Streptococcus pyogenes serotype M5	M protein, serotype 5 precursor	I, II
26328	Streptococcus pyogenes serotype M5	M protein, serotype 5 precursor	I, II
31004	Streptococcus pyogenes serotype M5	M protein, serotype 5 precursor	I, II
32047	Streptococcus pyogenes serotype M5	M protein, serotype 5 precursor	I, II
32048	Streptococcus pyogenes serotype M5	M protein, serotype 5 precursor	I, II
32190	Streptococcus pyogenes serotype M5	M protein, serotype 5 precursor	I, II
36841	Streptococcus pyogenes serotype M5	M protein, serotype 5 precursor	I, II
41143	Streptococcus pyogenes serotype M5	M protein, serotype 5 precursor	I, II
44463	Streptococcus pyogenes serotype M5	M protein, serotype 5 precursor	I, II
47492	Streptococcus pyogenes serotype M5	M protein, serotype 5 precursor	I, II
50296	Streptococcus pyogenes serotype M5	M protein, serotype 5 precursor	I, II
51486	Streptococcus pyogenes serotype M5	M protein, serotype 5 precursor	I, II
57290	Streptococcus pyogenes serotype M5	M protein, serotype 5 precursor	I, II
64312	Streptococcus pyogenes serotype M5	M protein, serotype 5 precursor	I, II
66084	Streptococcus pyogenes serotype M5	M protein, serotype 5 precursor	I, II
69186	Streptococcus pyogenes serotype M5	M protein, serotype 5 precursor	I, II
122615	Streptococcus pyogenes serotype M5	M protein	I, II
56291	Streptococcus pyogenes serotype M6	M protein	I, II
10411	Vibrio cholerae	toxin	I, II
28896	Vibrio cholerae	toxin	I, II
194	Mycobacterium tuberculosis	Hypothetical protein esxG	I
327	Mycobacterium tuberculosis	10 kDa culture filtrate antigen EsxB	I
447	Mycobacterium tuberculosis H37Rv	Antigen 85-A precursor	I
1079	Mycobacterium tuberculosis	ESAT-6-like protein esxB	I
1080	Mycobacterium tuberculosis	ESAT-6-like protein esxB	I
1857	Mycobacterium tuberculosis	mycocerosic acid synthase	I
2058	Mycobacterium tuberculosis	6 kDa early secretory antigenic target	I
2135	Mycobacterium tuberculosis	Antigen 85-A precursor	I
2490	Mycobacterium tuberculosis	CYTOTOXIN|HAEMOLYSIN HOMOLOGUE TLYA	I
2556	Mycobacterium tuberculosis	PE_PGRS 33	I
2733	Mycobacterium tuberculosis	Enoyl-	I
2968	Mycobacterium tuberculosis	PstA-1	I
2969	Mycobacterium tuberculosis	PstA-1	I
3030	Mycobacterium tuberculosis	Antigen 85-A precursor	I
3060	Mycobacterium tuberculosis	thymidylate synthase	I
3064	Mycobacterium tuberculosis	6 kDa early secretory antigenic target	I
3094	Mycobacterium tuberculosis	Antigen 85-B precursor	I
3097	Mycobacterium tuberculosis H37Rv	Antigen 85-A precursor	I
3118	Mycobacterium tuberculosis	Antigen 85-C precursor	I
3153	Mycobacterium tuberculosis	60 kDa chaperonin 2	I
3167	Mycobacterium avium	Enoyl-	I
3189	Mycobacterium tuberculosis H37Rv	Antigen 85-A precursor	I
3935	Mycobacterium tuberculosis	10 kDa culture filtrate antigen EsxB	I
4002	Mycobacterium tuberculosis H37Rv	PPE family protein	I
4682	Mycobacterium tuberculosis	LOW MOLECULAR WEIGHT T-CELL ANTIGEN TB8.4	I
4897	Mycobacterium tuberculosis	6 kDa early secretory antigenic target	I
5196	Mycobacterium tuberculosis	60 kDa chaperonin 2	I
5220	Mycobacterium tuberculosis	14 kDa antigen	I
5251	Mycobacterium tuberculosis	Lipoprotein lpqH precursor	I
5361	Mycobacterium tuberculosis	alanine dehydrogenase	I
5381	Mycobacterium tuberculosis	LOW MOLECULAR WEIGHT T-CELL ANTIGEN TB8.4	I
5623	Mycobacterium tuberculosis	Antigen 85-B precursor	I
6901	Mycobacterium tuberculosis	Antigen 85-B precursor	I
8685	Mycobacterium tuberculosis	Antigen 85-B precursor	I
9099	Mycobacterium tuberculosis H37Rv	Antigen 85-A precursor	I
9414	Mycobacterium tuberculosis	Superoxide dismutase	I
9415	Mycobacterium tuberculosis	Superoxide dismutase	I

9416	Mycobacterium tuberculosis H37Rv	Antigen 85-A precursor	I
9920	Mycobacterium tuberculosis	ESAT-6-like protein esxB	I
10133	Mycobacterium tuberculosis H37Rv	Antigen 85-A precursor	I
12062	Mycobacterium tuberculosis	hypothetical glycine-rich protein Rv381	I
12583	Mycobacterium tuberculosis H37Rv	ESAT-6-like protein esxB	I
12585	Mycobacterium tuberculosis H37Rv	10 kDa culture filtrate antigen EsxB	I
13215	Mycobacterium tuberculosis	Antigen 85-B	I
13381	Mycobacterium tuberculosis	14 kDa antigen	I
13386	Mycobacterium tuberculosis	ESAT-6-like protein esxB	I
13473	Mycobacterium tuberculosis	Antigen 85-B precursor	I
13787	Mycobacterium tuberculosis	Probable signal peptidase I	I
14372	Mycobacterium tuberculosis H37Rv	Antigen 85-A precursor	I
15768	Mycobacterium tuberculosis H37Rv	Antigen 85-A precursor	I
16332	Mycobacterium tuberculosis	Antigen 85-B precursor	I
16333	Mycobacterium tuberculosis	Antigen 85-B precursor	I
16570	Mycobacterium tuberculosis H37Rv	Antigen 85-A precursor	I
16705	Mycobacterium tuberculosis	Acetyl-/propionyl-coenzyme A carboxylase alpha chain	I
16739	Mycobacterium tuberculosis	Phosphate-binding protein pstS 1 precursor	I
16773	Mycobacterium tuberculosis	Elongation factor Tu	I
16910	Mycobacterium tuberculosis	mycocerosic acid synthase	I
16924	Mycobacterium tuberculosis	Antigen 85-B precursor	I
17099	Mycobacterium tuberculosis	Putative virulence-regulating 38 kDa protein	I
17728	Mycobacterium tuberculosis H37Rv	Antigen 85-A precursor	I
18276	Mycobacterium tuberculosis	Antigen 85-B precursor	I
18312	Mycobacterium tuberculosis	PstA-1	I
18464	Mycobacterium tuberculosis H37Rv	Antigen 85-A precursor	I
18550	Mycobacterium tuberculosis	14 kDa antigen	I
18744	Mycobacterium tuberculosis H37Rv	Antigen 85-A precursor	I
18811	Mycobacterium tuberculosis H37Rv	Antigen 85-A precursor	I
19348	Mycobacterium tuberculosis	IMMUNOGENIC PROTEIN MPT64 (ANTIGEN MPT64/MPB64)	I
19359	Mycobacterium tuberculosis	IMMUNOGENIC PROTEIN MPT64 (ANTIGEN MPT64/MPB64)	I
19878	Mycobacterium tuberculosis H37Rv	Antigen 85-A precursor	I
20369	Mycobacterium tuberculosis	14 kDa antigen	I
20475	Mycobacterium tuberculosis	PROBABLE SERINE PROTEASE PEPA (MTB32A)	I
20481	Mycobacterium tuberculosis	PstA-1	I
20503	Mycobacterium tuberculosis H37Rv	Antigen 85-A precursor	I
20615	Mycobacterium tuberculosis H37Rv	Antigen 85-A precursor	I
20761	Mycobacterium tuberculosis	3-phosphoshikimate 1-carboxyvinyltransferase	I
20859	Mycobacterium tuberculosis	60 kDa chaperonin 2	I
20860	Mycobacterium tuberculosis	60 kDa chaperonin 2	I
20883	Mycobacterium tuberculosis	mycocerosic acid synthase	I
20910	Mycobacterium tuberculosis H37Ra	ISONIAZID INDUCTIBLE GENE PROTEIN INIB	I
20911	Mycobacterium tuberculosis H37Ra	ISONIAZID INDUCTIBLE GENE PROTEIN INIB	I
20912	Mycobacterium tuberculosis H37Ra	ISONIAZID INDUCTIBLE GENE PROTEIN INIB	I
21000	Mycobacterium tuberculosis	Glutamine synthetase 1	I
21040	Mycobacterium tuberculosis	PstA-1	I
21078	Mycobacterium tuberculosis	Antigen 85-A precursor	I
21153	Mycobacterium tuberculosis	Alanine dehydrogenase	I
21185	Mycobacterium tuberculosis	DNA gyrase subunit B	I
21222	Mycobacterium tuberculosis	diaminopimelate decarboxylase	I
21231	Mycobacterium tuberculosis	Putative virulence-regulating 38 kDa protein	I
21240	Mycobacterium tuberculosis	Acetyl-/propionyl-coenzyme A carboxylase alpha chain	I
21243	Mycobacterium tuberculosis	DNA-directed RNA polymerase subunit beta'	I
21275	Mycobacterium tuberculosis	Antigen 85-B precursor	I
21780	Mycobacterium tuberculosis	Antigen 85-B precursor	I
21960	Mycobacterium tuberculosis	Antigen 85-B precursor	I
21976	Mycobacterium tuberculosis	10 kDa culture filtrate antigen EsxB	I
22633	Mycobacterium tuberculosis	Immunogenic protein MPT64 precursor	I
23306	Mycobacterium tuberculosis	ESAT-6-like protein esxH	I
26791	Mycobacterium tuberculosis H37Rv	Antigen 85-A precursor	I
27053	Mycobacterium tuberculosis	alanine dehydrogenase	I
27191	Mycobacterium tuberculosis	PstA-1	I
27363	Mycobacterium tuberculosis	unnamed protein product	I
27447	Mycobacterium tuberculosis str. Haarlem	PPE family protein	I
27586	Mycobacterium sp. KMS	Hypothetical protein	I
27786	Mycobacterium tuberculosis H37Rv	Antigen 85-B precursor	I
27900	Mycobacterium tuberculosis H37Rv	PROBABLE TRANSPOSASE FOR INSERTION SEQUENCE ELEMENT IS1081	I
27901	Mycobacterium tuberculosis	Antigen 85-B precursor	I
27981	Mycobacterium tuberculosis	Immunogenic protein MPT64 precursor	I
28040	Mycobacterium tuberculosis H37Rv	hypothetical protein Rv1461	I
28364	Mycobacterium tuberculosis	ESAT-6-like protein esxB	I
28506	Mycobacterium tuberculosis	10 kDa culture filtrate antigen EsxB	I
28594	Mycobacterium tuberculosis	Immunogenic protein MPT64 precursor	I

29558	Mycobacterium tuberculosis	Antigen 85-B precursor	I
31250	Mycobacterium tuberculosis	Immunogenic protein MPT64 precursor	I
31749	Mycobacterium tuberculosis	60 KDA chaperonin 2 groel2	I
31902	Mycobacterium tuberculosis	Antigen 85-A precursor	I
32103	Mycobacterium tuberculosis	60 kDa chaperonin (Protein Cpn60) (groEL protein)	I
32186	Mycobacterium tuberculosis	mycocerosic acid synthase	I
32213	Mycobacterium tuberculosis	Antigen 85-B precursor	I
32710	Mycobacterium tuberculosis H37Rv	hypothetical protein Rv3467	I
32860	Mycobacterium tuberculosis H37Rv	endonuclease IV	I
34866	Mycobacterium tuberculosis	PstA-1	I
34928	Mycobacterium tuberculosis	10 kDa culture filtrate antigen EsxB	I
35785	Mycobacterium tuberculosis	14 kDa antigen	I
37069	Mycobacterium tuberculosis	60 kDa chaperonin 2	I
37075	Mycobacterium tuberculosis	ISMt1 transposase B	I
37084	Mycobacterium tuberculosis	Glutamine synthetase 1	I
37109	Mycobacterium tuberculosis	mycocerosic acid synthase	I
37112	Mycobacterium tuberculosis	Phosphate-binding protein pstS 1 precursor	I
37118	Mycobacterium tuberculosis	Immunogenic protein MPT64 precursor	I
37140	Mycobacterium tuberculosis	Hypothetical protein esxG	I
37146	Mycobacterium tuberculosis	6 kDa early secretory antigenic target	I
37155	Mycobacterium tuberculosis H37Rv	Antigen 85-A precursor	I
37272	Mycobacterium tuberculosis	tuberculin-like peptide	I
37296	Mycobacterium tuberculosis	DNA gyrase subunit B	I
37297	Mycobacterium tuberculosis	DNA gyrase subunit B	I
37365	Mycobacterium tuberculosis	DnaK	I
37476	Mycobacterium tuberculosis	DnaK	I
37483	Mycobacterium tuberculosis	mycocerosic acid synthase	I
37667	Mycobacterium bovis	DNA-directed RNA polymerase subunit beta'	I
37785	Mycobacterium tuberculosis	Alanine dehydrogenase	I
37835	Mycobacterium tuberculosis	PROBABLE SERINE PROTEASE PEPA (MTB32A)	I
37951	Mycobacterium tuberculosis	Peroxidase/catalase T	I
38049	Mycobacterium tuberculosis	Antigen 85-B precursor	I
38050	Mycobacterium tuberculosis	Antigen 85-B precursor	I
38071	Mycobacterium tuberculosis	DNA-binding response regulator mtrA	I
38515	Mycobacterium tuberculosis H37Rv	Phosphate-binding protein pstS 1 precursor	I
38969	Mycobacterium tuberculosis H37Rv	Antigen 85-A precursor	I
40361	Mycobacterium tuberculosis H37Rv	Antigen 85-A precursor	I
40622	Mycobacterium tuberculosis	CYTOTOXIN|HAEMOLYSIN HOMOLOGUE TLYA	I
41030	Mycobacterium tuberculosis	10 KDA CULTURE FILTRATE ANTIGEN ESXB (LHP) (CFP10)	I
41078	Mycobacterium tuberculosis	thymidylate synthase	I
41558	Mycobacterium tuberculosis H37Rv	Antigen 85-A precursor	I
41618	Mycobacterium tuberculosis H37Rv	Antigen 85-A precursor	I
41910	Mycobacterium tuberculosis	mycocerosic acid synthase	I
41911	Mycobacterium tuberculosis	Peroxidase/catalase T	I
41947	Mycobacterium tuberculosis	mtp40 protein - Mycobacterium tuberculosis	I
42038	Mycobacterium tuberculosis	60 kDa chaperonin 2	I
42342	Mycobacterium tuberculosis	Antigen 85-B precursor	I
42393	Mycobacterium tuberculosis H37Rv	Antigen 85-A precursor	I
42897	Mycobacterium bovis	DNA-directed RNA polymerase subunit beta	I
43447	Mycobacterium tuberculosis	Immunogenic protein MPT64 precursor	I
44327	Mycobacterium tuberculosis	ESAT-6-like protein esxB	I
44802	Mycobacterium tuberculosis	hypothetical glycine-rich protein Rv3812	I
46499	Mycobacterium tuberculosis	6 kDa early secretory antigenic target	I
46625	Mycobacterium tuberculosis	hypothetical glycine-rich protein Rv3812	I
48458	Mycobacterium tuberculosis	Immunogenic protein MPT64 precursor	I
48580	Mycobacterium bovis	PPE FAMILY PROTEIN	I
51104	Mycobacterium tuberculosis H37Rv	LOW MOLECULAR WEIGHT PROTEIN ANTIGEN 7 ESXH	I
51535	Mycobacterium tuberculosis	Glutamine synthetase 1	I
51643	Mycobacterium tuberculosis	Immunogenic protein MPT64 precursor	I
52431	Mycobacterium tuberculosis	Antigen 85-B precursor	I
52690	Mycobacterium tuberculosis	10 kDa culture filtrate antigen EsxB	I
53077	Mycobacterium tuberculosis H37Rv	ESAT-6-like protein esxB	I
53370	Mycobacterium tuberculosis	IMMUNOGENIC PROTEIN MPT64 (ANTIGEN MPT64/MPB64)	I
54122	Mycobacterium tuberculosis	alanine dehydrogenase	I
54635	Mycobacterium tuberculosis	Tuberculin-active protein	I
54671	Mycobacterium tuberculosis	Antigen 85-B precursor	I
54704	Mycobacterium tuberculosis	Uncharacterized protein Rv2715/MT2788	I
54722	Mycobacterium tuberculosis	Peroxidase/catalase T	I
54812	Mycobacterium tuberculosis	secreted antigen Ag85A	I
54878	Mycobacterium tuberculosis	thymidylate synthase	I
54950	Mycobacterium tuberculosis	PstA-1	I
55156	Mycobacterium tuberculosis H37Rv	hypothetical protein Rv1945	I
55188	Mycobacterium tuberculosis H37Rv	translation initiation factor IF-3	I

55191	Mycobacterium tuberculosis H37Rv	PROBABLE CONSERVED TRANSMEMBRANE PROTEIN	I
55192	Mycobacterium tuberculosis	Hypothetical protein	I
55199	Mycobacterium tuberculosis H37Rv	1-acylglycerol-3-phosphate O-acyltransferase	I
55315	Mycobacterium tuberculosis CDC1551	CRISPR-associated protein, TM1811 family	I
55324	Mycobacterium tuberculosis H37Rv	PROBABLE PERIPLASMIC OLIGOPEPTIDE-BINDING LIPOPROTEIN OPPA	I
55334	Mycobacterium tuberculosis H37Rv	PROBABLE NAD-DEPENDENT GLUTAMATE DEHYDROGENASE GDH	I
58045	Mycobacterium tuberculosis H37Rv	Antigen 85-A precursor	I
58994	Mycobacterium tuberculosis	Putative virulence-regulating 38 kDa protein	I
59099	Mycobacterium tuberculosis	14 kDa antigen	I
59250	Mycobacterium tuberculosis	DnaK	I
59251	Mycobacterium tuberculosis	DnaK	I
59594	Mycobacterium tuberculosis	PstA-1	I
59595	Mycobacterium tuberculosis	PstA-1	I
59627	Mycobacterium tuberculosis	Antigen 85-B precursor	I
60095	Mycobacterium tuberculosis H37Rv	hypothetical protein Rv3714c	I
62797	Mycobacterium tuberculosis	ESAT-6-like protein esxB	I
63957	Mycobacterium tuberculosis H37Rv	Antigen 85-A precursor	I
64412	Mycobacterium tuberculosis	PE_PGRS 33	I
64714	Mycobacterium tuberculosis	MPT51/MPB51 antigen precursor	I
64949	Mycobacterium tuberculosis	Phosphate-binding protein pstS 1 precursor	I
65965	Mycobacterium tuberculosis	Immunogenic protein MPT64 precursor	I
67077	Mycobacterium tuberculosis	3-phosphoshikimate 1-carboxyvinyltransferase	I
68413	Mycobacterium tuberculosis H37Rv	Antigen 85-A precursor	I
69514	Mycobacterium tuberculosis	Alanine and proline-rich secreted protein apa precursor	I
69532	Mycobacterium tuberculosis	mycocerosic acid synthase	I
69584	Mycobacterium tuberculosis	DNA gyrase subunit B	I
69608	Mycobacterium tuberculosis	DnaK	I
69646	Mycobacterium tuberculosis	Alanine dehydrogenase	I
69798	Mycobacterium tuberculosis	Lipoprotein lpqH precursor	I
70507	Mycobacterium tuberculosis H37Rv	Soluble secreted antigen MPT53 precursor	I
70687	Mycobacterium tuberculosis	10 kDa culture filtrate antigen EsxB	I
71338	Mycobacterium tuberculosis H37Rv	Antigen 85-A precursor	I
71765	Mycobacterium tuberculosis H37Rv	Antigen 85-A precursor	I
72750	Mycobacterium tuberculosis	mycocerosic acid synthase	I
72756	Mycobacterium tuberculosis	PROBABLE SERINE PROTEASE PEPA (MTB32A)	I
72770	Mycobacterium tuberculosis H37Rv	Antigen 85-A precursor	I
72837	Mycobacterium tuberculosis	DNA-directed RNA polymerase subunit beta'	I
72965	Mycobacterium tuberculosis H37Rv	Antigen 85-C precursor	I
72985	Mycobacterium tuberculosis H37Rv	LOW MOLECULAR WEIGHT PROTEIN ANTIGEN 7 ESXH	I
73003	Mycobacterium tuberculosis H37Rv	Antigen 85-A precursor	I
73234	Mycobacterium tuberculosis H37Rv	Antigen 85-A precursor	I
73306	Mycobacterium tuberculosis	Antigen 85-B precursor	I
74130	Mycobacterium tuberculosis H37Rv	Antigen 85-A precursor	I
74606	Mycobacterium tuberculosis	Protein not available	I
74761	Mycobacterium tuberculosis	3-dehydroquinate dehydratase	I
74768	Mycobacterium tuberculosis	Antigen 85-B precursor	I
74914	Mycobacterium tuberculosis	Phosphate-binding protein pstS 1 precursor	I
74927	Mycobacterium tuberculosis	Putative virulence-regulating 38 kDa protein	I
75030	Mycobacterium tuberculosis	Putative virulence-regulating 38 kDa protein	I
76221	Mycobacterium tuberculosis	Antigen 85-C precursor	I
87675	Mycobacterium tuberculosis	6 kDa early secretory antigenic target	I
92301	Mycobacterium bovis BCG str. Pasteur 1173P2	ATP-dependent Clp protease proteolytic subunit	I
92817	Mycobacterium tuberculosis	Secreted antigen Ag85B	I
93270	Mycobacterium tuberculosis	Hypothetical protein	I
108957	Mycobacterium tuberculosis	hypothetical protein	I
108965	Mycobacterium tuberculosis	Prolipoprotein diacylglyceryl transferase	I
124973	Mycobacterium tuberculosis H37Rv	Esat-6 like protein esxJ (Esat-6 like protein 2)	I
126028	Mycobacterium tuberculosis CDC1551	ATP-dependent helicase	I
136142	Mycobacterium tuberculosis H37Rv	PROBABLE CUTINASE PRECURSOR CFP21	I
137437	Mycobacterium tuberculosis	Esat-6 like protein esxJ (Esat-6 like protein 2)	I
138865	Mycobacterium tuberculosis	D-alanyl-D-alanine carboxypeptidase	I
138896	Mycobacterium tuberculosis	Hypothetical protein	I
140541	Mycobacterium tuberculosis H37Rv	hypothetical protein	I
140542	Mycobacterium tuberculosis H37Rv	MCE-family protein MCE4B	I
140543	Mycobacterium tuberculosis	Uncharacterized glycosyl hydrolase Rv2006/MT2062	I
140561	Mycobacterium tuberculosis H37Rv	metal cation transporter P-type ATPase A CtpF	I
140564	Mycobacterium tuberculosis H37Rv	esterase lipoprotein LpqC	I
140576	Mycobacterium tuberculosis H37Rv	secreted L-alanine dehydrogenase ALD (40 kDa antigen) (TB43)	I
140597	Mycobacterium tuberculosis H37Rv	MCE-family protein MCE4A	I
140599	Mycobacterium tuberculosis H37Rv	MCE-family lipoprotein LprL	I
140600	Mycobacterium tuberculosis H37Rv	glutamine synthetase GLNA1 (glutamine synthase) (GS-I)	I
140615	Mycobacterium tuberculosis H37Rv	amino acid decarboxylase	I
140616	Mycobacterium tuberculosis H37Rv	membrane-associated phospholipase C	I

140617	Mycobacterium tuberculosis H37Rv	hypothetical protein	I
140623	Mycobacterium tuberculosis H37Rv	MCE-family protein MCE3A	I
140649	Mycobacterium tuberculosis H37Rv	LOW MOLECULAR WEIGHT PROTEIN ANTIGEN 7 ESXH	I
140652	Mycobacterium tuberculosis H37Rv	LOW MOLECULAR WEIGHT PROTEIN ANTIGEN 7 ESXH	I
140654	Mycobacterium tuberculosis H37Rv	LOW MOLECULAR WEIGHT PROTEIN ANTIGEN 7 ESXH	I
140706	Mycobacterium tuberculosis H37Rv	LOW MOLECULAR WEIGHT PROTEIN ANTIGEN 7 ESXH	I
140708	Mycobacterium tuberculosis H37Rv	LOW MOLECULAR WEIGHT PROTEIN ANTIGEN 7 ESXH	I
140712	Mycobacterium tuberculosis H37Rv	LOW MOLECULAR WEIGHT PROTEIN ANTIGEN 7 ESXH	I
140727	Mycobacterium tuberculosis H37Rv	LOW MOLECULAR WEIGHT PROTEIN ANTIGEN 7 ESXH	I
140744	Mycobacterium tuberculosis H37Rv	LOW MOLECULAR WEIGHT PROTEIN ANTIGEN 7 ESXH	I
140760	Mycobacterium tuberculosis H37Rv	LOW MOLECULAR WEIGHT PROTEIN ANTIGEN 7 ESXH	I
140769	Mycobacterium tuberculosis H37Rv	LOW MOLECULAR WEIGHT PROTEIN ANTIGEN 7 ESXH	I
141202	Mycobacterium tuberculosis H37Rv	Uncharacterized PPE family protein PPE51	I
142231	Mycobacterium tuberculosis	Lipoprotein lpqH precursor	I
142232	Mycobacterium tuberculosis	POSSIBLE CONSERVED TRANSMEMBRANE PROTEIN	I
142233	Mycobacterium tuberculosis	ATP-dependent helicase	I
144867	Mycobacterium tuberculosis	Putative transposase Rv3428c	I
144868	Mycobacterium tuberculosis	Uncharacterized glycosyl hydrolase Rv2006/MT2062	I
144869	Mycobacterium tuberculosis	Uncharacterized protein Rv2074/MT2134	I
144876	Mycobacterium tuberculosis	Putative diacyglycerol O-acyltransferase Rv3087/MT3172	I
144877	Mycobacterium tuberculosis	Hypothetical protein	I
144882	Mycobacterium tuberculosis	Uncharacterized protein Rv2627c/MT2702	I
144883	Mycobacterium tuberculosis	transmembrane cation transporter	I
144884	Mycobacterium tuberculosis	Uncharacterized MFS-type transporter Rv0191/MT0201	I
144886	Mycobacterium tuberculosis	PROBABLE TRANSPOSASE	I
144887	Mycobacterium tuberculosis	50S ribosomal protein L9	I
144890	Mycobacterium tuberculosis	AMP-binding family protein	I
144894	Mycobacterium tuberculosis	hypothetical protein	I
144895	Mycobacterium tuberculosis	hypothetical protein	I
144896	Mycobacterium tuberculosis	Probable nitrate/nitrite transporter narK2	I
144897	Mycobacterium tuberculosis	Pyruvate kinase	I
144905	Mycobacterium tuberculosis	Uncharacterized protein Rv2030c/MT2089	I
144908	Mycobacterium tuberculosis	MCE-FAMILY PROTEIN MCE1C	I
144909	Mycobacterium tuberculosis	Membrane transport protein mmpL8	I
144914	Mycobacterium tuberculosis	PROBABLE TRANSPOSASE	I
144915	Mycobacterium tuberculosis	Uncharacterized protein Rv2075c/MT2135	I
144916	Mycobacterium tuberculosis	CONSERVED HYPOTHETICAL INTEGRAL MEMBRANE PROTEIN YRBE3B	I
144917	Mycobacterium tuberculosis	Cell division protein ftsX homolog	I
144918	Mycobacterium tuberculosis	PROBABLE ENOYL-CoA HYDRATASE ECHA1	I
144922	Mycobacterium tuberculosis	Putative membrane protein mmpL3	I
144926	Mycobacterium tuberculosis	Putative diacyglycerol O-acyltransferase Rv0221/MT0231	I
144928	Mycobacterium tuberculosis	Cold-shock DEAD box protein A homolog	I
144929	Mycobacterium tuberculosis	ESX-1 secretion system protein EccCb1	I
144930	Mycobacterium tuberculosis	Uncharacterized protein Rv0959/MT0986	I
144935	Mycobacterium tuberculosis	Putative maltooligosyl trehalose synthase	I
144936	Mycobacterium tuberculosis	Hypothetical protein	I
144937	Mycobacterium tuberculosis	Uncharacterized protein Rv2004c/MT2060	I
144938	Mycobacterium tuberculosis	Hypothetical protein	I
144939	Mycobacterium tuberculosis	Hypothetical protein	I
144941	Mycobacterium tuberculosis	Hypothetical protein	I
144942	Mycobacterium tuberculosis	PROBABLE OXIDOREDUCTASE	I
144943	Mycobacterium tuberculosis	POSSIBLE TYPE I RESTRICTION/MODIFICATION SYSTEM SPECIFICITY DETERI	I
144944	Mycobacterium tuberculosis	Error-prone DNA polymerase	I
144945	Mycobacterium tuberculosis	PROBABLE CONSERVED TRANSMEMBRANE PROTEIN	I
144947	Mycobacterium tuberculosis	Enoyl-	I
144948	Mycobacterium tuberculosis	Putative phenylalanine aminotransferase	I
144949	Mycobacterium tuberculosis	Phthiocerol synthesis polyketide synthase type I PpsB	I
144950	Mycobacterium tuberculosis	POSSIBLE ALANINE RICH DEHYDROGENASE	I
144951	Mycobacterium tuberculosis	PROBABLE PROLINE DEHYDROGENASE	I
144953	Mycobacterium tuberculosis	Uncharacterized protein Rv0894/MT0918	I
144956	Mycobacterium tuberculosis	Bifunctional enzyme CysN/CysC	I
144957	Mycobacterium tuberculosis	Maf-like protein Rv3282/MT3381	I
144958	Mycobacterium tuberculosis	Uncharacterized protein Rv2030c/MT2089	I
144963	Mycobacterium tuberculosis	Probable membrane protein Rv1733c/MT1774	I
144966	Mycobacterium tuberculosis	POSSIBLE CONSERVED MCE ASSOCIATED MEMBRANE PROTEIN	I
144968	Mycobacterium tuberculosis	PROBABLE TRANSPOSASE	I
144969	Mycobacterium tuberculosis	Hypothetical protein	I
144970	Mycobacterium tuberculosis	Putative membrane protein mmpL3	I
144971	Mycobacterium tuberculosis	Probable methyltransferase	I
144972	Mycobacterium tuberculosis	PROBABLE LIPOPROTEIN AMINOPEPTIDASE LPQL	I
144973	Mycobacterium tuberculosis	POSSIBLE TRANSCRIPTIONAL REGULATORY PROTEIN	I
144975	Mycobacterium tuberculosis	Uncharacterized protein Rv1405c/MT1449	I
144976	Mycobacterium tuberculosis	Uncharacterized protein Rv1518/MT1568	I

144977	Mycobacterium tuberculosis	Uncharacterized protein Rv1813c/MT1861	I
144978	Mycobacterium tuberculosis	Uncharacterized protein Rv1734c/MT1774.1	I
144980	Mycobacterium tuberculosis	MCE-FAMILY PROTEIN MCE3B	I
144981	Mycobacterium tuberculosis	PROBABLE SUCCINATE DEHYDROGENASE	I
144982	Mycobacterium tuberculosis	U0002l	I
35	Mycobacterium tuberculosis	Phosphate-binding protein pstS 1 precursor	II
60	Mycobacterium tuberculosis	6 kDa early secretory antigenic target	II
93	Mycobacterium bovis BCG	secreted antigen 85-B fbpB	II
153	Mycobacterium bovis BCG	SECRETED ANTIGEN 85-B FBPB	II
183	Mycobacterium tuberculosis	PPE FAMILY PROTEIN	II
189	Mycobacterium tuberculosis H37Rv	10 KDA CULTURE FILTRATE ANTIGEN ESXB (LHP) (CFP10)	II
191	Mycobacterium tuberculosis	PPE FAMILY PROTEIN	II
222	Mycobacterium bovis BCG	secreted antigen 85-B fbpB	II
224	Mycobacterium bovis AN5	protein or antigen 85-B	II
327	Mycobacterium tuberculosis	10 kDa culture filtrate antigen EsxB	II
328	Mycobacterium tuberculosis H37Rv	ESAT-6-like protein esxB	II
433	Mycobacterium tuberculosis	19 KDA LIPOPROTEIN ANTIGEN PRECURSOR LPQH	II
439	Mycobacterium tuberculosis H37Rv	ESAT-6-like protein esxN	II
459	Mycobacterium tuberculosis	Lipoprotein lpqH precursor	II
502	Mycobacterium bovis BCG	secreted antigen 85-B fbpB	II
506	Mycobacterium tuberculosis	ESAT-6-like protein esxB	II
511	Mycobacterium tuberculosis	6 kDa early secretory antigenic target	II
528	Mycobacterium bovis BCG	secreted antigen 85-B fbpB	II
529	Mycobacterium bovis BCG	Antigen 85-B precursor	II
556	Mycobacterium bovis BCG	secreted antigen 85-B fbpB	II
726	Mycobacterium tuberculosis H37Rv	MCE-FAMILY PROTEIN MCE2A	II
904	Mycobacterium bovis BCG	secreted antigen 85-B fbpB	II
905	Mycobacterium bovis BCG	Antigen 85-B precursor	II
965	Mycobacterium tuberculosis H37Rv	PUTATIVE ESAT-6 LIKE PROTEIN ESXL (ESAT-6 LIKE PROTEIN 4)	II
966	Mycobacterium tuberculosis Erdman	antigen Mtb9.9B	II
967	Mycobacterium tuberculosis H37Rv	ESAT-6-like protein esxN	II
1197	Mycobacterium tuberculosis	Phosphate-binding protein pstS 1 precursor	II
1473	Mycobacterium tuberculosis	60 KDA CHAPERONIN 2 GROEL2	II
1525	Mycobacterium tuberculosis	60 KDA CHAPERONIN 2 GROEL2	II
1539	Mycobacterium tuberculosis	60 KDA CHAPERONIN 2 GROEL2	II
1544	Mycobacterium bovis BCG	SECRETED ANTIGEN 85-B FBPB	II
1545	Mycobacterium tuberculosis	Antigen 85-B	II
1546	Mycobacterium tuberculosis	Antigen 85-A precursor	II
1549	Mycobacterium tuberculosis	60 KDA CHAPERONIN 2 GROEL2	II
1581	Mycobacterium tuberculosis	60 kDa chaperonin 2	II
1600	Mycobacterium tuberculosis H37Rv	PUTATIVE ESAT-6 LIKE PROTEIN ESXL (ESAT-6 LIKE PROTEIN 4)	II
1601	Mycobacterium tuberculosis Erdman	antigen Mtb9.9B	II
1629	Mycobacterium tuberculosis	Lipoprotein lpqH precursor	II
1674	Mycobacterium tuberculosis	60 KDA CHAPERONIN 2 GROEL2	II
1682	Mycobacterium bovis BCG	secreted antigen 85-B fbpB	II
2007	Mycobacterium tuberculosis	60 KDA CHAPERONIN 2 GROEL2	II
2048	Mycobacterium tuberculosis	Immunogenic protein MPT64 precursor	II
2145	Mycobacterium bovis	60 KDA CHAPERONIN 2 GROEL2	II
2434	Mycobacterium tuberculosis	PPE FAMILY PROTEIN	II
2694	Mycobacterium bovis BCG	Secreted antigen Ag85B	II
2695	Mycobacterium tuberculosis	Antigen 85-B	II
2879	Mycobacterium bovis BCG	Immunogenic protein MPB70 precursor	II
2922	Mycobacterium tuberculosis	PPE FAMILY PROTEIN	II
3081	Mycobacterium tuberculosis	ESAT-6-like protein esxH	II
3196	Mycobacterium tuberculosis H37Rv	ESAT-6-like protein esxH	II
3248	Mycobacterium tuberculosis	10 kDa chaperonin	II
3399	Mycobacterium bovis BCG	SECRETED ANTIGEN 85-B FBPB	II
3400	Mycobacterium tuberculosis	Antigen 85-B	II
3403	Mycobacterium tuberculosis	Antigen 85-A precursor	II
3422	Mycobacterium tuberculosis	Antigen 85-A precursor	II
3505	Mycobacterium tuberculosis	Phosphate-binding protein pstS 1 precursor	II
3629	Mycobacterium tuberculosis	Immunogenic protein MPT64 precursor	II
3865	Mycobacterium tuberculosis	60 KDA CHAPERONIN 2 GROEL2	II
3935	Mycobacterium tuberculosis	10 kDa culture filtrate antigen EsxB	II
4132	Mycobacterium tuberculosis	10 kDa chaperonin	II
4187	Mycobacterium tuberculosis	PPE FAMILY PROTEIN	II
4334	Mycobacterium tuberculosis	Lipoprotein lpqH precursor	II
4426	Mycobacterium tuberculosis	10 kDa chaperonin	II
4556	Mycobacterium tuberculosis	mtp40 protein - Mycobacterium tuberculosis	II
4727	Mycobacterium tuberculosis	PPE FAMILY PROTEIN	II
4871	Mycobacterium bovis BCG	secreted antigen 85-B fbpB	II
4942	Mycobacterium tuberculosis	6 kDa early secretory antigenic target	II
4990	Mycobacterium tuberculosis	19 KDA LIPOPROTEIN ANTIGEN PRECURSOR LPQH	II

5063	Mycobacterium tuberculosis	PPE FAMILY PROTEIN	II
5196	Mycobacterium tuberculosis	60 kDa chaperonin 2	II
5245	Mycobacterium bovis BCG	Immunogenic protein MPB70 precursor	II
5422	Mycobacterium tuberculosis	60 kDa chaperonin 2	II
5661	Mycobacterium tuberculosis	6 kDa early secretory antigenic target	II
6322	Mycobacterium bovis BCG	secreted antigen 85-B fbpB	II
6323	Mycobacterium tuberculosis	Antigen 85-B	II
6348	Mycobacterium tuberculosis	Phosphate-binding protein pstS 1 precursor	II
7470	Mycobacterium tuberculosis	Phosphate-binding protein pstS 1 precursor	II
7472	Mycobacterium tuberculosis	Phosphate-binding protein pstS 1 precursor	II
7529	Mycobacterium tuberculosis Erdman	antigen Mtb9.9B	II
7530	Mycobacterium tuberculosis H37Rv	ESAT-6-like protein esxN	II
7531	Mycobacterium tuberculosis H37Rv	Putative ESAT-6-like protein 6	II
7703	Mycobacterium tuberculosis H37Rv	ESAT-6-like protein esxQ	II
7799	Mycobacterium tuberculosis	14 kDa antigen	II
7879	Mycobacterium tuberculosis	60 KDA CHAPERONIN 2 GROEL2	II
7893	Mycobacterium bovis BCG	secreted antigen 85-B fbpB	II
7894	Mycobacterium bovis BCG	Antigen 85-B precursor	II
8411	Mycobacterium tuberculosis	Putative lipoprotein lppX precursor	II
8464	Mycobacterium tuberculosis	Lipoprotein lpqH precursor	II
8590	Mycobacterium tuberculosis H37Rv	ESAT-6-like protein esxR	II
8591	Mycobacterium tuberculosis H37Rv	ESAT-6-like protein esxQ	II
8686	Mycobacterium tuberculosis	Antigen 85-A precursor	II
8687	Mycobacterium bovis BCG	SECRETED ANTIGEN 85-B FBPB	II
8688	Mycobacterium tuberculosis	Antigen 85-B	II
9352	Mycobacterium tuberculosis	Lipoprotein lpqH precursor	II
9474	Mycobacterium tuberculosis H37Rv	PPE FAMILY PROTEIN	II
9475	Mycobacterium tuberculosis H37Rv	PPE FAMILY PROTEIN	II
9613	Mycobacterium tuberculosis	14 kDa antigen	II
9649	Mycobacterium tuberculosis H37Rv	MCE-FAMILY PROTEIN MCE2A	II
9923	Mycobacterium tuberculosis	Phosphate-binding protein pstS 1 precursor	II
9924	Mycobacterium tuberculosis	Phosphate-binding protein pstS 1 precursor	II
10022	Mycobacterium tuberculosis H37Rv	PPE FAMILY PROTEIN	II
10043	Mycobacterium bovis	60 KDA CHAPERONIN 2 GROEL2	II
10533	Mycobacterium bovis BCG str. Pasteur 1173P2	PPE family protein	II
10571	Mycobacterium bovis	60 KDA CHAPERONIN 2 GROEL2	II
10838	Mycobacterium tuberculosis	Antigen 85-A precursor	II
10840	Mycobacterium bovis BCG	Antigen 85-B precursor	II
10842	Mycobacterium bovis	alpha-antigen	II
10984	Mycobacterium tuberculosis	6 kDa early secretory antigenic target	II
11937	Mycobacterium bovis BCG	10 kDa chaperonin	II
12151	Mycobacterium tuberculosis	10 kDa chaperonin	II
12206	Mycobacterium tuberculosis	6 kDa early secretory antigenic target	II
12207	Mycobacterium tuberculosis H37Rv	6 kDa early secretory antigenic target	II
12311	Mycobacterium tuberculosis H37Rv	MCE-FAMILY PROTEIN MCE2A	II
12366	Mycobacterium tuberculosis	Lipoprotein lpqH	II
12402	Mycobacterium tuberculosis	ESAT-6-like protein esxH	II
12583	Mycobacterium tuberculosis H37Rv	ESAT-6-like protein esxB	II
12585	Mycobacterium tuberculosis H37Rv	10 kDa culture filtrate antigen EsxB	II
13836	Mycobacterium tuberculosis	Putative lipoprotein lppX precursor	II
13839	Mycobacterium tuberculosis	60 KDA CHAPERONIN 2 GROEL2	II
14574	Mycobacterium tuberculosis	10 kDa chaperonin	II
15116	Mycobacterium tuberculosis	Antigen 85-B precursor	II
15177	Mycobacterium tuberculosis	6 kDa early secretory antigenic target	II
15309	Mycobacterium tuberculosis	HEAT SHOCK PROTEIN HSPX	II
15812	Mycobacterium tuberculosis	PPE FAMILY PROTEIN	II
16334	Mycobacterium bovis BCG	alpha-antigen	II
16926	Mycobacterium bovis BCG	Antigen 85-B precursor	II
17199	Mycobacterium tuberculosis	Phosphate-binding protein pstS 1 precursor	II
17348	Mycobacterium bovis BCG	secreted antigen 85-B fbpB	II
17532	Mycobacterium bovis BCG	secreted antigen 85-B fbpB	II
17759	Mycobacterium bovis BCG	Immunogenic protein MPB70 precursor	II
17833	Mycobacterium bovis BCG	secreted antigen 85-B fbpB	II
17836	Mycobacterium bovis BCG	Antigen 85-B precursor	II
17838	Mycobacterium tuberculosis	Antigen 85-A precursor	II
18344	Mycobacterium tuberculosis Erdman	ESAT-6-like protein esxI	II
18345	Mycobacterium tuberculosis H37Rv	ESAT-6-like protein esxN	II
18509	Mycobacterium tuberculosis	Lipoprotein lpqH precursor	II
18526	Mycobacterium tuberculosis	Lipoprotein lpqH precursor	II
18685	Mycobacterium tuberculosis	PPE FAMILY PROTEIN	II
18699	Mycobacterium bovis BCG	secreted antigen 85-B fbpB	II
18700	Mycobacterium tuberculosis	Antigen 85-B	II
18781	Mycobacterium bovis BCG	Immunogenic protein MPB70 precursor	II

18896	Mycobacterium tuberculosis	Antigen 85-A precursor	II
18897	Mycobacterium bovis BCG	Antigen 85-B precursor	II
18899	Mycobacterium bovis BCG	secreted antigen 85-B fbpB	II
18900	Mycobacterium bovis	alpha-antigen	II
18901	Mycobacterium bovis	alpha-antigen	II
18914	Mycobacterium tuberculosis	Lipoprotein lpqH precursor	II
18959	Mycobacterium tuberculosis	60 KDA CHAPERONIN 2 GROEL2	II
19050	Mycobacterium bovis BCG	Immunogenic protein MPB70 precursor	II
19282	Mycobacterium bovis	PE-PGRS FAMILY PROTEIN	II
19348	Mycobacterium tuberculosis	IMMUNOGENIC PROTEIN MPT64 (ANTIGEN MPT64/MPB64)	II
19359	Mycobacterium tuberculosis	IMMUNOGENIC PROTEIN MPT64 (ANTIGEN MPT64/MPB64)	II
19646	Mycobacterium tuberculosis	Antigen 85-A precursor	II
19860	Mycobacterium tuberculosis	PPE FAMILY PROTEIN	II
19989	Mycobacterium bovis BCG	Antigen 85-B precursor	II
20101	Mycobacterium bovis BCG	Antigen 85-B precursor	II
20242	Mycobacterium tuberculosis	6 kDa early secretory antigenic target	II
20281	Mycobacterium tuberculosis H37Rv	PPE FAMILY PROTEIN	II
20662	Mycobacterium tuberculosis	14 kDa antigen	II
20670	Mycobacterium tuberculosis	6 kDa early secretory antigenic target	II
20707	Mycobacterium tuberculosis H37Rv	MCE-FAMILY PROTEIN MCE4C	II
20744	Mycobacterium bovis BCG	Immunogenic protein MPB70 precursor	II
20820	Mycobacterium sp.	60 KDA CHAPERONIN 2 GROEL2	II
20821	Mycobacterium tuberculosis	60 kDa chaperonin 2	II
20890	Mycobacterium tuberculosis	Lipoprotein lpqH precursor	II
20979	Mycobacterium tuberculosis	Antigen 85-A precursor	II
20997	Mycobacterium tuberculosis	PPE FAMILY PROTEIN	II
21093	Mycobacterium tuberculosis	Antigen 85-A precursor	II
21094	Mycobacterium bovis BCG	secreted antigen 85-B fbpB	II
21096	Mycobacterium tuberculosis	Antigen 85-B	II
21179	Mycobacterium tuberculosis	PPE FAMILY PROTEIN	II
21371	Mycobacterium bovis BCG	Immunogenic protein MPB70 precursor	II
21423	Mycobacterium tuberculosis H37Rv	ESAT-6-like protein esxB	II
21434	Mycobacterium tuberculosis	Phosphate-binding protein pstS 1 precursor	II
21439	Mycobacterium tuberculosis	Antigen 85-A precursor	II
21482	Mycobacterium tuberculosis	Antigen 85-A precursor	II
21483	Mycobacterium tuberculosis H37Rv	ESAT-6-like protein esxN	II
21497	Mycobacterium tuberculosis H37Rv	PPE FAMILY PROTEIN	II
21670	Mycobacterium tuberculosis	Antigen 85-A precursor	II
21765	Mycobacterium tuberculosis	mtp40 protein - Mycobacterium tuberculosis	II
21773	Mycobacterium tuberculosis	Uncharacterized transporter Rv1979c/MT2031	II
21795	Mycobacterium bovis BCG	secreted antigen 85-B fbpB	II
21796	Mycobacterium bovis BCG	Antigen 85-B precursor	II
21797	Mycobacterium tuberculosis	Antigen 85-B	II
21976	Mycobacterium tuberculosis	10 kDa culture filtrate antigen EsxB	II
22008	Mycobacterium bovis BCG	Antigen 85-B precursor	II
22252	Mycobacterium tuberculosis	10 kDa chaperonin	II
22266	Mycobacterium tuberculosis Erdman	ESAT-6-like protein esxI	II
22323	Mycobacterium tuberculosis	mtp40 protein - Mycobacterium tuberculosis	II
22351	Mycobacterium tuberculosis	PPE FAMILY PROTEIN	II
22531	Mycobacterium tuberculosis	PPE FAMILY PROTEIN	II
22554	Mycobacterium tuberculosis H37Rv	ESAT-6-like protein esxN	II
22633	Mycobacterium tuberculosis	Immunogenic protein MPT64 precursor	II
22657	Mycobacterium tuberculosis	PPE FAMILY PROTEIN	II
22882	Mycobacterium leprae	10 kDa chaperonin	II
22884	Mycobacterium tuberculosis	10 kDa chaperonin	II
23155	Mycobacterium tuberculosis	Immunogenic protein MPT70 precursor	II
23487	Mycobacterium tuberculosis	Lipoprotein lpqH precursor	II
23735	Mycobacterium tuberculosis	Phosphate-binding protein pstS 1 precursor	II
23837	Mycobacterium tuberculosis	Phosphate-binding protein pstS 1 precursor	II
24522	Mycobacterium tuberculosis	IMMUNOGENIC PROTEIN MPT64 (ANTIGEN MPT64/MPB64)	II
24876	Mycobacterium bovis BCG	Antigen 85-B precursor	II
25269	Mycobacterium tuberculosis	60 KDA CHAPERONIN 2 GROEL2	II
25315	Mycobacterium tuberculosis	19 KDA LIPOPROTEIN ANTIGEN PRECURSOR LPQH	II
25363	Mycobacterium tuberculosis	Antigen 85-A precursor	II
25561	Mycobacterium tuberculosis	Immunogenic protein MPT70 precursor	II
25716	Mycobacterium tuberculosis	6 kDa early secretory antigenic target	II
26269	Mycobacterium tuberculosis	Antigen 85-B precursor	II
26654	Mycobacterium tuberculosis H37Rv	PUTATIVE ESAT-6 LIKE PROTEIN ESXL (ESAT-6 LIKE PROTEIN 4)	II
26672	Mycobacterium tuberculosis Erdman	ESAT-6-like protein esxI	II
26805	Mycobacterium tuberculosis	Immunogenic protein MPT64 precursor	II
26949	Mycobacterium bovis BCG	alpha-antigen	II
27389	Mycobacterium tuberculosis	14 kDa antigen	II
27635	Mycobacterium tuberculosis	mtp40 protein - Mycobacterium tuberculosis	II

27981	Mycobacterium tuberculosis	Immunogenic protein MPT64 precursor	II
28304	Mycobacterium tuberculosis	IMMUNOGENIC PROTEIN MPT63	II
28327	Mycobacterium tuberculosis	Phosphate-binding protein pstS 1 precursor	II
28364	Mycobacterium tuberculosis	ESAT-6-like protein esxB	II
28367	Mycobacterium tuberculosis H37Rv	ESAT-6-like protein esxB	II
28470	Mycobacterium tuberculosis H37Rv	6 kDa early secretory antigenic target	II
28506	Mycobacterium tuberculosis	10 kDa culture filtrate antigen EsxB	II
28594	Mycobacterium tuberculosis	Immunogenic protein MPT64 precursor	II
29256	Mycobacterium tuberculosis	Uncharacterized transporter Rv1979c/MT2031	II
29423	Mycobacterium tuberculosis H37Rv	ESAT-6-like protein esxN	II
29574	Mycobacterium tuberculosis H37Rv	ESAT-6-like protein esxN	II
31342	Mycobacterium bovis T/91/1378	10 kDa chaperonin	II
31443	Mycobacterium tuberculosis	Lipoprotein lpqH precursor	II
31838	Mycobacterium tuberculosis	Putative lipoprotein lppX precursor	II
32215	Mycobacterium bovis BCG	Antigen 85-B precursor	II
32668	Mycobacterium tuberculosis	Putative lipoprotein lppX precursor	II
32692	Mycobacterium tuberculosis	60 kDa chaperonin 2	II
32898	Mycobacterium bovis BCG	secreted antigen 85-B fbpB	II
33162	Mycobacterium tuberculosis	10 kDa chaperonin	II
33402	Mycobacterium tuberculosis	IMMUNOGENIC PROTEIN MPT64 (ANTIGEN MPT64/MPB64)	II
33707	Mycobacterium leprae	60 kDa chaperonin 2	II
33951	Mycobacterium tuberculosis	60 kDa chaperonin 2	II
33972	Mycobacterium tuberculosis	Putative lipoprotein lppX precursor	II
34174	Mycobacterium tuberculosis H37Rv	ESAT-6-like protein esxN	II
34232	Mycobacterium tuberculosis	Lipoprotein lpqH precursor	II
34293	Mycobacterium bovis BCG	secreted antigen 85-B fbpB	II
34437	Mycobacterium tuberculosis	10 kDa chaperonin	II
34667	Mycobacterium tuberculosis H37Rv	ESAT-6-like protein esxN	II
34775	Mycobacterium bovis BCG	SECRETED ANTIGEN 85-B FBPB	II
34823	Mycobacterium tuberculosis	Secreted antigen Ag85A	II
34873	Mycobacterium bovis BCG	secreted antigen 85-B fbpB	II
34928	Mycobacterium tuberculosis	10 kDa culture filtrate antigen EsxB	II
34949	Mycobacterium tuberculosis	6 kDa early secretory antigenic target	II
35139	Mycobacterium tuberculosis	6 kDa early secretory antigenic target	II
35140	Mycobacterium tuberculosis H37Rv	ESAT-6-like protein esxB	II
35316	Mycobacterium tuberculosis	10 kDa chaperonin	II
35391	Mycobacterium tuberculosis	10 kDa chaperonin	II
35403	Mycobacterium tuberculosis	60 KDA CHAPERONIN 2 GROEL2	II
35437	Mycobacterium tuberculosis	PPE FAMILY PROTEIN	II
35740	Mycobacterium tuberculosis	PPE family protein	II
35787	Mycobacterium tuberculosis	14 kDa antigen	II
35819	Mycobacterium tuberculosis	PPE FAMILY PROTEIN	II
36046	Mycobacterium tuberculosis	Putative lipoprotein lppX precursor	II
36536	Mycobacterium bovis BCG	Antigen 85-B precursor	II
37041	Mycobacterium tuberculosis H37Rv	10 kDa culture filtrate antigen EsxB	II
37059	Mycobacterium tuberculosis	Immunogenic protein MPT64 precursor	II
37154	Mycobacterium tuberculosis	PPE FAMILY PROTEIN	II
37173	Mycobacterium bovis BCG	alpha-antigen	II
37668	Mycobacterium tuberculosis	60 kDa chaperonin 2	II
37874	Mycobacterium bovis BCG	alpha-antigen	II
37930	Mycobacterium tuberculosis	60 kDa chaperonin 2	II
38059	Mycobacterium sp. JLS	Cell divisionFtsK/SpoIIIE precursor	II
38169	Mycobacterium bovis BCG	Antigen 85-B precursor	II
38334	Mycobacterium bovis BCG	Immunogenic protein MPB70 precursor	II
38492	Mycobacterium tuberculosis	PPE FAMILY PROTEIN	II
38749	Mycobacterium bovis BCG	SECRETED ANTIGEN 85-B FBPB	II
38916	Mycobacterium tuberculosis	60 KDA CHAPERONIN 2 GROEL2	II
39010	Mycobacterium bovis BCG	secreted antigen 85-B fbpB	II
39011	Mycobacterium tuberculosis	Antigen 85-A precursor	II
39169	Mycobacterium tuberculosis	14 kDa antigen	II
39170	Mycobacterium tuberculosis	14 kDa antigen	II
39281	Mycobacterium bovis BCG	Antigen 85-B precursor	II
39282	Mycobacterium bovis	Antigen 85-B	II
39354	Mycobacterium tuberculosis H37Rv	PPE FAMILY PROTEIN	II
39408	Mycobacterium tuberculosis	Putative lipoprotein lppX precursor	II
39449	Mycobacterium bovis BCG	SECRETED ANTIGEN 85-B FBPB	II
39498	Mycobacterium bovis BCG	alpha-antigen	II
39813	Mycobacterium tuberculosis Erdman	ESAT-6-like protein esxI	II
40162	Mycobacterium tuberculosis	Antigen 85-A precursor	II
40164	Mycobacterium bovis BCG	secreted antigen 85-B fbpB	II
40165	Mycobacterium tuberculosis	Antigen 85-B	II
41001	Mycobacterium tuberculosis	Phosphate-binding protein pstS 1 precursor	II
41030	Mycobacterium tuberculosis	10 KDA CULTURE FILTRATE ANTIGEN ESXB (LHP) (CFP10)	II

41031	Mycobacterium tuberculosis H37Rv	10 KDA CULTURE FILTRATE ANTIGEN ESXB (LHP) (CFP10)	II
41060	Mycobacterium tuberculosis	60 KDA CHAPERONIN 2 GROEL2	II
41064	Mycobacterium tuberculosis	60 KDA CHAPERONIN 2 GROEL2	II
41068	Mycobacterium tuberculosis	60 KDA CHAPERONIN 2 GROEL2	II
41088	Mycobacterium bovis	ESAT-6-like protein esxR	II
41089	Mycobacterium tuberculosis	ESAT-6-like protein esxH	II
41099	Mycobacterium tuberculosis	Lipoprotein lpqH precursor	II
41161	Mycobacterium tuberculosis	14 kDa antigen	II
41708	Mycobacterium bovis BCG	SECRETED ANTIGEN 85-B FBPB	II
41764	Mycobacterium tuberculosis Erdman	antigen Mtb9.9B	II
41765	Mycobacterium tuberculosis H37Rv	ESAT-6-like protein esxN	II
41766	Mycobacterium tuberculosis H37Rv	Putative ESAT-6-like protein 6	II
41840	Mycobacterium tuberculosis	Phosphate-binding protein pstS 1 precursor	II
41872	Mycobacterium tuberculosis	Antigen 85-A precursor	II
41889	Mycobacterium bovis BCG	Immunogenic protein MPB70 precursor	II
41943	Mycobacterium tuberculosis	ESAT-6-like protein esxH	II
41948	Mycobacterium tuberculosis	mtp40 protein - Mycobacterium tuberculosis	II
41952	Mycobacterium tuberculosis	mtp40 protein - Mycobacterium tuberculosis	II
42102	Mycobacterium tuberculosis H37Rv	ESAT-6-like protein esxR	II
42195	Mycobacterium tuberculosis	Putative lipoprotein lppX precursor	II
42633	Mycobacterium tuberculosis	19 KDA LIPOPROTEIN ANTIGEN PRECURSOR LPQH	II
42638	Mycobacterium bovis	ESAT-6-like protein esxH	II
42640	Mycobacterium tuberculosis H37Rv	ESAT-6-like protein esxR	II
42642	Mycobacterium tuberculosis H37Rv	ESAT-6-like protein esxQ	II
42739	Mycobacterium tuberculosis H37Rv	ESAT-6-like protein esxQ	II
42789	Mycobacterium bovis BCG	SECRETED ANTIGEN 85-B FBPB	II
42795	Mycobacterium bovis	6 kDa early secretory antigenic target	II
42797	Mycobacterium tuberculosis	6 kDa early secretory antigenic target	II
42798	Mycobacterium tuberculosis H37Rv	6 kDa early secretory antigenic target	II
42814	Mycobacterium tuberculosis H37Rv	ESAT-6-like protein esxN	II
43166	Mycobacterium tuberculosis H37Rv	ESAT-6-like protein esxN	II
43210	Mycobacterium tuberculosis H37Rv	6 kDa early secretory antigenic target ESXA (ESAT-6)	II
43331	Mycobacterium bovis BCG	secreted antigen 85-B fbpB	II
43332	Mycobacterium tuberculosis	Antigen 85-B	II
43447	Mycobacterium tuberculosis	Immunogenic protein MPT64 precursor	II
43504	Mycobacterium tuberculosis	Antigen 85-A precursor	II
43513	Mycobacterium bovis BCG	secreted antigen 85-B fbpB	II
43514	Mycobacterium tuberculosis	Antigen 85-B	II
43670	Mycobacterium bovis BCG	Antigen 85-B precursor	II
43881	Mycobacterium tuberculosis	Phosphate-binding protein pstS 1 precursor	II
43948	Mycobacterium bovis BCG	alpha-antigen	II
43949	Mycobacterium bovis AN5	alpha-antigen	II
44020	Mycobacterium tuberculosis	Phosphate-binding protein pstS 1 precursor	II
44100	Mycobacterium tuberculosis	Antigen 85-A precursor	II
44231	Mycobacterium tuberculosis H37Rv	PPE FAMILY PROTEIN	II
44381	Mycobacterium tuberculosis	Immunogenic protein MPT64 precursor	II
44653	Mycobacterium tuberculosis	6 kDa early secretory antigenic target	II
45249	Mycobacterium bovis BCG	secreted antigen 85-B fbpB	II
45250	Mycobacterium tuberculosis	Antigen 85-B	II
46500	Mycobacterium tuberculosis	6 kDa early secretory antigenic target	II
46501	Mycobacterium tuberculosis	6 kDa early secretory antigenic target	II
46788	Mycobacterium bovis BCG	secreted antigen 85-B fbpB	II
47002	Mycobacterium bovis BCG	SECRETED ANTIGEN 85-B FBPB	II
47702	Mycobacterium bovis BCG	secreted antigen 85-B fbpB	II
48458	Mycobacterium tuberculosis	Immunogenic protein MPT64 precursor	II
48624	Mycobacterium bovis BCG	SECRETED ANTIGEN 85-B FBPB	II
49152	Mycobacterium bovis BCG	secreted antigen 85-B fbpB	II
49254	Mycobacterium tuberculosis	14 kDa antigen	II
49280	Mycobacterium tuberculosis H37Rv	MCE-FAMILY PROTEIN MCE2A	II
49420	Mycobacterium bovis BCG	secreted antigen 85-B fbpB	II
49421	Mycobacterium tuberculosis	Antigen 85-B precursor	II
49440	Mycobacterium bovis BCG	secreted antigen 85-B fbpB	II
49635	Mycobacterium bovis BCG	Immunogenic protein MPB70 precursor	II
49699	Mycobacterium tuberculosis	Antigen 85-A precursor	II
49862	Mycobacterium tuberculosis	Secreted antigen Ag85A	II
49870	Mycobacterium tuberculosis	Antigen 85-A precursor	II
49871	Mycobacterium bovis BCG	secreted antigen 85-B fbpB	II
49872	Mycobacterium tuberculosis	Antigen 85-B	II
49925	Mycobacterium tuberculosis	Immunogenic protein MPT64 precursor	II
50318	Mycobacterium tuberculosis	6 kDa early secretory antigenic target	II
50366	Mycobacterium tuberculosis	PPE FAMILY PROTEIN	II
50437	Mycobacterium bovis BCG	alpha-antigen	II
50438	Mycobacterium bovis AN5	alpha-antigen	II

50444	Mycobacterium tuberculosis	Antigen 85-A precursor	II
50589	Mycobacterium tuberculosis H37Rv	ESAT-6-like protein esxN	II
50756	Mycobacterium tuberculosis H37Rv	ESAT-6-like protein esxN	II
50778	Mycobacterium bovis AN5	protein or antigen 85-B	II
50860	Mycobacterium tuberculosis Erdman	ESAT-6-like protein esxI	II
50966	Mycobacterium tuberculosis	Phosphate-binding protein pstS 1 precursor	II
50981	Mycobacterium tuberculosis	6 kDa early secretory antigenic target	II
51114	Mycobacterium bovis BCG	secreted antigen 85-B fbpB	II
51367	Mycobacterium tuberculosis	PPE FAMILY PROTEIN	II
51633	Mycobacterium tuberculosis	lipoprotein lppO	II
51643	Mycobacterium tuberculosis	Immunogenic protein MPT64 precursor	II
52024	Mycobacterium bovis BCG	SECRETED ANTIGEN 85-B FBPB	II
52025	Mycobacterium tuberculosis	Antigen 85-B precursor	II
52030	Mycobacterium tuberculosis	Antigen 85-A precursor	II
52626	Mycobacterium bovis BCG	Immunogenic protein MPB70 precursor	II
52668	Mycobacterium bovis BCG	alpha-antigen	II
52690	Mycobacterium tuberculosis	10 kDa culture filtrate antigen EsxB	II
52691	Mycobacterium tuberculosis H37Rv	ESAT-6-like protein esxB	II
53172	Mycobacterium tuberculosis	Putative lipoprotein lppX precursor	II
53312	Mycobacterium tuberculosis H37Rv	ESAT-6-like protein esxQ	II
53355	Mycobacterium tuberculosis	14 kDa antigen	II
53356	Mycobacterium tuberculosis	14 kDa antigen	II
53370	Mycobacterium tuberculosis	IMMUNOGENIC PROTEIN MPT64	II
53833	Mycobacterium tuberculosis H37Rv	PPE FAMILY PROTEIN	II
53974	Mycobacterium tuberculosis H37Rv	ESAT-6-like protein esxQ	II
54355	Mycobacterium bovis BCG	60 KDA chaperonin 2 groel2	II
54365	Mycobacterium bovis BCG	secreted antigen 85-B fbpB	II
54516	Mycobacterium tuberculosis	14 kDa antigen	II
54829	Mycobacterium bovis BCG	secreted antigen 85-B fbpB	II
54977	Mycobacterium bovis BCG	Antigen 85-B precursor	II
54988	Mycobacterium tuberculosis H37Rv	ESAT-6-like protein esxN	II
55157	Mycobacterium tuberculosis H37Rv	PPE FAMILY PROTEIN	II
55158	Mycobacterium tuberculosis H37Rv	PPE FAMILY PROTEIN	II
55394	Mycobacterium tuberculosis H37Rv	ESAT-6-like protein esxB	II
55882	Mycobacterium tuberculosis	14 kDa antigen	II
56043	Mycobacterium tuberculosis	14 kDa antigen	II
56836	Mycobacterium tuberculosis H37Rv	6 kDa early secretory antigenic target	II
56884	Mycobacterium tuberculosis	Antigen 85-A precursor	II
56894	Mycobacterium bovis BCG	secreted antigen 85-B fbpB	II
56895	Mycobacterium tuberculosis	Antigen 85-B	II
56994	Mycobacterium tuberculosis	Antigen 85-C precursor	II
57047	Mycobacterium tuberculosis	ESAT-6-like protein esxH	II
57048	Mycobacterium tuberculosis H37Rv	ESAT-6-like protein esxR	II
57256	Mycobacterium tuberculosis	Putative lipoprotein lppX precursor	II
57415	Mycobacterium tuberculosis	14 kDa antigen	II
57417	Mycobacterium tuberculosis	14 kDa antigen	II
57719	Mycobacterium tuberculosis	Immunogenic protein MPT70 precursor	II
58022	Mycobacterium tuberculosis	Lipoprotein lpqH precursor	II
58051	Mycobacterium bovis BCG	Antigen 85-B precursor	II
58124	Mycobacterium bovis BCG	Antigen 85-B precursor	II
58144	Mycobacterium tuberculosis	Phosphate-binding protein pstS 1 precursor	II
58225	Mycobacterium tuberculosis H37Rv	6 kDa early secretory antigenic target	II
58475	Mycobacterium tuberculosis	Putative lipoprotein lppX precursor	II
59629	Mycobacterium bovis BCG	SECRETED ANTIGEN 85-B FBPB	II
59748	Mycobacterium tuberculosis H37Rv	ESAT-6-like protein esxR	II
59861	Mycobacterium bovis BCG	alpha-antigen	II
60061	Mycobacterium tuberculosis	Phosphate-binding protein pstS 1 precursor	II
60262	Mycobacterium tuberculosis	Antigen 85-B precursor	II
60263	Mycobacterium tuberculosis	MPT51/MPB51 antigen precursor	II
60442	Mycobacterium bovis BCG	SECRETED ANTIGEN 85-B FBPB	II
61016	Mycobacterium bovis BCG	secreted antigen 85-B fbpB	II
61178	Mycobacterium bovis BCG	Immunogenic protein MPB70 precursor	II
61240	Mycobacterium bovis BCG	alpha-antigen	II
61578	Mycobacterium tuberculosis	6 kDa early secretory antigenic target	II
61782	Mycobacterium tuberculosis	ESAT-6-like protein esxB	II
61834	Mycobacterium tuberculosis	Immunogenic protein MPT64 precursor	II
61914	Mycobacterium tuberculosis	Putative lipoprotein lppX precursor	II
62057	Mycobacterium bovis	60 KDA CHAPERONIN 2 GROEL2	II
62062	Mycobacterium tuberculosis	14 kDa antigen	II
62187	Mycobacterium tuberculosis	Putative lipoprotein lppX precursor	II
62899	Mycobacterium tuberculosis	10 kDa chaperonin	II
62989	Mycobacterium tuberculosis	COG0234: Co-chaperonin GroES (HSP10)	II
62998	Mycobacterium tuberculosis	Lipoprotein lpqH precursor	II

63010	Mycobacterium tuberculosis	6 kDa early secretory antigenic target	II
63011	Mycobacterium tuberculosis H37Rv	6 kDa early secretory antigenic target	II
63662	Mycobacterium tuberculosis	60 kDa chaperonin 2	II
63663	Mycobacterium bovis	60 kDa chaperonin 2	II
63956	Mycobacterium bovis BCG	Antigen 85-B precursor	II
63960	Mycobacterium tuberculosis	Phosphate-binding protein pstS 1 precursor	II
63966	Mycobacterium bovis	Antigen 85-B	II
64077	Mycobacterium bovis BCG	SECRETED ANTIGEN 85-B FBPB	II
64078	Mycobacterium bovis BCG	Antigen 85-B precursor	II
64079	Mycobacterium tuberculosis	Antigen 85-B	II
64081	Mycobacterium tuberculosis	Antigen 85-A precursor	II
64580	Mycobacterium tuberculosis	Putative lipoprotein lppX precursor	II
64822	Mycobacterium tuberculosis	PPE FAMILY PROTEIN	II
65499	Mycobacterium bovis BCG	Antigen 85-B precursor	II
65767	Mycobacterium tuberculosis	PPE FAMILY PROTEIN	II
65912	Mycobacterium tuberculosis	PPE FAMILY PROTEIN	II
65965	Mycobacterium tuberculosis	Immunogenic protein MPT64 precursor	II
66693	Mycobacterium tuberculosis	mtp40 protein - Mycobacterium tuberculosis	II
67156	Mycobacterium tuberculosis	14 kDa antigen	II
67211	Mycobacterium tuberculosis	Putative lipoprotein lppX precursor	II
67233	Mycobacterium bovis BCG	Immunogenic protein MPB70 precursor	II
67588	Mycobacterium tuberculosis H37Rv	MCE-FAMILY PROTEIN MCE2A	II
67607	Mycobacterium tuberculosis	19 KDA LIPOPROTEIN ANTIGEN PRECURSOR LPQH	II
67657	Mycobacterium tuberculosis	10 kDa chaperonin	II
67695	Mycobacterium bovis BCG	SECRETED ANTIGEN 85-B FBPB	II
67697	Mycobacterium tuberculosis	Antigen 85-B	II
67783	Mycobacterium tuberculosis	Lipoprotein lpqH precursor	II
68189	Mycobacterium tuberculosis	60 KDA CHAPERONIN 2 GROEL2	II
68285	Mycobacterium tuberculosis	PPE FAMILY PROTEIN	II
68529	Mycobacterium bovis BCG	Antigen 85-B precursor	II
69128	Mycobacterium tuberculosis	PPE family protein	II
69308	Mycobacterium tuberculosis	Lipoprotein lpqH	II
69467	Mycobacterium tuberculosis H37Rv	ESAT-6-like protein esxQ	II
69796	Mycobacterium tuberculosis	PPE FAMILY PROTEIN	II
70124	Mycobacterium tuberculosis	Immunogenic protein MPT70 precursor	II
70687	Mycobacterium tuberculosis	10 kDa culture filtrate antigen EsxB	II
70903	Mycobacterium tuberculosis	IMMUNOGENIC PROTEIN MPT63	II
70980	Mycobacterium tuberculosis	IMMUNOGENIC PROTEIN MPT64	II
71346	Mycobacterium tuberculosis	Lipoprotein lpqH precursor	II
71492	Mycobacterium tuberculosis	6 kDa early secretory antigenic target	II
71596	Mycobacterium tuberculosis	60 KDA CHAPERONIN 2 GROEL2	II
71945	Mycobacterium tuberculosis	PPE FAMILY PROTEIN	II
72008	Mycobacterium tuberculosis	60 KDA CHAPERONIN 2 GROEL2	II
72085	Mycobacterium bovis BCG	SECRETED ANTIGEN 85-B FBPB	II
72294	Mycobacterium tuberculosis	10 kDa chaperonin	II
72312	Mycobacterium tuberculosis	Antigen 85-A precursor	II
72313	Mycobacterium bovis BCG	SECRETED ANTIGEN 85-B FBPB	II
72314	Mycobacterium tuberculosis	Antigen 85-B	II
72323	Mycobacterium tuberculosis	Immunogenic protein MPT64 precursor	II
72514	Mycobacterium bovis BCG	secreted antigen 85-B fbpB	II
72741	Mycobacterium tuberculosis	mtp40 protein - Mycobacterium tuberculosis	II
72888	Mycobacterium tuberculosis	6 kDa early secretory antigenic target	II
73049	Mycobacterium tuberculosis CDC1551	proteinase	II
73069	Mycobacterium tuberculosis H37Rv	Probable integral membrane cytochrome D ubiquinol oxidase (subunit I)	II
73235	Mycobacterium bovis BCG	Antigen 85-B precursor	II
73294	Mycobacterium bovis BCG	Antigen 85-B precursor	II
73361	Mycobacterium tuberculosis H37Rv	ESAT-6-like protein esxR	II
73362	Mycobacterium tuberculosis	ESAT-6-like protein esxH	II
73378	Mycobacterium tuberculosis	Phosphate-binding protein pstS 1 precursor	II
73524	Mycobacterium tuberculosis H37Rv	MCE-FAMILY PROTEIN MCE4C	II
73578	Mycobacterium tuberculosis	Antigen 85-A precursor	II
73593	Mycobacterium tuberculosis	IMMUNOGENIC PROTEIN MPT64	II
73690	Mycobacterium tuberculosis	60 kDa chaperonin 2	II
73784	Mycobacterium tuberculosis	14 kDa antigen	II
73962	Mycobacterium tuberculosis	60 kDa chaperonin	II
73964	Mycobacterium tuberculosis	60 KDA CHAPERONIN 2 GROEL2	II
74486	Mycobacterium bovis BCG	alpha-antigen	II
74769	Mycobacterium bovis BCG	Antigen 85-B precursor	II
74889	Mycobacterium bovis BCG	Antigen 85-B precursor	II
75152	Mycobacterium leprae	10 kDa chaperonin	II
75153	Mycobacterium tuberculosis	10 kDa chaperonin	II
75172	Mycobacterium tuberculosis	Immunogenic protein MPT64 precursor	II
75463	Mycobacterium tuberculosis	ESAT-6-like protein esxH	II

75495	Mycobacterium tuberculosis	6 kDa early secretory antigenic target	II
75496	Mycobacterium tuberculosis H37Rv	ESAT-6-like protein esxR	II
75786	Mycobacterium leprae	10 kDa chaperonin	II
76454	Mycobacterium bovis BCG	secreted antigen 85-B fbpB	II
76583	Mycobacterium bovis BCG	secreted antigen 85-B fbpB	II
76584	Mycobacterium tuberculosis	Antigen 85-B	II
78930	Mycobacterium tuberculosis	60 kDa chaperonin 2	II
78960	Mycobacterium tuberculosis	60 kDa chaperonin 2	II
78980	Mycobacterium tuberculosis	60 kDa chaperonin 2	II
78996	Mycobacterium tuberculosis	60 kDa chaperonin 2	II
79000	Mycobacterium tuberculosis	60 kDa chaperonin 2	II
79056	Mycobacterium bovis	heat shock protein 65	II
79110	Mycobacterium tuberculosis	60 kDa chaperonin 2	II
80451	Mycobacterium tuberculosis H37Rv	early secretory antigenic target, 6 kDa	II
82519	Mycobacterium tuberculosis H37Rv	10 kDa culture filtrate antigen EsxB	II
82520	Mycobacterium tuberculosis H37Rv	10 kDa culture filtrate antigen EsxB	II
85043	Mycobacterium tuberculosis H37Rv	10 kDa culture filtrate antigen EsxB	II
85635	Mycobacterium tuberculosis H37Rv	early secretory antigenic target, 6 kDa	II
85735	Mycobacterium tuberculosis H37Rv	early secretory antigenic target, 6 kDa	II
100510	Mycobacterium tuberculosis	PPE family protein	II
100593	Mycobacterium tuberculosis	PPE family protein	II
102321	Mycobacterium tuberculosis	IMMUNOGENIC PROTEIN MPT63	II
102375	Mycobacterium tuberculosis	IMMUNOGENIC PROTEIN MPT63	II
102640	Mycobacterium tuberculosis	IMMUNOGENIC PROTEIN MPT63	II
102648	Mycobacterium tuberculosis	IMMUNOGENIC PROTEIN MPT63	II
102708	Mycobacterium tuberculosis	IMMUNOGENIC PROTEIN MPT63	II
102824	Mycobacterium tuberculosis	IMMUNOGENIC PROTEIN MPT63	II
102870	Mycobacterium tuberculosis	IMMUNOGENIC PROTEIN MPT63	II
102929	Mycobacterium tuberculosis	IMMUNOGENIC PROTEIN MPT63	II
102963	Mycobacterium tuberculosis	IMMUNOGENIC PROTEIN MPT63	II
102966	Mycobacterium tuberculosis	IMMUNOGENIC PROTEIN MPT63	II
103408	Mycobacterium tuberculosis	Chaperone protein dnaK	II
103416	Mycobacterium tuberculosis	Antigen 85-A precursor	II
103423	Mycobacterium tuberculosis	Antigen 85-A precursor	II
105967	Mycobacterium tuberculosis	19 KDA LIPOPROTEIN ANTIGEN PRECURSOR LPQH	II
105976	Mycobacterium tuberculosis	19 KDA LIPOPROTEIN ANTIGEN PRECURSOR LPQH	II
106686	Mycobacterium tuberculosis	6 kDa early secretory antigenic target	II
110765	Mycobacterium tuberculosis	hypothetical protein	II
110788	Mycobacterium tuberculosis	hypothetical protein	II
110790	Mycobacterium tuberculosis	hypothetical protein	II
110791	Mycobacterium tuberculosis	hypothetical protein	II
110822	Mycobacterium tuberculosis	hypothetical protein	II
110827	Mycobacterium tuberculosis	hypothetical protein	II
110831	Mycobacterium tuberculosis	hypothetical protein	II
110838	Mycobacterium tuberculosis	hypothetical protein	II
110878	Mycobacterium tuberculosis	hypothetical protein	II
110881	Mycobacterium tuberculosis	hypothetical protein	II
110905	Mycobacterium tuberculosis	hypothetical protein	II
110907	Mycobacterium tuberculosis	hypothetical protein	II
110925	Mycobacterium tuberculosis	hypothetical protein	II
110928	Mycobacterium tuberculosis	hypothetical protein	II
110929	Mycobacterium tuberculosis	hypothetical protein	II
110930	Mycobacterium tuberculosis	hypothetical protein	II
110935	Mycobacterium tuberculosis	hypothetical protein	II
110937	Mycobacterium tuberculosis	hypothetical protein	II
110944	Mycobacterium tuberculosis	hypothetical protein	II
110953	Mycobacterium tuberculosis	hypothetical protein	II
110966	Mycobacterium tuberculosis	hypothetical protein	II
110973	Mycobacterium tuberculosis	hypothetical protein	II
110998	Mycobacterium tuberculosis	hypothetical protein	II
111006	Mycobacterium tuberculosis	hypothetical protein	II
111011	Mycobacterium tuberculosis	hypothetical protein	II
111014	Mycobacterium tuberculosis	hypothetical protein	II
111023	Mycobacterium tuberculosis	hypothetical protein	II
111047	Mycobacterium tuberculosis	hypothetical protein	II
111054	Mycobacterium tuberculosis	hypothetical protein	II
114830	Mycobacterium	60 KDA CHAPERONIN 2 GROEL2	II
114858	Mycobacterium	60 KDA CHAPERONIN 2 GROEL2	II
115927	Mycobacterium tuberculosis	hypothetical protein	II
118738	Mycobacterium tuberculosis H37Ra	PPE family protein	II
118739	Mycobacterium tuberculosis H37Ra	PPE family protein	II
118742	Mycobacterium tuberculosis H37Ra	PPE family protein	II
118747	Mycobacterium tuberculosis H37Ra	PPE family protein	II

118756	Mycobacterium tuberculosis H37Ra	PPE family protein	II
118765	Mycobacterium tuberculosis H37Ra	PPE family protein	II
118792	Mycobacterium tuberculosis H37Ra	PPE family protein	II
118803	Mycobacterium tuberculosis H37Ra	PPE family protein	II
118825	Mycobacterium tuberculosis H37Ra	PPE family protein	II
118840	Mycobacterium tuberculosis H37Ra	PPE family protein	II
118849	Mycobacterium tuberculosis H37Ra	PPE family protein	II
118856	Mycobacterium tuberculosis H37Ra	PPE family protein	II
118857	Mycobacterium tuberculosis H37Ra	PPE family protein	II
118869	Mycobacterium bovis	60 kDa chaperonin 2	II
118876	Mycobacterium tuberculosis H37Ra	PPE family protein	II
118878	Mycobacterium tuberculosis H37Ra	PPE family protein	II
118880	Mycobacterium tuberculosis H37Ra	PPE family protein	II
118886	Mycobacterium tuberculosis H37Ra	PPE family protein	II
118889	Mycobacterium tuberculosis H37Ra	PPE family protein	II
118890	Mycobacterium tuberculosis H37Ra	PPE family protein	II
118900	Mycobacterium tuberculosis H37Ra	PPE family protein	II
118920	Mycobacterium tuberculosis H37Ra	PPE family protein	II
118931	Mycobacterium tuberculosis H37Ra	PPE family protein	II
118932	Mycobacterium tuberculosis H37Ra	PPE family protein	II
118934	Mycobacterium tuberculosis H37Ra	PPE family protein	II
118936	Mycobacterium tuberculosis H37Ra	PPE family protein	II
118939	Mycobacterium tuberculosis H37Ra	PPE family protein	II
118942	Mycobacterium tuberculosis H37Ra	PPE family protein	II
118990	Mycobacterium tuberculosis H37Ra	PPE family protein	II
119007	Mycobacterium tuberculosis H37Ra	PPE family protein	II
119019	Mycobacterium tuberculosis H37Ra	PPE family protein	II
119020	Mycobacterium tuberculosis H37Ra	PPE family protein	II
119021	Mycobacterium tuberculosis H37Ra	PPE family protein	II
119028	Mycobacterium tuberculosis H37Ra	PPE family protein	II
119033	Mycobacterium tuberculosis H37Ra	PPE family protein	II
119035	Mycobacterium tuberculosis H37Ra	PPE family protein	II
119047	Mycobacterium tuberculosis H37Ra	PPE family protein	II
119050	Mycobacterium tuberculosis H37Ra	PPE family protein	II
119056	Mycobacterium tuberculosis H37Ra	PPE family protein	II
125165	Mycobacterium tuberculosis H37Rv	PPE family protein	II
125317	Mycobacterium tuberculosis	KatG	II
136442	Mycobacterium tuberculosis	Immunogenic protein MPT64 precursor	II
142231	Mycobacterium tuberculosis	Lipoprotein lpqH precursor	II
142232	Mycobacterium tuberculosis	POSSIBLE CONSERVED TRANSMEMBRANE PROTEIN	II
142233	Mycobacterium tuberculosis	ATP-dependent helicase	II
145826	Mycobacterium tuberculosis	6 kDa early secretory antigenic target	II
A|ISPRTLNAW	HIV	p24	I
AADTGNSSQ	HIV	p17	I
AAENLWVTVYY	HIV	gp160	I
AAEWDRLHPVH	HIV	p24	I
AAFDLSFFL	HIV	Nef	I
AALDLSHFL	HIV	Nef	I
AALDMSHFL	HIV	Nef	I
AAVDLSHFL	HIV	Nef	I
AAVDLSHFLK	HIV	Nef	I
AAVDLSHFLKEK	HIV	Nef	I
AAVKAACWW	HIV	Integrase	I
ACQGVGGPGHK	HIV	p24	I
ACQGVGGPSHK	HIV	p24	I
ACVPTDPNP	HIV	gp160	I
AEAMSQVTNS	HIV	p2p7p1p6	I
AENLVWTVYY	HIV	gp160	I
AENLWVTVY	HIV	gp160	I
AENLWVTVYY	HIV	gp160	I
AEQASADVKNW	HIV	p24	I
AEQASQDVKNW	HIV	p24	I
AEQASQEVKNW	HIV	p24	I
AEQASQEVKNWM	HIV	p24	I
AEQATQDVKNW	HIV	p24	I
AESFRFEET	HIV	p2p7p1p6	I
AETFYVDGA	HIV	RT	I
AETFYVDGAAN	HIV	RT	I
AETGQETAY	HIV	Integrase	I
AETGQETAYY	HIV	Integrase	I
AEWDRLHPV	HIV	p24	I
AEWDRVHPV	HIV	p24	I
AFDLSFFLK	HIV	Nef	I

AFHHVAREK	HIV	Nef	I
AFHHVAREL	HIV	Nef	I
AFSPEVIPMFSALS	HIV	p24	I
AFSPEVIPMFT	HIV	p24	I
AIFQCSMTK	HIV	RT	I
AIFQSSMTK	HIV	RT	I
AIIRILQQL	HIV	p24	I
AIRKAILGR	HIV	Vif	I
AISPRTLNAW	HIV	p24	I
AISPRTLNAWV	HIV	p24	I
AKAYETEKHNVW	HIV	gp160	I
AKTIIVQLTEPVE	HIV	gp160	I
ALAALITPK	HIV	Vif	I
ALDLSHFL	HIV	Nef	I
ALDLSHFLK	HIV	Nef	I
ALGPGASLEEM	HIV	p24	I
ALHHVAREL	HIV	Nef	I
ALIRILQQL	HIV	Vpr	I
ALKHRAYEL	HIV	Nef	I
ALQDSGLEV	HIV	RT	I
ALSEGATPQDL	HIV	p24	I
ALSEGATPQDLNMM	HIV	p24	I
ALTSSNTAA	HIV	Nef	I
ALVEICTEL	HIV	RT	I
ALVEICTEM	HIV	RT	I
ALVEICTEMEK	HIV	RT	I
ALVEMGHHA	HIV	Vpu	I
ALVEMGHHV	HIV	Vpu	I
ALVICTEMEK	HIV	RT	I
AMQMLKDTI	HIV	p24	I
AMQMLKETI	HIV	p24	I
ANPDCKTIL	HIV	p24	I
APQGHPNNQVSI	HIV	Tat	I
APTKAKRRV	HIV	gp160	I
APTKAKRRVV	HIV	gp160	I
AQNPEIVIY	HIV	RT	I
AQNPEIVIYQY	HIV	RT	I
ARLVITTYW	HIV	Vif	I
ARRNRRRRW	HIV	Rev	I
ARVLAVERY	HIV	gp160	I
ASILRGGKLDK	HIV	p17	I
ASQEVKNWM	HIV	p24	I
ASVLSGGEL	HIV	p17	I
ATLEEMMTA	HIV	p24	I
ATLWCVHQRIDI	HIV	p17	I
ATLYCVHQK	HIV	p17	I
ATLYCVHQR	HIV	p17	I
ATPQDLNMML	HIV	p24	I
ATPQDLNTM	HIV	p24	I
ATPQDLNTMLNT	HIV	p24	I
ATQEVKNWM	HIV	p24	I
AVDLSHFLK	HIV	Nef	I
AVFIHNFKR	HIV	Integrase	I
AVFIHNFKRK	HIV	Integrase	I
AVLSVVNRV	HIV	gp160	I
AVLYCVHQR	HIV	p17	I
AVRERMRRT	HIV	Nef	I
AVRHFPRIW	HIV	Vpr	I
AVRHFRPIW	HIV	Vpr	I
AVRHRPRIW	HIV	Vpr	I
AVRIIKIL/M	HIV	Rev	I
CAPAGFAIL	HIV	gp160	I
CGKEGHIAR	HIV	p2p7p1p6	I
CKNVSTVQC	HIV	gp160	I
CLHCQVCFI	HIV	Tat	I
CPKVSFEPI	HIV	gp160	I
CQGVGGPGHK	HIV	p24	I
CRAPRKKGC	HIV	p2p7p1p6	I
CTERQANFL	HIV	p2p7p1p6	I
CTLNFPISPI	HIV	Protease-RT	I
CTNVSTVQC	HIV	gp160	I
CTPAGYAILKC	HIV	gp160	I
CTPYDINQMLNC	HIV	p24	I

CTRPNNNTRK	HIV	gp160	I
CVHQRIEIK	HIV	p17	I
DAKLIITTY	HIV	Vif	I
DAKLVITTY	HIV	Vif	I
DCKTILKAL	HIV	p24	I
DCTERQANFLG	HIV	p2p7p1p6	I
DIRQGPKEPFR	HIV	p24	I
DIYKRWII	HIV	p24	I
DLADQLIHLY	HIV	Vif	I
DLEIGQHRTK	HIV	RT	I
DLNMMLNIV	HIV	p24	I
DLNTMLNTV	HIV	p24	I
DLNTMLNTVG	HIV	p24	I
DLSHFLKEK	HIV	Nef	I
DNLWVTVYY	HIV	gp160	I
DPEKEVLQWK	HIV	Nef	I
DPNPQEMVL	HIV	gp160	I
DPNPQEVAL	HIV	gp160	I
DPNPQEVVL	HIV	gp160	I
DQGPQREPY	HIV	Vpr	I
DRFFKTLRA	HIV	p24	I
DRFWKTLRA	HIV	p24	I
DRFYKLTRA	HIV	p24	I
DRFYKTLRA	HIV	p24	I
DRFYKTLRAE	HIV	p24	I
DRFYKTLRAEQ	HIV	p24	I
DRFYKTRAE	HIV	p24	I
DSRLAFHHM	HIV	Nef	I
DSRLAFHHV	HIV	Nef	I
DTGHSNQVSQNY	HIV	p17	I
DTINEEAAEW	HIV	p24	I
DTVLEDINL	HIV	Protease	I
DTVLEDMNL	HIV	Protease	I
DTVLEEMNL	HIV	Protease	I
DTVLEEWNL	HIV	Protease	I
DTWAGVEAIIR	HIV	Vpr	I
DVKDTKEAL	HIV	p17	I
DVKQLTEVV	HIV	RT	I
DYVDRFFKTL	HIV	p24	I
DYVDRFYKT	HIV	p24	I
DYVDRFYKTL	HIV	p24	I
EAVRHFPRI	HIV	Vpr	I
EDMNLPGRW	HIV	Protease	I
EEAAEWDRL	HIV	p24	I
EEAAEWDRV	HIV	p24	I
EEGVGFPVRPQ	HIV	Nef	I
EEHEKYHSNW	HIV	Integrase	I
EEINLPGKW	HIV	Protease	I
EEKAFSPEV	HIV	p24	I
EELLKTVRL	HIV	Rev	I
EELREHLLKW	HIV	RT	I
EELRQHLLR	HIV	RT	I
EELRQHLLRW	HIV	RT	I
EELRSLYNT	HIV	p17	I
EELRSLYNTV	HIV	p17	I
EEMMTACQGVG	HIV	p24	I
EEMNLPGRW	HIV	Protease	I
EGATPQDLNMML	HIV	p24	I
EGTDRIVIEI	HIV	gp160	I
EIIGDIRQAY	HIV	gp160	I
EIKDTKEAL	HIV	p17	I
EILKEPVGHV	HIV	RT	I
EIVASCDKCQL	HIV	Integrase	I
EIYKRWIIL	HIV	p24	I
EKEGKISKI	HIV	RT	I
EKGGLEGLIHSQRR	HIV	Nef	I
EKIRLRPGGKKYKL	HIV	p17	I
ELDRWEKIRL	HIV	p17	I
ELHPDKWTV	HIV	RT	I
ELHPDRWTV	HIV	RT	I
ELKKIIGQVR	HIV	Integrase	I
ELKSLFNTI	HIV	p17	I
ELRQHLLRW	HIV	RT	I

ELRSLYNTV	HIV	p17	I
ELRSLYNTVA	HIV	p17	I
ELVNQIIEQL	HIV	RT	I
EMMTACQGV	HIV	p24	I
ENLWVTVYY	HIV	gp160	I
EPFRDYVDRF	HIV	p24	I
EPFRDYVDRFF	HIV	p24	I
EPIDKELYPL	HIV	p2p7p1p6	I
EPIDKELYPLA	HIV	p2p7p1p6	I
EPIVGAETF	HIV	RT	I
EPIVGAETFY	HIV	RT	I
EPTAPPEESF	HIV	p2p7p1p6	I
EPVDPNLEPW	HIV	Tat	I
EPVDPRLEPW	HIV	Tat	I
EQASQEVKNWMT	HIV	p24	I
EREVLVWKF	HIV	Nef	I
ERFAVNPGL	HIV	p17	I
ERILSTYLGR	HIV	Rev	I
ERQANFLGKIW	HIV	p2p7p1p6	I
ERYLKDQQL	HIV	gp160	I
ERYLKDQQLLG	HIV	gp160	I
ERYLRDQQL	HIV	gp160	I
ETAYFILKL	HIV	Integrase	I
ETFYVDGAANR	HIV	RT	I
ETGQETAY	HIV	Integrase	I
ETGQETAYY	HIV	Integrase	I
ETINEEAAEW	HIV	p24	I
ETKLGKAGY	HIV	RT	I
ETYGDTWAGV	HIV	Vpr	I
ETYGDTWTGV	HIV	Vpr	I
EVAQRAYR	HIV	gp160	I
EVFRPGGGDM	HIV	gp160	I
EVHIPLGEAR	HIV	Vif	I
EVHNVWATHA	HIV	gp160	I
EVIPMFSAL	HIV	p24	I
EVIPMFTAL	HIV	p24	I
EVKDTKEAL	HIV	p17	I
EVKNWMTETL	HIV	p24	I
EVKNWMTETLL	HIV	p24	I
EVNIVTDSQY	HIV	RT	I
EYRKILRQR	HIV	Vpu	I
FFPDWKNYT	HIV	Nef	I
FHCQVCFITK	HIV	Tat	I
FHCQVCFMTK	HIV	Tat	I
FIMIVGGLV	HIV	gp160	I
FKNLKTGKY	HIV	RT	I
FKRKGGIGGY	HIV	Integrase	I
FLGKIWPS	HIV	p2p7p1p6	I
FLGKIWPSHK	HIV	p2p7p1p6	I
FLGKIWPSYK	HIV	p2p7p1p6	I
FLKEKGGLEGL	HIV	Nef	I
FLQSRPEPTA	HIV	p2p7p1p6	I
FMKKGLGISY	HIV	Tat	I
FMTKGLGISY	HIV	Tat	I
FNCGGEFFY	HIV	gp160	I
FPDWQNYTP	HIV	Nef	I
FPISPIETV	HIV	Protease-RT	I
FPISPIETVP	HIV	Protease-RT	I
FPRIWLHGL	HIV	Vpr	I
FPRPWLHGL	HIV	Vpr	I
FPRPWLHSL	HIV	Vpr	I
FPRTWLHGL	HIV	Vpr	I
FPVKPQVPL	HIV	Nef	I
FPVRPQVPL	HIV	Nef	I
FPVRPQVPLR	HIV	Nef	I
FPVTPQVPL	HIV	Nef	I
FPVTPQVPLR	HIV	Nef	I
FPVTPQVPLRMTY	HIV	Nef	I
FQTKGLGISY	HIV	Tat	I
FRDYVDRFF	HIV	p24	I
FRDYVDRFFK	HIV	p24	I
FRDYVDRFFKAL	HIV	p24	I
FRDYVDRFYK	HIV	p24	I

FRDYVDRFYKTLRA	HIV	p24	I
FSFPQITLW	HIV	Gag_Pol_TF	I
FSPEVIPMF	HIV	p24	I
FSVPLDEDF	HIV	RT	I
FTTPDKKHQK	HIV	RT	I
FVNTPPLVK	HIV	RT	I
FYCNSTQLF	HIV	gp160	I
FYCNTTQLF	HIV	gp160	I
FYKTLRAEQ	HIV	p24	I
GAETFYVDGA	HIV	RT	I
GAFDLSFFL	HIV	Nef	I
GAFDLSFFLK	HIV	Nef	I
GALDLSFFL	HIV	Nef	I
GALDLSHFL	HIV	Nef	I
GATPQDLNMMLNIV	HIV	p24	I
GATPQDLNTMLNTV	HIV	p24	I
GDIYKRWII	HIV	p24	I
GDIYWKRWI	HIV	p24	I
GEFFYCDTS	HIV	gp160	I
GEIYKRWII	HIV	p24	I
GEIYKRWIIL	HIV	p24	I
GELDRWEKI	HIV	p17	I
GEVGFPVRPQV	HIV	Nef	I
GGHQAAMQMLK	HIV	p24	I
GGKKKYKLK	HIV	p17	I
GGKKKYKLKHIV	HIV	p17	I
GHQAAMEMLKD	HIV	p24	I
GHQAAMQML	HIV	p24	I
GHQAAMQMLKD	HIV	p24	I
GHQAAMQMLKE	HIV	p24	I
GIPHPAGLK	HIV	RT	I
GIPHPAGLKK	HIV	RT	I
GIRYPLTFGWCFK	HIV	Nef	I
GKKAIGTVL	HIV	Protease	I
GKKVSQNYPIV	HIV	p17-p24	I
GKLDAWEKIRLR	HIV	p17	I
GKLDSWEKIRLR	HIV	p17	I
GLADQLIHL	HIV	Vif	I
GLLESSEGC	HIV	p17	I
GLNKIVRMY	HIV	p24	I
GMGSPQILV	HIV	Rev	I
GNFLQSRPTAPPF	HIV	p2p7p1p6	I
GNSSQVSQNY	HIV	p17	I
GPCKNVSTVQ	HIV	gp160	I
GPGATLEEM	HIV	p24	I
GPGHKARVL	HIV	p24	I
GPGHKARVLA	HIV	p24-p2p7p1p6	I
GPGIRYPLTFGWCF	HIV	Nef	I
GPGVRYPLTF	HIV	Nef	I
GPGVRYPLTFGWCY	HIV	Nef	I
GPKESKKKVE	HIV	Tat	I
GPKVKQWPL	HIV	RT	I
GPKVKQWPLT	HIV	RT	I
GPSHKARVL	HIV	p24	I
GQGQWTYQI	HIV	RT	I
GQLDRWEKI	HIV	p17	I
GQMREPRGSDI	HIV	p24	I
GQMREPRGSDIA	HIV	p24	I
GQMVHQAISP	HIV	p24	I
GQMVHQAISPRTL	HIV	p24	I
GQMVHQAISPRTLN	HIV	p24	I
GRAFVTIGK	HIV	gp160	I
GRFPLTFGW	HIV	Nef	I
GRRGWEALK	HIV	gp160	I
GRRGWEALKY	HIV	gp160	I
GRRGWEVLKY	HIV	gp160	I
GSEELRSLY	HIV	p17	I
GTCKSVSTVQCTH	HIV	gp160	I
GTEELRSLY	HIV	p17	I
GVETFYVDGA	HIV	RT	I
GVGAASRDL	HIV	Nef	I
GVGAVSRDL	HIV	Nef	I
GVGGPGHKAR	HIV	p24	I

GYFPDWQNY	HIV	Nef	I
HAPWDVNDL	HIV	Vpu	I
HEDIISLWDQSLK	HIV	gp160	I
HFPRIWLHGLG	HIV	Vpr	I
HIGPGRAFY	HIV	gp160	I
HIVSPRCEY	HIV	Vif	I
HLEGKVILV	HIV	Integrase	I
HLKTAVQMAV	HIV	Integrase	I
HLVWASREL	HIV	p17	I
HMYISKKAK	HIV	Vif	I
HPDIVIYQY	HIV	RT	I
HPKISSEVHI	HIV	Vif	I
HPKVSSEVHI	HIV	Vif	I
HPRISSEVHI	HIV	Vif	I
HPRVSSEVHI	HIV	Vif	I
HPVHAGPIA	HIV	p24	I
HPVHAGPVA	HIV	p24	I
HPVSLHGMDDPE	HIV	Nef	I
HQAAMQMLK	HIV	p24	I
HQAAMQMLKETINE	HIV	p24	I
HQAISPRTL	HIV	p24	I
HQKEPPFLW	HIV	RT	I
HQMKDCNERQAN	HIV	p2p7p1p6	I
HQPISPRTL	HIV	p24	I
HQRIEIKDTK	HIV	p17	I
HRLRDLLLI	HIV	gp160	I
HSFNCGGEF	HIV	gp160	I
HSQRRQDIL	HIV	Nef	I
HSQRRQDILDLWIY	HIV	Nef	I
HTGERDWHL	HIV	Vif	I
HTQGYFPDW	HIV	Nef	I
HTQGYFPDWQ	HIV	Nef	I
HVASGYIEA	HIV	Integrase	I
HVPHAGPIA	HIV	p24	I
HYCAPAGFAIL	HIV	gp160	I
HYMLKHIVW	HIV	p17	I
HYMLKHLVW	HIV	p17	I
HYMLKHLVWAS	HIV	p17	I
HYMLNHIVW	HIV	p17	I
IAKNCRAPRK	HIV	p2p7p1p6	I
IAKNCRAPRKK	HIV	p2p7p1p6	I
IALESIVIW	HIV	RT	I
IAMESIVIW	HIV	RT	I
IATESIVIW	HIV	RT	I
IATLWCVHQR	HIV	p17	I
IDIKDTKEAL	HIV	p17	I
IEEKAFSPEV	HIV	p24	I
IEEKAFSPEVI	HIV	p24	I
IEELRQHLL	HIV	RT	I
IEICGHKAIG	HIV	Protease	I
IEIKDTKEAL	HIV	p17	I
IFPFRAFYA	HIV	gp160	I
IGPGRAFYAR	HIV	gp160	I
IHSISERIL	HIV	Rev	I
IHSQRRQDI	HIV	Nef	I
IIATDIQTK	HIV	Integrase	I
IILGLNKIV	HIV	p24	I
IILGLNKIVR	HIV	p24	I
IISLWDQSL	HIV	gp160	I
IKLEPVHGVY	HIV	RT	I
ILDLWIYHT	HIV	Nef	I
ILDLWVYHT	HIV	Nef	I
ILGEPPTVL	HIV	Rev	I
ILGLNKIVR	HIV	p24	I
ILGLNKIVRMY	HIV	p24	I
ILIEICGHK	HIV	Protease	I
ILKDPVHGV	HIV	RT	I
ILKEPVHGV	HIV	RT	I
ILKEPVHGVY	HIV	RT	I
ILKLAGRWPVK	HIV	Integrase	I
ILRIPVHGV	HIV	RT	I
ILRSCNTSV	HIV	gp160	I
ILVESPAVL	HIV	Rev	I

IPAETGQETA	HIV	Integrase	I
IPIHYCAPA	HIV	gp160	I
IPLGDAKLII	HIV	Vif	I
IPLGDARLVI	HIV	Vif	I
IPLTEEAEL	HIV	RT	I
IPRRIRQGA	HIV	gp160	I
IPRRIRQGF	HIV	gp160	I
IPRRIRQGL	HIV	gp160	I
IPRRRIRQGL	HIV	gp160	I
IPTRIRQGL	HIV	gp160	I
IPYNPQSQGVV	HIV	Integrase	I
IPYNPQSQGW	HIV	Integrase	I
IQQEFGIPY	HIV	Integrase	I
IQQEFGIPYNPQ	HIV	Integrase	I
IRDKVQKEY	HIV	gp160	I
IRLRPGGKK	HIV	p17	I
IRLRPGGKKK	HIV	p17	I
ISERILSTY	HIV	Rev	I
ISERILSTYL	HIV	Rev	I
ISGKAKGWF	HIV	Vif	I
ISKKAKGWF	HIV	Vif	I
ISLWDQSLK	HIV	gp160	I
ISPRTLNAW	HIV	p24	I
ISPRTLNAWV	HIV	p24	I
ISTERILSTY	HIV	Rev	I
ITKALGISY	HIV	Tat	I
ITKGLGISYGR	HIV	Tat	I
ITLWQRPLV	HIV	Protease	I
ITTESIVIW	HIV	RT	I
ITVGPGQVFY	HIV	gp160	I
IVGAETFYVDGAAN	HIV	RT	I
IVGAETFYVDGAAS	HIV	RT	I
IVLPEKDSW	HIV	RT	I
IVNRNRQGY	HIV	gp160	I
IVNRVRQGY	HIV	gp160	I
IVNRVRQGYSP	HIV	gp160	I
IVTDSQYAL	HIV	RT	I
IVTRIVELL	HIV	gp160	I
IVWASRELER	HIV	p17	I
IYETYGDTW	HIV	Vpr	I
IYIGPGRAF	HIV	gp160	I
IYKRWIILG	HIV	p24	I
IYKRWIILGL	HIV	p24	I
IYKRWIILGLNK	HIV	p24	I
IYQEPFKNL	HIV	RT	I
IYQEPFKNLK	HIV	RT	I
IYQYMDDLYV	HIV	RT	I
IYSQKRQDI	HIV	Nef	I
KAACWWAGI	HIV	Integrase	I
KAAFDLSFF	HIV	Nef	I
KAALDLSHF	HIV	Nef	I
KAALDLSHFL	HIV	Nef	I
KAAVDLSHF	HIV	Nef	I
KAAVDLSHFL	HIV	Nef	I
KAAVDLSMFL	HIV	Nef	I
KAFSPEVIMPF	HIV	p24	I
KAFSPEVIPMF	HIV	p24	I
KAHDPEVHNVW	HIV	gp160	I
KALGPAATL	HIV	p24	I
KAPRKKGCW	HIV	p2p7p1p6	I
KAPRKKGCWK	HIV	p2p7p1p6	I
KAVRIKLFLY	HIV	Rev	I
KAVRLIKFLY	HIV	Rev	I
KAVRRLIKFLY	HIV	Rev	I
KAYDTEVHNVW	HIV	gp160	I
KAYETEVHNVW	HIV	gp160	I
KCGKEGHQMK	HIV	p2p7p1p6	I
KDTINEEAAEWDRL	HIV	p24	I
KEKGGLEGL	HIV	Nef	I
KEKGGLEGLVYSQK	HIV	Nef	I
KELYPLASL	HIV	p2p7p1p6	I
KELYPLTSL	HIV	p2p7p1p6	I
KEPVHGVYY	HIV	RT	I

KETINEEAA	HIV	p24	I
KEVHNVWAT	HIV	gp160	I
KGAFDLSFF	HIV	Nef	I
KGALDLSHF	HIV	Nef	I
KGFNPEVIPMF	HIV	p24	I
KHPGSQPKTA	HIV	Tat	I
KIAIRSENISNNA	HIV	gp160	I
KIATESIVIW	HIV	p24	I
KIEEEQNKSK	HIV	p17	I
KIEELRQHL	HIV	RT	I
KIEELRQHLL	HIV	RT	I
KIIKDYGKQM	HIV	Integrase	I
KIKPPLPSV	HIV	Vif	I
KIKPPLPSVTK	HIV	Vif	I
KILYQSNPY	HIV	Rev	I
KIQNFRVYY	HIV	Integrase	I
KIQNFRVYYR	HIV	Integrase	I
KIRLRPGGK	HIV	p17	I
KIRLRPGGKK	HIV	p17	I
KIRLRPGGKKKYKL	HIV	p17	I
KIRWIIGLNK	HIV	p24	I
KITTESIVIW	HIV	RT	I
KIWPSYKGR	HIV	p2p7p1p6	I
KKQEILDLWVY	HIV	Nef	I
KLGKAGYVT	HIV	RT	I
KLGKAGYVV	HIV	RT	I
KLNWASQIY	HIV	RT	I
KLPIWKETW	HIV	RT	I
KLTEDRWNK	HIV	Vif	I
KLTPLCVSL	HIV	gp160	I
KLTPLCVTL	HIV	gp160	I
KLTSCNTSV	HIV	gp160	I
KLVDFRELNK	HIV	RT	I
KLVGKLNWA	HIV	RT	I
KLWVTVYYGV	HIV	gp160	I
KMIGGIGGF	HIV	Protease	I
KMIGGIGGFI	HIV	Protease	I
KMSFEPIPIH	HIV	gp160	I
KNCGGEFFYCNS	HIV	gp160	I
KNCSFNMTT	HIV	gp160	I
KPCVKLTPL	HIV	gp160	I
KPCVKLTPLC	HIV	gp160	I
KPPLPSVKKL	HIV	Vif	I
KPPLPSVTKL	HIV	Vif	I
KPVVSTQLLL	HIV	gp160	I
KQNPDIVIY	HIV	RT	I
KQNPDIVIYQY	HIV	RT	I
KRKGGIGGY	HIV	Integrase	I
KRQDILDLWIY	HIV	Nef	I
KRQDILDLWVY	HIV	Nef	I
KRQEILDLW	HIV	Nef	I
KRQEILDLWVY	HIV	Nef	I
KRWIIILGLNK	HIV	p24	I
KRWIILGGLNK	HIV	p24	I
KRWIILGLNK	HIV	p24	I
KRWIILGLNKIV	HIV	p24	I
KRWIILGNK	HIV	p24	I
KRWIIMGLHK	HIV	p24	I
KRWIIMGLNK	HIV	p24	I
KRWIIMGNK	HIV	p24	I
KRWILGLNKIV	HIV	p24	I
KRWILGLNKIVRMY	HIV	p24	I
KRWILLGLNK	HIV	p24	I
KRYMIKHLV	HIV	p17	I
KSALDLSHF	HIV	Nef	I
KTAVQMAVF	HIV	Integrase	I
KTGRLIKLLY	HIV	Rev	I
KTKPPLPSVKK	HIV	Vif	I
KTLPLCVTL	HIV	gp160	I
KTQQAAADK	HIV	p17	I
KTVKCFNCGR	HIV	p2p7p1p6	I
KTVRLIKFLY	HIV	Rev	I
KTVRLIKRLY	HIV	Rev	I

KVIEVAQGA	HIV	gp160	I
KVRQYDQIL	HIV	Protease	I
KVVPRRKAK	HIV	Integrase	I
KVYLAWVPA	HIV	RT	I
KVYLAWVPAHK	HIV	RT	I
KWILGLNKIVRMY	HIV	p24	I
KYCWNLLQY	HIV	gp160	I
KYKLKHIVW	HIV	p17	I
KYLGSLVQY	HIV	gp160	I
KYLWNLLQY	HIV	gp160	I
KYMLKHIVW	HIV	p17	I
KYRLKHIVW	HIV	p17	I
KYRLKHLVW	HIV	p17	I
KYTAFTIPSI	HIV	RT	I
KYWWNLLQY	HIV	gp160	I
LADQLIHLHY	HIV	Vif	I
LAFHHVAREL	HIV	Nef	I
LAIVALVVA	HIV	Vpu	I
LARNCRAPRK	HIV	p2p7p1p6	I
LEKHGAITS	HIV	Nef	I
LEWRFDSRL	HIV	Nef	I
LFCASCAKAY	HIV	gp160	I
LFCASDAKAY	HIV	gp160	I
LFLDGIDKA	HIV	RT-Integrase	I
LFNTVATLY	HIV	p17	I
LGLNKIVRMY	HIV	p24	I
LGLNKIVRMYS	HIV	p24	I
LGPGRVWYTT	HIV	gp160	I
LGVAPTTTKRRWER	HIV	gp160	I
LHPVHAGPI	HIV	p24	I
LKEPVHGVY	HIV	RT	I
LLDTGADDTVL	HIV	Protease	I
LLDTIAIAV	HIV	gp160	I
LLLYWGRELK	HIV	gp160	I
LLNATAIAV	HIV	gp160	I
LLNATDIAV	HIV	gp160	I
LLQLTVWGI	HIV	gp160	I
LLQYWSQEL	HIV	gp160	I
LLRAIEAQQHL	HIV	gp160	I
LLRWGLTTPDKK	HIV	RT	I
LLWKGEGAV	HIV	Integrase	I
LLWKGEGAW	HIV	Integrase	I
LPCRIKQFINMWQE	HIV	gp160	I
LPCRIKQII	HIV	gp160	I
LPCRIKWII	HIV	gp160	I
LPGRWKPKMI	HIV	Protease	I
LPLPPLDRL	HIV	Rev	I
LPPIVAKEI	HIV	Integrase	I
LPPLDRLTL	HIV	Rev	I
LPPLERLTL	HIV	Rev	I
LPPVVAKEI	HIV	Integrase	I
LPQGWKGSPA	HIV	RT	I
LPQGWKGSPAI	HIV	RT	I
LQLPPIERL	HIV	Rev	I
LQLPPLERL	HIV	Rev	I
LRAVRIIKI	HIV	Rev	I
LRWGFCTPDKK	HIV	RT	I
LSEGATPQDL	HIV	p24	I
LSHFLKEKGGLEG	HIV	Nef	I
LSPRTLNAW	HIV	p24	I
LTDTTNQKT	HIV	RT	I
LTEEAELEL	HIV	RT	I
LTFGWCFKL	HIV	Nef	I
LTFGWCFKLV	HIV	Nef	I
LTFGWCYKL	HIV	Nef	I
LTFGWCYKLV	HIV	Nef	I
LVGKLNWASQIY	HIV	RT	I
LVGPTPANI	HIV	Protease	I
LVGPTPVNI	HIV	Protease	I
LVGPTPVNII	HIV	Protease	I
LVILAIVALV	HIV	Vpu	I
LVQNSNPDCK	HIV	p24	I
LVWASRELERF	HIV	p17	I

LWVTVYYGV	HIV	gp160	I
LYNTVATLY	HIV	p17	I
LYPLASLRSL	HIV	p2p7p1p6	I
MASDFNLPPV	HIV	Integrase	I
MAVFIHNFK	HIV	Integrase	I
MGARASVLSG	HIV	p17	I
MGPKRAFYAT	HIV	gp160	I
MHEDIISLW	HIV	gp160	I
MKKGLGISYGR	HIV	Tat	I
MQMLKDTINEEAAE	HIV	p24	I
MTKGLGISY	HIV	Tat	I
MTKILEPFR	HIV	RT	I
MTNNPPIPV	HIV	p24	I
MTSNPPIPV	HIV	p24	I
MTYKAALDL	HIV	Nef	I
MTYKAAVDL	HIV	Nef	I
MVHQAISPR	HIV	p24	I
MVHQSMSPRTLNAW	HIV	p24	I
NAKTIIVHL	HIV	gp160	I
NANPDCKTI	HIV	p24	I
NANPDCKTILRAL	HIV	p24	I
NANPDSKTI	HIV	p24	I
NAWVKIEEK	HIV	p24	I
NCGGEFFYCN	HIV	gp160	I
NCSFNISTSI	HIV	gp160	I
NCYCKKCCF	HIV	Tat	I
NCYCKKCCY	HIV	Tat	I
NCYCKRCCF	HIV	Tat	I
NETPGIRYQY	HIV	RT	I
NFRGPKRIKCFNCG	HIV	p2p7p1p6	I
NIWATHACV	HIV	gp160	I
NLQEQIGWM	HIV	p24	I
NLQEQIGWMT	HIV	p24	I
NLWVTVYYGV	HIV	gp160	I
NMWQEVGKAM	HIV	gp160	I
NNETPGIRY	HIV	RT	I
NNETPGVRY	HIV	RT	I
NPDCKTILRAL	HIV	p24	I
NPDIVIYQY	HIV	RT	I
NPDIVIYQYM	HIV	RT	I
NPDVILIQY	HIV	RT	I
NPEIVIYQY	HIV	RT	I
NPVPVGNIY	HIV	p24	I
NRWQVMIVW	HIV	Vif	I
NSGGEFFYSNS	HIV	gp160	I
NSNPDCKTIL	HIV	p24	I
NSSKVSQNY	HIV	p17	I
NSSQVSQNY	HIV	p17	I
NSSQVSQNYP	HIV	p17-p24	I
NTMLNTVGGHQAAM	HIV	p24	I
NTPPLVKLW	HIV	RT	I
NTPVFAIKK	HIV	RT	I
NTPVFAIKKK	HIV	RT	I
NTVATLYCV	HIV	p17	I
NVTENFNMW	HIV	gp160	I
NVWATHACV	HIV	gp160	I
NWRSELYKY	HIV	gp160	I
NYTPGPGVRY	HIV	Nef	I
NYTPGPGVRYPLT	HIV	Nef	I
PDCKTILKA	HIV	p24	I
PFRDYVDRF	HIV	p24	I
PFRDYVDRFF	HIV	p24	I
PGGKKRYRLKHL	HIV	p17	I
PGMDGPKVKQ	HIV	RT	I
PIPVGDIYK	HIV	p24	I
PIQKETWEAW	HIV	RT	I
PIQKETWETW	HIV	RT	I
PIVLPEKDSW	HIV	RT	I
PKTACTNCY	HIV	Tat	I
PLDESFRKY	HIV	RT	I
PLTFGWCFKL	HIV	Nef	I
PLTFGWCYK	HIV	Nef	I
PLTFGWCYKL	HIV	Nef	I

PLTFGWCYKLV	HIV	Nef	I
PLTSLKSLFGS	HIV	p2p7p1p6	I
PLVKLWYQL	HIV	RT	I
PMFTALSEGAT	HIV	p24	I
PNCGGEFFY	HIV	gp160	I
PPIPVGDIH	HIV	p24	I
PPIPVGDIY	HIV	p24	I
PPIPVGEIY	HIV	p24	I
PPQGSQTHQV	HIV	Tat	I
PPSGKGGNY	HIV	p2p7p1p6	I
PPVPVGDIY	HIV	p24	I
PQDLNTMLN	HIV	p24	I
PQDLNTMLNTV	HIV	p24	I
PQVPLRPMTY	HIV	Nef	I
PSSGGDLEITTHSF	HIV	gp160	I
PTDPNPQEI	HIV	gp160	I
PTPVNIIGRNL	HIV	Protease	I
PTRRELQVW	HIV	Gag_Pol_TF	I
PTSRELQVW	HIV	Gag_Pol_TF	I
PTVLESGTKE	HIV	Rev	I
PVDPRLEPW	HIV	Tat	I
PVGDIYKRWII	HIV	p24	I
PVPFQLPPL	HIV	Rev	I
QAISPRTLNAV	HIV	p24	I
QAISPRTLNAW	HIV	p24	I
QASGEVKNW	HIV	p24	I
QASKEVKNWV	HIV	p24	I
QASQEVKNW	HIV	p24	I
QATQDVKNW	HIV	p24	I
QATQEVKGW	HIV	p24	I
QATQEVKNM	HIV	p24	I
QATQEVKNW	HIV	p24	I
QATWIPEWEF	HIV	RT	I
QDILDLWIY	HIV	Nef	I
QEEHEKYHSNW	HIV	Integrase	I
QEILDLWVY	HIV	Nef	I
QELKNSAVSL	HIV	gp160	I
QELLALDKW	HIV	gp160	I
QEPIDKELY	HIV	p2p7p1p6	I
QFRNKTIVF	HIV	gp160	I
QGVGGPGHKARVL	HIV	p24	I
QGWKGSPAI	HIV	RT	I
QGYFPDWQNY	HIV	Nef	I
QIIEQLIKK	HIV	RT	I
QILEQLQPA	HIV	p17	I
QILEQLQPAL	HIV	p17	I
QILGEPPTV	HIV	Rev	I
QIRSLSGWIL	HIV	Rev	I
QIRTYSGWI	HIV	Rev	I
QIYAGIKVK	HIV	RT	I
QIYPGIKVK	HIV	RT	I
QIYPGIKVR	HIV	RT	I
QIYQEPFKNLK	HIV	RT	I
QIYQEPFKNLKTG	HIV	RT	I
QLDCTHLEGK	HIV	Integrase	I
QLPPLERLT	HIV	Rev	I
QLQPSLQTGS	HIV	p17	I
QLTEAVQKI	HIV	RT	I
QMAVFIHNFK	HIV	Integrase	I
QMHEDIISL	HIV	gp160	I
QMQEDIISL	HIV	gp160	I
QMVHQAISPR	HIV	p24	I
QPDKSESELV	HIV	RT	I
QPIQIAIAAL	HIV	Vpu	I
QPKTACTTCY	HIV	Tat	I
QPKTPCNKCY	HIV	Tat	I
QRGWEVLKY	HIV	gp160	I
QRPLVTIKI	HIV	Protease	I
QRPLVTVKIG	HIV	Protease	I
QRQRQIQSL	HIV	Rev	I
QRRQDILDL	HIV	Nef	I
QTDPAVKNWM	HIV	p24	I
QTKGLGISY	HIV	Tat	I

QTRVLAIERYL	HIV	gp160	I
QVLGESPTVL	HIV	Rev	I
QVPLRPMTY	HIV	Nef	I
QVPLRPMTYK	HIV	Nef	I
QVPLRPMTYKA	HIV	Nef	I
QVPLRPMYTK	HIV	Nef	I
QVPLRRMTYK	HIV	Nef	I
QVRDQAEHL	HIV	Integrase	I
QVSQNYPIV	HIV	p17-p24	I
QYDDAVYKL	HIV	Protease	I
QYDQIPIEI	HIV	Protease	I
RAEPAADGV	HIV	Nef	I
RAEQASQEV	HIV	p24	I
RAEQASQEVK	HIV	p24	I
RAEQATQDVKN	HIV	p24	I
RAFSPEVIP	HIV	p24	I
RAIEAQQHL	HIV	gp160	I
RAIEAQQHM	HIV	gp160	I
RAIEAQQHML	HIV	gp160	I
RAIEAQQWQ	HIV	gp160	I
RAIEVQQHL	HIV	gp160	I
RALGPGATL	HIV	p24	I
RALGPGATL/M	HIV	p24	I
RAMASDFNL	HIV	Integrase	I
RAPRKKGCW	HIV	p2p7p1p6	I
RAVGMGALIFEFL	HIV	gp160	I
RAYRAILHI	HIV	gp160	I
RCSSNITGLL	HIV	gp160	I
RDYVDRFFKTL	HIV	p24	I
RDYVDRFYKTL	HIV	p24	I
REHLLKWGF	HIV	RT	I
REPHNEWTL	HIV	Vpr	I
REPYNEWTL	HIV	Vpr	I
RETKLGKAGY	HIV	RT	I
RFAVNPGLL	HIV	p17	I
RGPGRAFVTI	HIV	gp160	I
RGPGRAFYTT	HIV	gp160	I
RGRQKVVPL	HIV	RT	I
RIDVKDTKEAL	HIV	p17	I
RIGCRHSRIGV	HIV	Vpr	I
RIGPGQTFY	HIV	gp160	I
RIKPPLPSVTK	HIV	Vif	I
RIKQFINMW	HIV	gp160	I
RIKQIINMW	HIV	gp160	I
RILQQLLFI	HIV	Vpr	I
RILSTYLGR	HIV	Rev	I
RIRQGLERA	HIV	gp160	I
RIRTWKSLVK	HIV	Vif	I
RKAKIIKDY	HIV	Integrase	I
RKAKIIRDY	HIV	Integrase	I
RKNYQHLWK	HIV	gp160	I
RLAFHHMAR	HIV	Nef	I
RLAFHHVAR	HIV	Nef	I
RLAFHHVARELHPE	HIV	Nef	I
RLHPVHAGPIA	HIV	p24	I
RLISCNTSV	HIV	gp160	I
RLLNAWVKV	HIV	p24	I
RLRDLLLIV	HIV	gp160	I
RLRDLLLIVTR	HIV	gp160	I
RLRPGGKKHY	HIV	p17	I
RLRPGGKKHYM	HIV	p17	I
RLRPGGKKK	HIV	p17	I
RLRPGGKKKK	HIV	p17	I
RLRPGGKKKKY	HIV	p17	I
RLRPGGKKKY	HIV	p17	I
RLRPGGKKKYK	HIV	p17	I
RLRPGGKKRYRL	HIV	p17	I
RLRQGGKKK	HIV	p17	I
RLSYNTVATLY	HIV	p17	I
RLVNGSLAL	HIV	gp160	I
RLVSGFLAL	HIV	gp160	I
RMRGAHTNDV	HIV	RT	I
RMRGAHTNDVK	HIV	RT	I

RMRRAEPAA	HIV	Nef	I
RMYSPTSIL	HIV	p24	I
RMYSPVSIL	HIV	p24	I
RPAELVLQL	HIV	Rev	I
RPAEPVPLQL	HIV	Rev	I
RPEPTAPPA	HIV	p2p7p1p6	I
RPGGKKKKYK	HIV	p17	I
RPGGKKKYKL	HIV	p17	I
RPGGKKKYML	HIV	p17	I
RPGGKKRYKL	HIV	p17	I
RPGGKKRYM	HIV	p17	I
RPIVSTQLL	HIV	gp160	I
RPMTYKAAL	HIV	Nef	I
RPMTYKAAV	HIV	Nef	I
RPMTYKGAL	HIV	Nef	I
RPNNNTRKS	HIV	gp160	I
RPNNNTRKSI	HIV	gp160	I
RPNNNTRRGI	HIV	gp160	I
RPQQVPLRPM	HIV	Nef	I
RPQVPLRPM	HIV	Nef	I
RPQVPLRPMTY	HIV	Nef	I
RPRPGGKKK	HIV	p17	I
RPVVSTQLL	HIV	gp160	I
RPVVSTQLLL	HIV	gp160	I
RPYNNTRQS	HIV	gp160	I
RQANFLGKI	HIV	p2p7p1p6	I
RQDILDLWI	HIV	Nef	I
RQDILDLWIY	HIV	Nef	I
RQDILDLWV	HIV	Nef	I
RQDILDLWVY	HIV	Nef	I
RQGLERALL	HIV	gp160	I
RQYDQILIEI	HIV	Protease	I
RQYDQIPIEI	HIV	Protease	I
RRGWEVLKY	HIV	gp160	I
RRIRQGLERILL	HIV	gp160	I
RRKPPLPSIAK	HIV	Vif	I
RRQDILDLWI	HIV	Nef	I
RRQDILDLWIY	HIV	Nef	I
RRQDILDLWVY	HIV	Nef	I
RRWIQLGLQK	HIV	p24	I
RSAEPVPLQL	HIV	Rev	I
RSIRLVSGFL	HIV	gp160	I
RSLFNTVATLY	HIV	p17	I
RSLYNTATLY	HIV	p17	I
RSLYNTVATLY	HIV	p17	I
RSLYNTVAVLY	HIV	p17	I
RTLNAWVKV	HIV	p24	I
RTRGAHTNDVK	HIV	RT	I
RTVRLIKLLY	HIV	Rev	I
RVIEVAQRV	HIV	gp160	I
RVIEVLQRA	HIV	gp160	I
RVKEKYQHL	HIV	gp160	I
RVLAEAMSQV	HIV	p24-p2p7p1p6	I
RVLKQVTEK	HIV	gp160	I
RVYLSWVPAHK	HIV	RT	I
RWIILGLNK	HIV	p24	I
RYLKDQQLL	HIV	gp160	I
RYLRDQQLL	HIV	gp160	I
RYLRDQQLLGI	HIV	gp160	I
RYPLTFGWCF	HIV	Nef	I
RYPLTFGWCFY	HIV	Nef	I
RYPLTFGWCY	HIV	Nef	I
RYPLTFGWCYK	HIV	Nef	I
SAAVKAACWW	HIV	Integrase	I
SAEPVPLQL	HIV	Rev	I
SEGATPQDL	HIV	p24	I
SEGCRQILG	HIV	p17	I
SEVNIVTDSQY	HIV	RT	I
SEWILSTHL	HIV	Rev	I
SFDPIPIHY	HIV	gp160	I
SFEPIPIHY	HIV	gp160	I
SFNCGGEFF	HIV	gp160	I
SFNCGGEFFY	HIV	gp160	I

SFNCRGEFFY	HIV	gp160	I
SFTCGGEFF	HIV	gp160	I
SHFLKEKGGLEGL	HIV	Nef	I
SIFQSSMTK	HIV	RT	I
SILDIRQGPK	HIV	p24	I
SKRIVKCFNCG	HIV	p2p7p1p6	I
SLAFRHVAR	HIV	Nef	I
SLFNTVATL	HIV	p17	I
SLFNTVATLY	HIV	p17	I
SLGQHIYET	HIV	Vpr	I
SLLNATAIAV	HIV	gp160	I
SLLNATDIAV	HIV	gp160	I
SLVKHHMYI	HIV	Vif	I
SLVKHHMYV	HIV	Vif	I
SLWDQSLKP	HIV	gp160	I
SLYNIVATLWCVH	HIV	p17	I
SLYNTAVTL	HIV	p17	I
SLYNTIATL	HIV	p17	I
SLYNTVAAL	HIV	p17	I
SLYNTVATL	HIV	p17	I
SLYNTVATLY	HIV	p17	I
SLYNTVAVL	HIV	p17	I
SPAIFQSSM	HIV	RT	I
SPAIFQSSMT	HIV	RT	I
SPHPRISSEV	HIV	Vif	I
SPIETVPVKL	HIV	RT	I
SPRTLNAWV	HIV	p24	I
SPRTLNAWVKV	HIV	p24	I
SPSIFQSSM	HIV	RT	I
SQKQEQIDK	HIV	p2p7p1p6	I
SQVTNSATI	HIV	p2p7p1p6	I
SRAKWNNTL	HIV	gp160	I
SRKAKGWFY	HIV	Vif	I
STGIRRVLFL	HIV	RT-Integrase	I
STLQEQIAW	HIV	p24	I
STLQEQIGW	HIV	p24	I
STLQEQIGWM	HIV	p24	I
STTVKAACW	HIV	Integrase	I
STTVKAACWW	HIV	Integrase	I
STWNVNGTW	HIV	gp160	I
SVITQACPK	HIV	gp160	I
SVLSGGQLDR	HIV	p17	I
SVPLDESFRK	HIV	RT	I
SVPLRPMTYK	HIV	Nef	I
SYHRLRDLLLIVTR	HIV	gp160	I
SYRRLRDLL	HIV	gp160	I
SYVDRFYKSL	HIV	p24	I
TACNKCHCK	HIV	Tat	I
TACNNCYCK	HIV	Tat	I
TACNNCYCKK	HIV	Tat	I
TAVPWNASW	HIV	gp160	I
TAVPWNSSW	HIV	gp160	I
TELQAIQLAL	HIV	RT	I
THLEGKIIL	HIV	Integrase	I
THLEGKVIL	HIV	Integrase	I
TIKIGGQLK	HIV	Protease	I
TINEEAAEW	HIV	p24	I
TKALGISYGR	HIV	Tat	I
TKIQNFRVYY	HIV	Integrase	I
TLEILEELKN	HIV	Vpr	I
TLFCASDAK	HIV	gp160	I
TLGPGRVLY	HIV	gp160	I
TLNAWVKLV	HIV	p24	I
TLNAWVKVI	HIV	p24	I
TLNAWVKVV	HIV	p24	I
TLNFPISPI	HIV	Protease-RT	I
TLQEQIGWM	HIV	p24	I
TLRAEQATQD	HIV	p24	I
TLSQIVTKL	HIV	gp160	I
TLTSCNTSV	HIV	gp160	I
TLYCVHQKI	HIV	p17	I
TLYCVHQRI	HIV	p17	I
TMGAASITL	HIV	gp160	I

TNSANIMMQR	HIV	p2p7p1p6	I
TNVPWNSSW	HIV	gp160	I
TPGPGIRYPL	HIV	Nef	I
TPGPGRFPL	HIV	Nef	I
TPGPGTRYPL	HIV	Nef	I
TPGPGVKYPL	HIV	Nef	I
TPGPGVRYP	HIV	Nef	I
TPGPGVRYPL	HIV	Nef	I
TPKKIKPPL	HIV	Vif	I
TPPLVKLWYQL	HIV	RT	I
TPQDLNMML	HIV	p24	I
TPQDLNMMLN	HIV	p24	I
TPQDLNQML	HIV	p24	I
TPQDLNQMLNTV	HIV	p24	I
TPQDLNTML	HIV	p24	I
TPQDLNTMLN	HIV	p24	I
TPQDLNTMLNT	HIV	p24	I
TPQVPLRPM	HIV	Nef	I
TPQVPLRPMTY	HIV	Nef	I
TPSQKQEPI	HIV	p2p7p1p6	I
TPVNIIGRNML	HIV	Protease	I
TPYDINQML	HIV	p24	I
TQGYFPDWQNY	HIV	Nef	I
TQGYFPDWQNYT	HIV	Nef	I
TQICGTLNF	HIV	Protease	I
TQIGCTLNF	HIV	Protease	I
TQMNWPNLWK	HIV	gp160	I
TRANSPTRR	HIV	Gag_Pol_TF	I
TRYPLTFGW	HIV	Nef	I
TSTLQEQIAW	HIV	p24	I
TSTLQEQIGN	HIV	p24	I
TSTLQEQIGW	HIV	p24	I
TSTLQEQIGWF	HIV	p24	I
TSTLQEQVGW	HIV	p24	I
TSTLQRQIGW	HIV	p24	I
TSTPQEQIGW	HIV	p24	I
TSTVEEQIQW	HIV	p24	I
TSTVEEQQIW	HIV	p24	I
TTLFCASDAK	HIV	gp160	I
TTPDKKHQKE	HIV	RT	I
TTVPWNVSW	HIV	gp160	I
TVLDVGDAY	HIV	RT	I
TVLDVGDIY	HIV	RT	I
TVRERMRRA	HIV	Nef	I
TVRLIKFLY	HIV	Rev	I
TVYYGVPVW	HIV	gp160	I
TVYYGVPVWK	HIV	gp160	I
TWAGVEAIIRI	HIV	Vpr	I
TWAVEAIIRI	HIV	Vpr	I
TWETWWTEYW	HIV	RT	I
TYNETYNEI	HIV	gp160	I
TYQIYQEPF	HIV	RT	I
VAEGTDRVIEI	HIV	gp160	I
VASCDKCQL	HIV	Integrase	I
VCFITKALGI	HIV	Tat	I
VCFMTKGLGI	HIV	Tat	I
VCFTTKGLGI	HIV	Tat	I
VDRFYKLTRAEQAS	HIV	p24	I
VDRFYKTLRAEQAS	HIV	p24	I
VERYLKDQQL	HIV	gp160	I
VEWPAVRERM	HIV	Nef	I
VFAVLSIVNR	HIV	gp160	I
VGEIYKRWIIGLNK	HIV	p24	I
VGFPVRPQV	HIV	Nef	I
VGFPVTPQVPLRMT	HIV	Nef	I
VGGPGHKARVL	HIV	p24	I
VHPVHAGPIA	HIV	p24	I
VHQAISPRTL	HIV	p24	I
VINRVRQGY	HIV	gp160	I
VIVQYMDDL	HIV	RT	I
VIYQYMDDL	HIV	RT	I
VIYQYMMDL	HIV	RT	I
VIYQYNDDL	HIV	RT	I

VKNWMTETL	HIV	p24	I
VKNWMTETLL	HIV	p24	I
VKNWMTETLLV	HIV	p24	I
VKVIEEKAF	HIV	p24	I
VKVVEEKAF	HIV	p24	I
VLAEAMSQA	HIV	p24-p2p7p1p6	I
VLAEAMSQV	HIV	p24-p2p7p1p6	I
VLAEAMSQVT	HIV	p24-p2p7p1p6	I
VLALERYLKDQQL	HIV	gp160	I
VLDVGDAYFSV	HIV	RT	I
VLEWRFDSRL	HIV	Nef	I
VLMWKFDSRL	HIV	Nef	I
VLVGPTPVNI	HIV	Protease	I
VLYCVHQRI	HIV	p17	I
VNGFLALAW	HIV	gp160	I
VNIVTDSQYA	HIV	RT	I
VPLDEDFRKY	HIV	RT	I
VPLDEGFRKY	HIV	RT	I
VPLDKDFRKY	HIV	RT	I
VPLRPMTYK	HIV	Nef	I
VPLTREAEL	HIV	RT	I
VPRRKAKII	HIV	Integrase	I
VPRRKVKII	HIV	Integrase	I
VPTDPNPPEV	HIV	gp160	I
VPVEPEKVEEA	HIV	Nef	I
VPVWKEAKTTL	HIV	gp160	I
VPVWKEATT	HIV	gp160	I
VPVWKEATTT	HIV	gp160	I
VPVWKEATTTL	HIV	gp160	I
VQHAISPRTLNAWV	HIV	p24	I
VQNANPDCK	HIV	p24	I
VQNLQGQMV	HIV	p24	I
VQNSNPDCK	HIV	p24	I
VQRTCRAIL	HIV	gp160	I
VQYWGLELK	HIV	gp160	I
VRHFPRIWL	HIV	Vpr	I
VRMYSPVSI	HIV	p24	I
VRQYDQIPIEI	HIV	Protease	I
VRYPLTFGW	HIV	Nef	I
VSFEPIPIH	HIV	gp160	I
VSFEPIPIHY	HIV	gp160	I
VSFEPIPPHYCA	HIV	gp160	I
VSGFLALAW	HIV	gp160	I
VSKKAKGWI	HIV	Vif	I
VTDSQYALGI	HIV	RT	I
VTEEFNMWK	HIV	gp160	I
VTENFNMWK	HIV	gp160	I
VTENFNMWKN	HIV	gp160	I
VTIKIGGQLK	HIV	Protease	I
VTILIGGQLK	HIV	Protease	I
VTKLTEDRW	HIV	Vif	I
VTVYYGVPV	HIV	gp160	I
VTVYYGVPVWK	HIV	gp160	I
VTVYYGVPVWR	HIV	gp160	I
VVAAIIAIV	HIV	Vpu	I
VWKDAETTLF	HIV	gp160	I
VWKEAKTTLF	HIV	gp160	I
VWKEATTTLF	HIV	gp160	I
VYHTQGYFPDWQNY	HIV	Nef	I
VYYDPSKDL	HIV	RT	I
VYYGVPVWKEA	HIV	gp160	I
WASRELERF	HIV	p17	I
WETWWMDYW	HIV	RT	I
WHLGHGVSI	HIV	Vif	I
WHLGHVSI	HIV	Vif	I
WHLGQGVSI	HIV	Vif	I
WIYHTQGYF	HIV	Nef	I
WIYHTQGYFPDWQ	HIV	Nef	I
WKFDSRLAF	HIV	Nef	I
WKGPAKLLW	HIV	Integrase	I
WKGSPAIFQSSMT	HIV	RT	I
WKPKMIGGI	HIV	Protease	I
WLWYIKIFI	HIV	gp160	I

WLWYIRIFI	HIV	gp160	I
WMTNNPPIPV	HIV	p24	I
WPVDPRLEPW	HIV	Tat	I
WQEVGKAMY	HIV	gp160	I
WRFDSRLAF	HIV	Nef	I
WRFDSRLAFH	HIV	Nef	I
WRFDSRLAHH	HIV	Nef	I
WSKSSIIGW	HIV	Nef	I
WYIKIFIII	HIV	gp160	I
WYIKIFIMI	HIV	gp160	I
WYIRIFIMI	HIV	gp160	I
YCAPAGFAIL	HIV	gp160	I
YCVHAGIEVRD	HIV	p17	I
YETEVHNVW	HIV	gp160	I
YFPDWQDYT	HIV	Nef	I
YFPDWQNYT	HIV	p24	I
YHTQGYFPDW	HIV	Nef	I
YHTQGYFPDWQ	HIV	Nef	I
YIEAEVIPA	HIV	Integrase	I
YIKIFIMIV	HIV	gp160	I
YKGALDLSHFL	HIV	Nef	I
YKVVKIEPL	HIV	gp160	I
YLAWVPAHK	HIV	RT	I
YLGGSEEPV	HIV	Rev	I
YLPTFGWCY	HIV	Nef	I
YLRDQQLLGIWGC	HIV	gp160	I
YNIVATLWCVHQ	HIV	p17	I
YPGIKVKQL	HIV	RT	I
YPGIKVRQL	HIV	RT	I
YPIGKVRQL	HIV	RT	I
YPLASLRSL	HIV	p2p7p1p6	I
YPLASLRSLF	HIV	p2p7p1p6	I
YPLTFGWCF	HIV	Nef	I
YPLTFGWCY	HIV	Nef	I
YPLTSLRSL	HIV	p2p7p1p6	I
YPLTSLRSLF	HIV	p2p7p1p6	I
YQYMDDLYV	HIV	RT	I
YRLGVGALI	HIV	Vpu	I
YRLINCNTSV	HIV	gp160	I
YSENSSEYY	HIV	gp160	I
YSPLSLQTL	HIV	gp160	I
YTAFTIPSI	HIV	RT	I
YTAFTIPSV	HIV	RT	I
YTPGPGIRY	HIV	Nef	I
YTPGPGTRY	HIV	Nef	I
YTPGPGVRY	HIV	Nef	I
YVDRFFKAL	HIV	p24	I
YVDRFFKRL	HIV	p24	I
YVDRFFKTL	HIV	p24	I
YVDRFYKTL	HIV	p24	I
AIRHIPRRIRQGLER	HIV	gp160	II
APPIGGQISCSSNITY	HIV	gp160	II
ASLWNWFNITNWLWY	HIV	gp160	II
AVLSIVNRVRQGYSPLSF	HIV	gp160	II
CNISRAQWNNTLEQI	HIV	gp160	II
CRIKQIINMWQEVGR	HIV	gp160	II
CSGKLICTTAVP	HIV	gp160	II
CTTAVPWNASWS	HIV	gp160	II
CVKLTPLCVTLECRN	HIV	gp160	II
CVPTDPNPQEVV	HIV	gp160	II
DMRDNWRSELYKYKV	HIV	gp160	II
DRVIEVVGQAYRAIR	HIV	gp160	II
DRVIEVVQGAYRAIR	HIV	gp160	II
EESQNQQEKNEQELL	HIV	gp160	II
EFFYCNTTQLFNNTW	HIV	gp160	II
EIDNYTNTIYTLLEEC	HIV	gp160	II
ELYKYKVVEIKPLGV	HIV	gp160	II
EMVNQMQEDVISLWD	HIV	gp160	II
ENFNMWKNEMVNQMQ	HIV	gp160	II
ENVTENFNMWKNEMV	HIV	gp160	II
EQIWNHTTWMEWDRE	HIV	gp160	II
EQRGPGRAFVTIGKI	HIV	gp160	II
ETFRPGGGDMRNNWR	HIV	gp160	II

EVVIRSANFTDNAKT	HIV	gp160	II
FAILKCNNK	HIV	gp160	II
FDPIPIHYCTPAGYA	HIV	gp160	II
FINMWQEVGKAMYAPPI	HIV	gp160	II
FNNTWRLNHTEGTKGC	HIV	gp160	II
FRPGGGDMRDNWRSEL	HIV	gp160	II
FYRLDIVPLTKNYS	HIV	gp160	II
GATTTLFCASDAKAY	HIV	gp160	II
GIEEEGGERDRDR	HIV	gp160	II
GIRPIVSTQLLLNGSC	HIV	gp160	II
GIVQQQNNLLRAIEA	HIV	gp160	II
GIWGCSGKLI	HIV	gp160	II
GIWGCSGKLIC	HIV	gp160	II
GNSNNESEIFRPGGG	HIV	gp160	II
GRAFVTIGKIGNMRQ	HIV	gp160	II
GSNNTVGNPIILPCRI	HIV	gp160	II
HEDIISLWDQSLK	HIV	gp160	II
HIPRRIRQGLERILL	HIV	gp160	II
IGGQIRCSSN	HIV	gp160	II
IISLWDQSLKPC	HIV	gp160	II
IKLFIMIVGGLVGLR	HIV	gp160	II
INCNTSAITQACPKV	HIV	gp160	II
INMWQEVGKAMYAPP	HIV	gp160	II
IQRGPGRAFVTIGKIGN	HIV	gp160	II
ITIPCRIKQIINMWQ	HIV	gp160	II
KNCSFNISTSIRGKV	HIV	gp160	II
KQFINMWQEWGKAMYA	HIV	gp160	II
KYKVIKIEPLGIAPTC	HIV	gp160	II
LELDKWASLWNWFNITN	HIV	gp160	II
LGIWGCSGKLIC	HIV	gp160	II
LPCRIKQIINMWQEVY	HIV	gp160	II
MWQEVGKAMYAPPIGC	HIV	gp160	II
MYAPPIKGNITCKSN	HIV	gp160	II
NAKTIIVQLNESVAIC	HIV	gp160	II
NATTVVRDRKQTVYA	HIV	gp160	II
NCSFNATTVVRDRDQ	HIV	gp160	II
NESVAINCT	HIV	gp160	II
NKTFNGKGPCTNVSTY	HIV	gp160	II
PAGFAILKCNNKTFN	HIV	gp160	II
PAGFAILKCNNKTFNY	HIV	gp160	II
PEIVTHSFNCGGEFF	HIV	gp160	II
PIHYCAPAGFAILK	HIV	gp160	II
PIKGNITCKSNITGL	HIV	gp160	II
PKVTFDPIPIHYCTP	HIV	gp160	II
PPISGQIRCS	HIV	gp160	II
QIVKKLREQFGNNK	HIV	gp160	II
QNQQEKNEQELLE	HIV	gp160	II
QQHLLQLTVWGIKQL	HIV	gp160	II
QSSGGDPEIV	HIV	gp160	II
RDGGTNVTNDTEVFRC	HIV	gp160	II
RIHIGPGRAFYTTKN	HIV	gp160	II
RIIGDIRKAHCNISRY	HIV	gp160	II
RIQRGPGRAFVTIGK	HIV	gp160	II
RPVVSTQLLLNGSLA	HIV	gp160	II
SANFTDNAKTIIVQL	HIV	gp160	II
SFEPIPIHYCAP	HIV	gp160	II
SLKPCVKLTPLC	HIV	gp160	II
SLKPCVKLTPLCVSL	HIV	gp160	II
SLWDQSLKPCVKLTPL	HIV	gp160	II
SSGGKPEIVTHSFNC	HIV	gp160	II
SSNITGLLLTRDGGTC	HIV	gp160	II
SSSIITIPCRIKQII	HIV	gp160	II
SVITQACSKVSFE	HIV	gp160	II
SVVEINCTRPNNNTRKS	HIV	gp160	II
TEKLWVTVYYGVPVW	HIV	gp160	II
TGDIIGDIRQAHCNI	HIV	gp160	II
THACVPADPNPQEMV	HIV	gp160	II
TKAKRRVVEREKR	HIV	gp160	II
TLEQIVKKLREQFGNC	HIV	gp160	II
TNVSTVQCTHGRPIY	HIV	gp160	II
TQLFNSTWFNSTWST	HIV	gp160	II
TTLFCASKAKAYDTE	HIV	gp160	II
TWFNSTWSTKGSNNT	HIV	gp160	II

TWSTKGSNNTEGSDT	HIV	gp160	II
VGLNWVTVYYGVPVWK	HIV	gp160	II
VGRAMYAPPIKGNIT	HIV	gp160	II
VITQACPKVSFEPIP	HIV	gp160	II
VVIRSDNFTNNAKTIC	HIV	gp160	II
VYALFYRLDIVPLTK	HIV	gp160	II
VYYGVPVWKEA	HIV	gp160	II
WLNATAIAVTEGTDRC	HIV	gp160	II
WSNKSLEDIWDNMTWC	HIV	gp160	II
WVTVYYGVPVWKGAT	HIV	gp160	II
YFNMWKNNMV	HIV	gp160	II
YKVVEIKPLGVAPTT	HIV	gp160	II
YKYKVVKIEPLGVAP	HIV	gp160	II
YLRDQQLLGIWG	HIV	gp160	II
YVAEGTDRVIEVVQGAC	HIV	gp160	II
AGERIVDIIATDIQT	HIV	Integrase	II
ELKKIIGQVRDQAEHLK	HIV	Integrase	II
HSNWRAMASDFNLPP	HIV	Integrase	II
KQITKIQNFRVYY	HIV	Integrase	II
KTAVQMAVFFIHNFKR	HIV	Integrase	II
KTAVQMAVFIHNFKR	HIV	Integrase	II
LKTAVQMAVFIHNFK	HIV	Integrase	II
LWKGEGAVVIQDNSDIK	HIV	Integrase	II
QKQITKIQNFRVYYR	HIV	Integrase	II
RKGGIGGYSAGERIVDII	HIV	Integrase	II
SAGERIVDIIATDIQTK	HIV	Integrase	II
TEKLQKQITKIQNFRVYY	HIV	Integrase	II
VIQDNSDIKVVPRRKAKI	HIV	Integrase	II
AFHHMARELHPEYYKD	HIV	Nef	II
AWLEAQEEEEVGF	HIV	Nef	II
EEVGFPVRPQ	HIV	Nef	II
EVGFPVIPQVPLRPM	HIV	Nef	II
FDSRLAFHHVARELHP	HIV	Nef	II
FHHMARELH	HIV	Nef	II
FKGAFDLSFFLKEKGGL	HIV	Nef	II
KFDSRLAFHHMARELH	HIV	Nef	II
LAFHHVARELHPEYF	HIV	Nef	II
LEKHGAITSSNTAAT	HIV	Nef	II
LWVYHTGQGYFPDWQN	HIV	Nef	II
LWVYHTQGYFPDWQNY	HIV	Nef	II
NSLLHPMSLHGMDDPEK	HIV	Nef	II
PAVRERMRRAEPAADGV	HIV	Nef	II
PEKEVLVWKFDSRLAFH	HIV	Nef	II
QKRQDILDLWVYHTQGY	HIV	Nef	II
QVPLRPMTYKAAVDLSH	HIV	Nef	II
RRAEPAADGVGAVSRDL	HIV	Nef	II
RSVVGWPAVRERMRRA	HIV	Nef	II
SAIRERMRR	HIV	Nef	II
SSNTAATNAACAWLE	HIV	Nef	II
YKAAVDLSHFLKEKGGL	HIV	Nef	II
AAADTGHSSQVSQNY	HIV	p17	II
ASILRGGKLDKW	HIV	p17	II
ASRELERFALNPGLL	HIV	p17	II
ASRELERFAVNPGLL	HIV	p17	II
EELRSLYNTVATLYC	HIV	p17	II
EGCRQILGQLQPSLQTGS	HIV	p17	II
EIKDTKEALDKIEEE	HIV	p17	II
EKIRLRPGGKKKYKL	HIV	p17	II
EKIRLRPGGKKKYKLHK	HIV	p17	II
EKIRLRPGGKKKYKLKH	HIV	p17	II
ERFALNPGLLETSEGGK	HIV	p17	II
ERFALNPSLLETAEG	HIV	p17	II
ERFAVNPGLL	HIV	p17	II
ERFAVNPGLLETSEGCR	HIV	p17	II
GGKLDAWEKIRLRPG	HIV	p17	II
GKKHYMLKHLVWASRE	HIV	p17	II
GKKKYKLKHIVWASREL	HIV	p17	II
GLLETSEGCRQILGQL	HIV	p17	II
HIVWASRELERFAVN	HIV	p17	II
HYMLKHLVWAS	HIV	p17	II
KHIVWASRELERFAV	HIV	p17	II
LERFAVNPGLL	HIV	p17	II
LERFAVNPGLLETSE	HIV	p17	II

LKSLFNTVATLYCVH	HIV	p17	II
LRPGGKKKYKLKHIV	HIV	p17	II
MGARASVLSGGELDRWI	HIV	p17	II
PGLLETSEGCK	HIV	p17	II
RELERFAVN	HIV	p17	II
RLRPGGKKHYM	HIV	p17	II
SGGELDRWEKIRLRPGGI	HIV	p17	II
SLYNTVATLYCVHQR	HIV	p17	II
SLYNTVATLYCVHQRIEV	HIV	p17	II
SRELERFALNPSLLEE	HIV	p17	II
TGSEELRSLYNTVATLY	HIV	p17	II
TSEELKSLFVTVATL	HIV	p17	II
VATLYCVHAGI	HIV	p17	II
YKLKHIVWASRELER	HIV	p17	II
SQVSQNYPIVQNLQGQM	HIV	p17-p24	II
AAEWDRLHPVHAGPIA	HIV	p24	II
AFSPEVIPMFSALSEC	HIV	p24	II
AFSPEVIPMFSALSEGA	HIV	p24	II
AFSPEVIPMFSALSEGAT	HIV	p24	II
AFSPEVIPMFT	HIV	p24	II
AFSPEVIPMFTALSEGA	HIV	p24	II
AMQMKLETINEEAAE	HIV	p24	II
AMQMLKETINEEAAE	HIV	p24	II
AWVKVIEEKAFSPEV	HIV	p24	II
DLNTMLNTYGGHQAAC	HIV	p24	II
DRFFKTLRAEQAT	HIV	p24	II
DRFFKTLRAEQATQE	HIV	p24	II
DVKNWMTDTLLQNA	HIV	p24	II
DYVDRFYKTLRAE	HIV	p24	II
DYVDRFYKTLRAEQA	HIV	p24	II
EAAEWDRVHP	HIV	p24	II
EAAEWDRVHPVHAGP	HIV	p24	II
EEKAFSPEV	HIV	p24	II
EEKAFSPEVIP	HIV	p24	II
EIYKRWIILG	HIV	p24	II
EIYRWIILG	HIV	p24	II
EPRGSDIAGT	HIV	p24	II
EQIAWMTSNPPVPVG	HIV	p24	II
ETINEEAAEWDRVHPC	HIV	p24	II
ETINEEAAEWDRVHPVH	HIV	p24	II
EVIPMFSALS	HIV	p24	II
EWDRVHPVHA	HIV	p24	II
FFKTLRAEQATQE	HIV	p24	II
FRDYVDRFFKT	HIV	p24	II
FRDYVDRFYKTLRAE	HIV	p24	II
FYKTLRAEQASQ	HIV	p24	II
FYKTLRAEQASQE	HIV	p24	II
GEIYKRWIILGLNKI	HIV	p24	II
GEIYRWIILGLNKI	HIV	p24	II
GGHQAAMQMLKDTINEI	HIV	p24	II
GLNKIVRMYSPTSIL	HIV	p24	II
GPKEPFRDYVDRFYK	HIV	p24	II
GPKEPFRDYVDRFYKTLI	HIV	p24	II
GQMREPRGSDI	HIV	p24	II
GSDIAGTTSTLQEQI	HIV	p24	II
GSDIAGTTSTLQEQIC	HIV	p24	II
GTTSTLQEQIA	HIV	p24	II
IAPGQMREPRGSDIA	HIV	p24	II
ILGLNKIVRMY	HIV	p24	II
ILKALGPAATLEEMM	HIV	p24	II
INEEAAEWDRL	HIV	p24	II
IRQGPKEPFRDYVDR	HIV	p24	II
IVRMYSPTSILDIRQC	HIV	p24	II
IVRMYSPTSILDIRQGPK	HIV	p24	II
IYKRWIILGLNKIVR	HIV	p24	II
KIVRMYSPT	HIV	p24	II
KIVRMYSPTS	HIV	p24	II
KRWIILGLNKIVRMY	HIV	p24	II
KVVEEKAFSPEVIPM	HIV	p24	II
LDIRQGPKEPFRDYVC	HIV	p24	II
LGPAATLEEMMTACQ	HIV	p24	II
LHPVHAGPIAPGQMREPI	HIV	p24	II
LLVQNANPDCKTILR	HIV	p24	II

LNKIVRMYSPVSILD	HIV	p24	II
LRAEQASQEVKNWMTE	HIV	p24	II
MREPRGSKIAGTTST	HIV	p24	II
MTDTLLQNANPDCKTIL	HIV	p24	II
MTETLLVQNANPDCKTI	HIV	p24	II
NANPDCKTILKALGPAA	HIV	p24	II
NAWVKVVEEKAFSPEC	HIV	p24	II
NPPIPVGEIYKRWIIC	HIV	p24	II
NTMLNTVGGHQAAM	HIV	p24	II
PEVIPMFSALSEGATP	HIV	p24	II
PGQMREPRGSDIAGT	HIV	p24	II
PIVQNIQGQ	HIV	p24	II
PIVQNIQGQMVHQAI	HIV	p24	II
PIVQNLQGQMV	HIV	p24	II
PKEPFRDYV	HIV	p24	II
PMFTALSEGAT	HIV	p24	II
PQDLNMMLNIVGGHQA	HIV	p24	II
PQDLNTMLNTVGGHQ	HIV	p24	II
PRTLNAWVKVVEEKAF	HIV	p24	II
PVGDIYKRWIILGLNKIV	HIV	p24	II
PVGEIYKRWIILGLN	HIV	p24	II
PVGEIYKRWIILGLNKIV	HIV	p24	II
PVHGPIAPGQMREP	HIV	p24	II
QGQMVHQAISPRTLN	HIV	p24	II
QMVHQAISPRTLNAWV	HIV	p24	II
RAEQASQEVKNWMTE	HIV	p24	II
REPRGSDIAGTTSTL	HIV	p24	II
RFYKTLRAEQAS	HIV	p24	II
RLHPVHAGPIA	HIV	p24	II
SALSEGATPQDLNTMC	HIV	p24	II
SILDIRQGPKEPFRDYV	HIV	p24	II
SPTSILDIRQGPKEP	HIV	p24	II
STLQEQIGWMTNNPP	HIV	p24	II
STLQEQIGWMTNNPPIP	HIV	p24	II
TNNPPIPBGEIYKRW	HIV	p24	II
TPQDLNTMLNTVGGH	HIV	p24	II
VDRFYKTLRAEQASQ	HIV	p24	II
VHAGPIAPG	HIV	p24	II
VHQAISPRTLNAWVKC	HIV	p24	II
VKNWMTETLLVQNANC	HIV	p24	II
WIILGLNKIVRM	HIV	p24	II
WIILGLNKIVRMYSP	HIV	p24	II
WIILGLNKIVRMYSPTSI	HIV	p24	II
WILLGLNKIVRMYSPTSI	HIV	p24	II
WKVVEEKAFSPEVIPMF	HIV	p24	II
WMTSNPPVPVG	HIV	p24	II
WVKVVEEKAFSPEVIPM	HIV	p24	II
YKTLRAEQA	HIV	p24	II
YKTLRAEQASQEVKN	HIV	p24	II
YVDRFFKTLRAEQATQD	HIV	p24	II
YVDRFYKTLRAEQASQE	HIV	p24	II
ACQGVGGPGHKARVLA	HIV	p24-p2p7p1p6	II
GHKARVLAEAMSQVTN	HIV	p24-p2p7p1p6	II
DCTERQANFLG	HIV	p2p7p1p6	II
EAMSQVTNSATIMMQR	HIV	p2p7p1p6	II
EPIDKELYPLASLRS	HIV	p2p7p1p6	II
ESFRSGVETTTPPQK	HIV	p2p7p1p6	II
FEETTPAPPKQ	HIV	p2p7p1p6	II
FLQSRPEPTAPPEESFRF	HIV	p2p7p1p6	II
FNCGKEGHTARNCRA	HIV	p2p7p1p6	II
GEETTTPSQKQEPIDKEL	HIV	p2p7p1p6	II
GKIWPSHKGRPGNFLQS	HIV	p2p7p1p6	II
GRPGNFLQSRPEPTA	HIV	p2p7p1p6	II
HIAKNCRAPRKKGCWK	HIV	p2p7p1p6	II
KDREPLTSLKS	HIV	p2p7p1p6	II
KEGHQMKDCTERQAN	HIV	p2p7p1p6	II
MKDCTERQANFLGKI	HIV	p2p7p1p6	II
QKQEPIDKELYPLASLR	HIV	p2p7p1p6	II
QRGNFRNQRKTVKCF	HIV	p2p7p1p6	II
RAPRKKGCWKCGKEGH	HIV	p2p7p1p6	II
RNQRKTVKCFNCGKEG	HIV	p2p7p1p6	II
RQANFLGKIWPSHKGR	HIV	p2p7p1p6	II
TNSATIMMQRGNFRNQR	HIV	p2p7p1p6	II

VKCFNCGKGEH	HIV	p2p7p1p6	II
FIKVRQYDQIPIEICGKK	HIV	Protease	II
DEELIRTVRLIKLLY	HIV	Rev	II
GTRQARRNRRRRWRER	HIV	Rev	II
KTVRLIKFLYQSNPPPS	HIV	Rev	II
RRRRWRERQRQIHSIS	HIV	Rev	II
SNPPPNPEGTRQARR	HIV	Rev	II
EICTEMEKEGKISKIGP	HIV	RT	II
EKVYLAWVPAHKGIG	HIV	RT	II
FRKYTAFTIPSINNE	HIV	RT	II
GKTPKFKLPIQKETW	HIV	RT	II
GTKALTEVIPLTEEA	HIV	RT	II
IGQHRTKIEELRQHL	HIV	RT	II
KDSSTVNDIQKLVGK	HIV	RT	II
KDSWTVNDIQKLVGK	HIV	RT	II
KDSWTWNDIQKLVGK	HIV	RT	II
KVYLAWVPAHKGIGG	HIV	RT	II
LAENREILKEPVHGV	HIV	RT	II
LEKEPIVGAETFYVD	HIV	RT	II
MTKILEPFRKQNPDIVIY	HIV	RT	II
PLTEEAELELAENRE	HIV	RT	II
QKLWGKLNWASQIYP	HIV	RT	II
SPAIFQCSSMTKILEP	HIV	RT	II
SPAIFQSSMTKILEP	HIV	RT	II
SSTVNDIQKLV	HIV	RT	II
TEMEKEGKISKIGPE	HIV	RT	II
TYQIYQEPFKNLKTG	HIV	RT	II
WEFVNTPPLVKLWYQ	HIV	RT	II
WRQLCKLLRGTKALT	HIV	RT	II
SAGIRKVLFLD	HIV	RT-Integrase	II
HCQVCFITKALGISYG	HIV	Tat	II
HCQVCFMTKGLGISYG	HIV	Tat	II
HQASLSKQPTSQPRGD	HIV	Tat	II
KALGISYGRKKRRQR	HIV	Tat	II
QPKTACTNCYCKKCCF	HIV	Tat	II
QPKTACTNCYCKRCCF	HIV	Tat	II
LGQGVSIEWRKQRYST	HIV	Vif	II
VITTYWGLHTGE	HIV	Vif	II
DTWAGVEAIIRILQQ	HIV	Vpr	II
ETYGDTWAGVEAIIR	HIV	Vpr	II
IHFRIGCRHSRIGVT	HIV	Vpr	II
LQQLLFIHFRIGCRHS	HIV	Vpr	II
PRIWLHGLGQHIYET	HIV	Vpr	II
QLLFIHFRIGCRHSR	HIV	Vpr	II
RHFPRIWLHGLGQHI	HIV	Vpr	II
SRIGILRQRRARNGASRS	HIV	Vpr	II
AIVVWSIVLIEYRKIL	HIV	Vpu	II
