# Supplementary material for: Equivalent T Cell Epitope Promiscuity in Ecologically Diverse Human Pathogens
Source: PLoS One. 2013 Aug 9;8(8):e73124. doi: 10.1371/journal.pone.0073124 (PMC3739752; doi:10.1371/journal.pone.0073124)
Supplement: Table S2 — (PDF) [file pone.0073124.s005.pdf]

**HLA-DR**	**HLA-A**	**HLA-B**
DRB1_1381	HLA-A01:01	HLA-B07:02
DRB1_1389	HLA-A01:02	HLA-B07:03
DRB5_0205	HLA-A01:03	HLA-B07:04
DRB1_1388	HLA-A01:06	HLA-B07:05
DRB1_0106	HLA-A01:07	HLA-B07:06
DRB1_0110	HLA-A01:08	HLA-B07:07
DRB1_0123	HLA-A01:09	HLA-B07:08
DRB1_0840	HLA-A01:10	HLA-B07:09
DRB1_0120	HLA-A01:12	HLA-B07:10
DRB1_0102	HLA-A01:13	HLA-B07:100
DRB1_13101	HLA-A01:14	HLA-B07:101
DRB1_1465	HLA-A01:17	HLA-B07:102
DRB1_1390	HLA-A01:19	HLA-B07:103
DRB1_1421	HLA-A01:20	HLA-B07:104
DRB1_1358	HLA-A01:21	HLA-B07:105
DRB1_0822	HLA-A01:23	HLA-B07:106
DRB1_1394	HLA-A01:24	HLA-B07:107
DRB1_0806	HLA-A01:25	HLA-B07:108
DRB5_0204	HLA-A01:26	HLA-B07:109
DRB5_0202	HLA-A01:28	HLA-B07:11
DRB1_0810	HLA-A01:29	HLA-B07:110
DRB1_0812	HLA-A01:30	HLA-B07:112
DRB1_0121	HLA-A01:32	HLA-B07:113
DRB1_1375	HLA-A01:33	HLA-B07:114
DRB5_0106	HLA-A01:35	HLA-B07:115
DRB1_0467	HLA-A01:36	HLA-B07:12
DRB1_0104	HLA-A01:37	HLA-B07:13
DRB1_0126	HLA-A01:38	HLA-B07:14
DRB1_1303	HLA-A01:39	HLA-B07:15
DRB1_1395	HLA-A01:40	HLA-B07:16
DRB1_1310	HLA-A01:41	HLA-B07:17
DRB1_1142	HLA-A01:42	HLA-B07:18
DRB1_1212	HLA-A01:43	HLA-B07:19
DRB1_1366	HLA-A01:44	HLA-B07:20
DRB1_1157	HLA-A01:45	HLA-B07:21
DRB1_0818	HLA-A01:46	HLA-B07:22
DRB1_1452	HLA-A01:47	HLA-B07:23
DRB1_0908	HLA-A01:48	HLA-B07:24
DRB1_1192	HLA-A01:49	HLA-B07:25
DRB1_0109	HLA-A01:50	HLA-B07:26
DRB1_1413	HLA-A01:51	HLA-B07:27

DRB1_1480	HLA-A01:54	HLA-B07:28
DRB1_0831	HLA-A01:55	HLA-B07:29
DRB1_0805	HLA-A01:58	HLA-B07:30
DRB1_0101	HLA-A01:59	HLA-B07:31
DRB1_0105	HLA-A01:60	HLA-B07:32
DRB1_0107	HLA-A01:61	HLA-B07:33
DRB1_0108	HLA-A01:62	HLA-B07:34
DRB1_0112	HLA-A01:63	HLA-B07:35
DRB1_0119	HLA-A01:64	HLA-B07:36
DRB1_0122	HLA-A01:65	HLA-B07:37
DRB1_0125	HLA-A01:66	HLA-B07:38
DRB1_0127	HLA-A02:01	HLA-B07:39
DRB1_0128	HLA-A02:02	HLA-B07:40
DRB1_0130	HLA-A02:03	HLA-B07:41
DRB1_0131	HLA-A02:04	HLA-B07:42
DRB1_0132	HLA-A02:05	HLA-B07:43
DRB1_1002	HLA-A02:06	HLA-B07:44
DRB1_1134	HLA-A02:07	HLA-B07:45
DRB1_1344	HLA-A02:08	HLA-B07:46
DRB1_1417	HLA-A02:09	HLA-B07:47
DRB1_0906	HLA-A02:10	HLA-B07:48
DRB1_1332	HLA-A02:101	HLA-B07:50
DRB1_1348	HLA-A02:102	HLA-B07:51
DRB1_1478	HLA-A02:103	HLA-B07:52
DRB1_1204	HLA-A02:104	HLA-B07:53
DRB1_1209	HLA-A02:105	HLA-B07:54
DRB1_1406	HLA-A02:106	HLA-B07:55
DRB1_1420	HLA-A02:107	HLA-B07:56
DRB1_1483	HLA-A02:108	HLA-B07:57
DRB1_0118	HLA-A02:109	HLA-B07:58
DRB1_1211	HLA-A02:11	HLA-B07:59
DRB1_0129	HLA-A02:110	HLA-B07:60
DRB1_1429	HLA-A02:111	HLA-B07:61
DRB1_1113	HLA-A02:112	HLA-B07:62
DRB1_0706	HLA-A02:114	HLA-B07:63
DRB1_1321	HLA-A02:115	HLA-B07:64
DRB1_1304	HLA-A02:116	HLA-B07:65
DRB1_1393	HLA-A02:117	HLA-B07:66
DRB1_1221	HLA-A02:118	HLA-B07:68
DRB1_1481	HLA-A02:119	HLA-B07:69
DRB1_0413	HLA-A02:12	HLA-B07:70
DRB1_1474	HLA-A02:120	HLA-B07:71

DRB1_0113	HLA-A02:121	HLA-B07:72
DRB1_1431	HLA-A02:122	HLA-B07:73
DRB1_1141	HLA-A02:123	HLA-B07:74
DRB1_1512	HLA-A02:124	HLA-B07:75
DRB1_0409	HLA-A02:126	HLA-B07:76
DRB1_1104	HLA-A02:127	HLA-B07:77
DRB1_1135	HLA-A02:128	HLA-B07:78
DRB1_1138	HLA-A02:129	HLA-B07:79
DRB1_1143	HLA-A02:13	HLA-B07:80
DRB1_1144	HLA-A02:130	HLA-B07:81
DRB1_1146	HLA-A02:131	HLA-B07:82
DRB1_1158	HLA-A02:132	HLA-B07:83
DRB1_1160	HLA-A02:133	HLA-B07:84
DRB1_1177	HLA-A02:134	HLA-B07:85
DRB1_1178	HLA-A02:135	HLA-B07:86
DRB1_1202	HLA-A02:136	HLA-B07:87
DRB1_1213	HLA-A02:137	HLA-B07:88
DRB1_1215	HLA-A02:138	HLA-B07:89
DRB1_1218	HLA-A02:139	HLA-B07:90
DRB1_1223	HLA-A02:14	HLA-B07:91
DRB1_1311	HLA-A02:140	HLA-B07:92
DRB1_1342	HLA-A02:141	HLA-B07:93
DRB1_1349	HLA-A02:142	HLA-B07:94
DRB1_1136	HLA-A02:143	HLA-B07:95
DRB1_1208	HLA-A02:144	HLA-B07:96
DRB1_1320	HLA-A02:145	HLA-B07:97
DRB1_1201	HLA-A02:146	HLA-B07:98
DRB1_1203	HLA-A02:147	HLA-B07:99
DRB1_1205	HLA-A02:148	HLA-B08:01
DRB1_1206	HLA-A02:149	HLA-B08:02
DRB1_1207	HLA-A02:150	HLA-B08:03
DRB1_1210	HLA-A02:151	HLA-B08:04
DRB1_1214	HLA-A02:152	HLA-B08:05
DRB1_1217	HLA-A02:153	HLA-B08:07
DRB1_1219	HLA-A02:154	HLA-B08:09
DRB1_1333	HLA-A02:155	HLA-B08:10
DRB1_1103	HLA-A02:156	HLA-B08:11
DRB1_1163	HLA-A02:157	HLA-B08:12
DRB1_1176	HLA-A02:158	HLA-B08:13
DRB1_1185	HLA-A02:159	HLA-B08:14
DRB1_1324	HLA-A02:16	HLA-B08:15
DRB1_1106	HLA-A02:160	HLA-B08:16

DRB1_1147	HLA-A02:161	HLA-B08:17
DRB1_0410	HLA-A02:162	HLA-B08:18
DRB1_1150	HLA-A02:163	HLA-B08:20
DRB1_1156	HLA-A02:164	HLA-B08:21
DRB1_1188	HLA-A02:165	HLA-B08:22
DRB5_0103	HLA-A02:166	HLA-B08:23
DRB1_1312	HLA-A02:167	HLA-B08:24
DRB1_1419	HLA-A02:168	HLA-B08:25
DRB1_1330	HLA-A02:169	HLA-B08:26
DRB1_1179	HLA-A02:17	HLA-B08:27
DRB1_0115	HLA-A02:170	HLA-B08:28
DRB1_1484	HLA-A02:171	HLA-B08:29
DRB1_1317	HLA-A02:172	HLA-B08:31
DRB1_1437	HLA-A02:173	HLA-B08:32
DRB1_0804	HLA-A02:174	HLA-B08:33
DRB1_0902	HLA-A02:175	HLA-B08:34
DRB1_1118	HLA-A02:176	HLA-B08:35
DRB1_1306	HLA-A02:177	HLA-B08:36
DRB1_1415	HLA-A02:178	HLA-B08:37
DRB1_1523	HLA-A02:179	HLA-B08:38
DRB1_0828	HLA-A02:18	HLA-B08:39
DRB1_0837	HLA-A02:180	HLA-B08:40
DRB1_1167	HLA-A02:181	HLA-B08:41
DRB1_1183	HLA-A02:182	HLA-B08:42
DRB1_0114	HLA-A02:183	HLA-B08:43
DRB1_0111	HLA-A02:184	HLA-B08:44
DRB1_0124	HLA-A02:185	HLA-B08:45
DRB1_1485	HLA-A02:186	HLA-B08:46
DRB1_0904	HLA-A02:187	HLA-B08:47
DRB1_0903	HLA-A02:188	HLA-B08:48
DRB1_0116	HLA-A02:189	HLA-B08:49
DRB3_0201	HLA-A02:19	HLA-B08:50
DRB3_0224	HLA-A02:190	HLA-B08:51
DRB1_0832	HLA-A02:191	HLA-B08:52
DRB1_1159	HLA-A02:192	HLA-B08:53
DRB1_0434	HLA-A02:193	HLA-B08:54
DRB1_1319	HLA-A02:194	HLA-B08:55
DRB1_1353	HLA-A02:195	HLA-B08:56
DRB1_0117	HLA-A02:196	HLA-B08:57
DRB1_1309	HLA-A02:197	HLA-B08:58
DRB1_1371	HLA-A02:198	HLA-B08:59
DRB1_0464	HLA-A02:199	HLA-B08:60

DRB1_0803	HLA-A02:20	HLA-B08:61
DRB1_0814	HLA-A02:200	HLA-B08:62
DRB1_0823	HLA-A02:201	HLA-B13:01
DRB1_0827	HLA-A02:202	HLA-B13:02
DRB1_0833	HLA-A02:203	HLA-B13:03
DRB1_0835	HLA-A02:204	HLA-B13:04
DRB1_0836	HLA-A02:205	HLA-B13:06
DRB1_0838	HLA-A02:206	HLA-B13:09
DRB5_0203	HLA-A02:207	HLA-B13:10
DRB1_1505	HLA-A02:208	HLA-B13:11
DRB1_1360	HLA-A02:209	HLA-B13:12
DRB5_0102	HLA-A02:21	HLA-B13:13
DRB5_0108N	HLA-A02:210	HLA-B13:14
DRB1_1220	HLA-A02:211	HLA-B13:15
DRB1_1378	HLA-A02:212	HLA-B13:16
DRB1_0466	HLA-A02:213	HLA-B13:17
DRB1_1412	HLA-A02:214	HLA-B13:18
DRB1_0103	HLA-A02:215	HLA-B13:19
DRB1_1108	HLA-A02:216	HLA-B13:20
DRB1_1356	HLA-A02:217	HLA-B13:21
DRB1_1385	HLA-A02:218	HLA-B13:22
DRB1_1148	HLA-A02:219	HLA-B13:23
DRB1_1308	HLA-A02:22	HLA-B13:25
DRB1_1370	HLA-A02:220	HLA-B13:26
DRB1_1372	HLA-A02:221	HLA-B13:27
DRB1_1384	HLA-A02:224	HLA-B13:28
DRB1_1377	HLA-A02:228	HLA-B13:29
DRB1_0801	HLA-A02:229	HLA-B13:30
DRB1_0816	HLA-A02:230	HLA-B13:31
DRB1_0826	HLA-A02:231	HLA-B13:32
DRB1_0839	HLA-A02:232	HLA-B13:33
DRB1_1001	HLA-A02:233	HLA-B13:34
DRB1_1003	HLA-A02:234	HLA-B13:35
DRB1_1337	HLA-A02:235	HLA-B13:36
DRB1_1173	HLA-A02:236	HLA-B13:37
DRB5_0111	HLA-A02:237	HLA-B13:38
DRB1_1193	HLA-A02:238	HLA-B13:39
DRB1_1315	HLA-A02:239	HLA-B14:01
DRB1_1357	HLA-A02:24	HLA-B14:02
DRB1_1432	HLA-A02:240	HLA-B14:03
DRB1_1354	HLA-A02:241	HLA-B14:04
DRB1_1614	HLA-A02:242	HLA-B14:05

DRB1_0712	HLA-A02:243	HLA-B14:06
DRB1_1549	HLA-A02:244	HLA-B14:08
DRB1_0701	HLA-A02:245	HLA-B14:09
DRB1_0703	HLA-A02:246	HLA-B14:10
DRB1_0705	HLA-A02:247	HLA-B14:11
DRB1_0707	HLA-A02:248	HLA-B14:12
DRB1_0708	HLA-A02:249	HLA-B14:13
DRB1_0713	HLA-A02:25	HLA-B14:14
DRB1_0714	HLA-A02:251	HLA-B14:15
DRB1_0715	HLA-A02:252	HLA-B14:16
DRB1_0716	HLA-A02:253	HLA-B14:17
DRB1_0717	HLA-A02:254	HLA-B14:18
DRB1_0719	HLA-A02:255	HLA-B15:01
DRB1_1476	HLA-A02:256	HLA-B15:02
DRB1_0315	HLA-A02:257	HLA-B15:03
DRB1_1121	HLA-A02:258	HLA-B15:04
DRB3_0301	HLA-A02:259	HLA-B15:05
DRB1_1102	HLA-A02:26	HLA-B15:06
DRB1_1116	HLA-A02:260	HLA-B15:07
DRB1_1165	HLA-A02:261	HLA-B15:08
DRB1_1301	HLA-A02:262	HLA-B15:09
DRB1_1322	HLA-A02:263	HLA-B15:10
DRB1_1335	HLA-A02:264	HLA-B15:101
DRB1_1351	HLA-A02:265	HLA-B15:102
DRB1_1352	HLA-A02:266	HLA-B15:103
DRB1_1359	HLA-A02:27	HLA-B15:104
DRB1_1364	HLA-A02:28	HLA-B15:105
DRB1_1368	HLA-A02:29	HLA-B15:106
DRB1_1369	HLA-A02:30	HLA-B15:107
DRB1_1380	HLA-A02:31	HLA-B15:108
DRB1_1383	HLA-A02:33	HLA-B15:109
DRB1_1387	HLA-A02:34	HLA-B15:11
DRB1_1391	HLA-A02:35	HLA-B15:110
DRB1_1392	HLA-A02:36	HLA-B15:112
DRB1_1434	HLA-A02:37	HLA-B15:113
DRB1_1611	HLA-A02:38	HLA-B15:114
DRB1_0901	HLA-A02:39	HLA-B15:115
DRB1_0909	HLA-A02:40	HLA-B15:116
DRB1_0487	HLA-A02:41	HLA-B15:117
DRB1_1411	HLA-A02:42	HLA-B15:118
DRB5_0101	HLA-A02:44	HLA-B15:119
DRB5_0105	HLA-A02:45	HLA-B15:12

DRB5_0113	HLA-A02:46	HLA-B15:120
DRB5_0114	HLA-A02:47	HLA-B15:121
DRB1_0829	HLA-A02:48	HLA-B15:122
DRB1_1338	HLA-A02:49	HLA-B15:123
DRB1_1365	HLA-A02:50	HLA-B15:124
DRB1_0324	HLA-A02:51	HLA-B15:125
DRB1_0479	HLA-A02:52	HLA-B15:126
DRB1_0405	HLA-A02:54	HLA-B15:127
DRB1_0429	HLA-A02:55	HLA-B15:128
DRB1_0430	HLA-A02:56	HLA-B15:129
DRB1_0445	HLA-A02:57	HLA-B15:13
DRB1_0448	HLA-A02:58	HLA-B15:131
DRB1_0477	HLA-A02:59	HLA-B15:132
DRB1_0483	HLA-A02:60	HLA-B15:133
DRB1_0484	HLA-A02:61	HLA-B15:134
DRB1_0489	HLA-A02:62	HLA-B15:135
DRB1_1343	HLA-A02:63	HLA-B15:136
DRB1_1172	HLA-A02:64	HLA-B15:137
DRB1_1313	HLA-A02:65	HLA-B15:138
DRB3_0214	HLA-A02:66	HLA-B15:139
DRB1_0401	HLA-A02:67	HLA-B15:14
DRB1_0416	HLA-A02:68	HLA-B15:140
DRB1_0426	HLA-A02:69	HLA-B15:141
DRB1_0433	HLA-A02:70	HLA-B15:142
DRB1_0463	HLA-A02:71	HLA-B15:143
DRB1_0476	HLA-A02:72	HLA-B15:144
DRB1_1430	HLA-A02:73	HLA-B15:145
DRB1_0709	HLA-A02:74	HLA-B15:146
DRB3_0215	HLA-A02:75	HLA-B15:147
DRB1_0820	HLA-A02:76	HLA-B15:148
DRB1_1612	HLA-A02:77	HLA-B15:15
DRB1_0311	HLA-A02:78	HLA-B15:150
DRB1_0428	HLA-A02:79	HLA-B15:151
DRB1_0435	HLA-A02:80	HLA-B15:152
DRB1_1398	HLA-A02:81	HLA-B15:153
DRB1_1433	HLA-A02:84	HLA-B15:154
DRB1_1503	HLA-A02:85	HLA-B15:155
DRB1_1615	HLA-A02:86	HLA-B15:156
DRB3_0204	HLA-A02:87	HLA-B15:157
DRB1_0412	HLA-A02:89	HLA-B15:158
DRB1_1602	HLA-A02:90	HLA-B15:159
DRB1_1610	HLA-A02:91	HLA-B15:16

DRB1_1616	HLA-A02:92	HLA-B15:160
DRB1_0404	HLA-A02:93	HLA-B15:161
DRB1_0423	HLA-A02:95	HLA-B15:162
DRB1_0440	HLA-A02:96	HLA-B15:163
DRB1_0468	HLA-A02:97	HLA-B15:164
DRB1_0470	HLA-A02:99	HLA-B15:165
DRB1_1184	HLA-A03:01	HLA-B15:166
DRB1_1327	HLA-A03:02	HLA-B15:167
DRB1_1170	HLA-A03:04	HLA-B15:168
DRB1_1379	HLA-A03:05	HLA-B15:169
DRB1_1536	HLA-A03:06	HLA-B15:17
DRB1_1409	HLA-A03:07	HLA-B15:170
DRB1_1518	HLA-A03:08	HLA-B15:171
DRB1_1125	HLA-A03:09	HLA-B15:172
DRB1_1318	HLA-A03:10	HLA-B15:173
DRB1_1386	HLA-A03:12	HLA-B15:174
DRB1_1463	HLA-A03:13	HLA-B15:175
DRB1_1535	HLA-A03:14	HLA-B15:176
DRB1_0411	HLA-A03:15	HLA-B15:177
DRB1_0424	HLA-A03:16	HLA-B15:178
DRB1_0442	HLA-A03:17	HLA-B15:179
DRB1_0491	HLA-A03:18	HLA-B15:18
DRB1_1186	HLA-A03:19	HLA-B15:180
DRB1_1316	HLA-A03:20	HLA-B15:183
DRB1_1469	HLA-A03:22	HLA-B15:184
DRB1_1528	HLA-A03:23	HLA-B15:185
DRB1_1501	HLA-A03:24	HLA-B15:186
DRB1_1506	HLA-A03:25	HLA-B15:187
DRB1_1509	HLA-A03:26	HLA-B15:188
DRB1_1513	HLA-A03:27	HLA-B15:189
DRB1_1516	HLA-A03:28	HLA-B15:19
DRB1_1520	HLA-A03:29	HLA-B15:191
DRB1_1522	HLA-A03:30	HLA-B15:192
DRB1_1524	HLA-A03:31	HLA-B15:193
DRB1_1532	HLA-A03:32	HLA-B15:194
DRB1_1533	HLA-A03:33	HLA-B15:195
DRB1_1540	HLA-A03:34	HLA-B15:196
DRB1_1541	HLA-A03:35	HLA-B15:197
DRB1_1542	HLA-A03:37	HLA-B15:198
DRB1_1543	HLA-A03:38	HLA-B15:199
DRB1_1545	HLA-A03:39	HLA-B15:20
DRB1_1546	HLA-A03:40	HLA-B15:200

DRB1_0307	HLA-A03:41	HLA-B15:201
DRB1_0321	HLA-A03:42	HLA-B15:202
DRB1_0349	HLA-A03:43	HLA-B15:21
DRB1_1107	HLA-A03:44	HLA-B15:23
DRB1_1117	HLA-A03:45	HLA-B15:24
DRB1_1168	HLA-A03:46	HLA-B15:25
DRB1_1329	HLA-A03:47	HLA-B15:27
DRB1_1402	HLA-A03:48	HLA-B15:28
DRB1_1405	HLA-A03:49	HLA-B15:29
DRB1_1423	HLA-A03:50	HLA-B15:30
DRB1_1441	HLA-A03:51	HLA-B15:31
DRB1_1456	HLA-A03:52	HLA-B15:32
DRB1_1459	HLA-A03:53	HLA-B15:33
DRB1_1491	HLA-A03:54	HLA-B15:34
DRB1_1494	HLA-A03:55	HLA-B15:35
DRB1_1496	HLA-A03:56	HLA-B15:36
DRB1_0303	HLA-A03:57	HLA-B15:37
DRB1_0306	HLA-A03:58	HLA-B15:38
DRB1_0326	HLA-A03:59	HLA-B15:39
DRB1_0331	HLA-A03:60	HLA-B15:40
DRB1_0347	HLA-A03:61	HLA-B15:42
DRB1_0415	HLA-A03:62	HLA-B15:43
DRB1_0436	HLA-A03:63	HLA-B15:44
DRB1_1155	HLA-A03:64	HLA-B15:45
DRB1_1105	HLA-A03:65	HLA-B15:46
DRB1_0458	HLA-A03:66	HLA-B15:47
DRB1_1181	HLA-A03:67	HLA-B15:48
DRB1_1355	HLA-A03:70	HLA-B15:49
DRB1_1504	HLA-A03:71	HLA-B15:50
DRB4_0101	HLA-A03:72	HLA-B15:51
DRB4_0103	HLA-A03:73	HLA-B15:52
DRB1_1154	HLA-A03:74	HLA-B15:53
DRB1_1361	HLA-A03:75	HLA-B15:54
DRB1_1418	HLA-A03:76	HLA-B15:55
DRB1_1507	HLA-A03:77	HLA-B15:56
DRB1_1548	HLA-A03:78	HLA-B15:57
DRB1_0457	HLA-A03:79	HLA-B15:58
DRB1_0813	HLA-A03:80	HLA-B15:60
DRB1_1114	HLA-A03:81	HLA-B15:61
DRB1_1120	HLA-A03:82	HLA-B15:62
DRB1_1191	HLA-A11:01	HLA-B15:63
DRB1_1302	HLA-A11:02	HLA-B15:64

DRB1_1323	HLA-A11:03	HLA-B15:65
DRB1_1334	HLA-A11:04	HLA-B15:66
DRB1_1336	HLA-A11:05	HLA-B15:67
DRB1_1346	HLA-A11:06	HLA-B15:68
DRB1_1373	HLA-A11:07	HLA-B15:69
DRB1_1374	HLA-A11:08	HLA-B15:70
DRB1_1397	HLA-A11:09	HLA-B15:71
DRB1_1399	HLA-A11:10	HLA-B15:72
DRB1_1495	HLA-A11:11	HLA-B15:73
DRB1_1527	HLA-A11:12	HLA-B15:74
DRB1_1101	HLA-A11:13	HLA-B15:75
DRB1_1109	HLA-A11:14	HLA-B15:76
DRB1_1110	HLA-A11:15	HLA-B15:77
DRB1_1111	HLA-A11:16	HLA-B15:78
DRB1_1112	HLA-A11:17	HLA-B15:80
DRB1_1115	HLA-A11:18	HLA-B15:81
DRB1_1119	HLA-A11:19	HLA-B15:82
DRB1_1124	HLA-A11:20	HLA-B15:83
DRB1_1128	HLA-A11:22	HLA-B15:84
DRB1_1129	HLA-A11:23	HLA-B15:85
DRB1_1133	HLA-A11:24	HLA-B15:86
DRB1_1139	HLA-A11:25	HLA-B15:87
DRB1_1149	HLA-A11:26	HLA-B15:88
DRB1_1151	HLA-A11:27	HLA-B15:89
DRB1_1161	HLA-A11:29	HLA-B15:90
DRB1_1162	HLA-A11:30	HLA-B15:91
DRB1_1166	HLA-A11:31	HLA-B15:92
DRB1_1174	HLA-A11:32	HLA-B15:93
DRB1_1175	HLA-A11:33	HLA-B15:95
DRB1_1190	HLA-A11:34	HLA-B15:96
DRB1_1194	HLA-A11:35	HLA-B15:97
DRB1_1195	HLA-A11:36	HLA-B15:98
DRB1_1305	HLA-A11:37	HLA-B15:99
DRB1_1314	HLA-A11:38	HLA-B18:01
DRB1_1350	HLA-A11:39	HLA-B18:02
DRB1_1362	HLA-A11:40	HLA-B18:03
DRB1_1363	HLA-A11:41	HLA-B18:04
DRB1_1382	HLA-A11:42	HLA-B18:05
DRB1_1404	HLA-A11:43	HLA-B18:06
DRB1_1428	HLA-A11:44	HLA-B18:07
DRB1_1461	HLA-A11:45	HLA-B18:08
DRB3_0212	HLA-A11:46	HLA-B18:09

DRB1_0480	HLA-A11:47	HLA-B18:10
DRB1_1416	HLA-A11:48	HLA-B18:11
DRB1_1448	HLA-A11:49	HLA-B18:12
DRB1_1510	HLA-A11:51	HLA-B18:13
DRB1_1521	HLA-A11:53	HLA-B18:14
DRB1_1534	HLA-A11:54	HLA-B18:15
DRB1_0455	HLA-A11:55	HLA-B18:18
DRB1_1331	HLA-A11:56	HLA-B18:19
DRB3_0213	HLA-A11:57	HLA-B18:20
DRB1_0301	HLA-A11:58	HLA-B18:21
DRB1_0304	HLA-A11:59	HLA-B18:22
DRB1_0308	HLA-A11:60	HLA-B18:24
DRB1_0318	HLA-A11:61	HLA-B18:25
DRB1_0322	HLA-A11:62	HLA-B18:26
DRB1_0323	HLA-A11:63	HLA-B18:27
DRB1_0325	HLA-A11:64	HLA-B18:28
DRB1_0328	HLA-A23:01	HLA-B18:29
DRB1_0330	HLA-A23:02	HLA-B18:30
DRB1_0332	HLA-A23:03	HLA-B18:31
DRB1_0333	HLA-A23:04	HLA-B18:32
DRB1_0334	HLA-A23:05	HLA-B18:33
DRB1_0336	HLA-A23:06	HLA-B18:34
DRB1_0337	HLA-A23:09	HLA-B18:35
DRB1_0339	HLA-A23:10	HLA-B18:36
DRB1_0343	HLA-A23:12	HLA-B18:37
DRB1_0344	HLA-A23:13	HLA-B18:38
DRB1_0345	HLA-A23:14	HLA-B18:39
DRB1_0346	HLA-A23:15	HLA-B18:40
DRB1_0348	HLA-A23:16	HLA-B18:41
DRB1_0350	HLA-A23:17	HLA-B18:42
DRB1_0351	HLA-A23:18	HLA-B18:43
DRB1_0352	HLA-A23:20	HLA-B18:44
DRB1_0354	HLA-A23:21	HLA-B18:45
DRB1_0355	HLA-A23:22	HLA-B18:46
DRB1_0450	HLA-A23:23	HLA-B18:47
DRB1_0711	HLA-A23:24	HLA-B18:48
DRB1_1164	HLA-A23:25	HLA-B18:49
DRB1_1446	HLA-A23:26	HLA-B18:50
DRB1_1531	HLA-A24:02	HLA-B27:01
DRB3_0202	HLA-A24:03	HLA-B27:02
DRB3_0210	HLA-A24:04	HLA-B27:03
DRB3_0218	HLA-A24:05	HLA-B27:04

DRB3_0220	HLA-A24:06	HLA-B27:05
DRB3_0223	HLA-A24:07	HLA-B27:06
DRB1_0437	HLA-A24:08	HLA-B27:07
DRB1_1216	HLA-A24:10	HLA-B27:08
DRB1_1424	HLA-A24:100	HLA-B27:09
DRB1_1443	HLA-A24:101	HLA-B27:10
DRB1_0320	HLA-A24:102	HLA-B27:11
DRB1_0417	HLA-A24:103	HLA-B27:12
DRB1_0446	HLA-A24:104	HLA-B27:13
DRB1_0472	HLA-A24:105	HLA-B27:14
DRB1_0824	HLA-A24:106	HLA-B27:15
DRB1_1130	HLA-A24:107	HLA-B27:16
DRB1_1367	HLA-A24:108	HLA-B27:17
DRB1_1447	HLA-A24:109	HLA-B27:18
DRB1_1449	HLA-A24:110	HLA-B27:19
DRB1_1462	HLA-A24:111	HLA-B27:20
DRB1_1464	HLA-A24:112	HLA-B27:21
DRB1_0317	HLA-A24:113	HLA-B27:23
DRB1_0335	HLA-A24:114	HLA-B27:24
DRB1_0453	HLA-A24:115	HLA-B27:25
DRB1_0475	HLA-A24:116	HLA-B27:26
DRB1_0907	HLA-A24:117	HLA-B27:27
DRB1_1182	HLA-A24:118	HLA-B27:28
DRB1_13100	HLA-A24:119	HLA-B27:29
DRB1_1453	HLA-A24:120	HLA-B27:30
DRB1_1455	HLA-A24:121	HLA-B27:31
DRB1_1479	HLA-A24:122	HLA-B27:32
DRB3_0209	HLA-A24:123	HLA-B27:33
DRB3_0221	HLA-A24:124	HLA-B27:34
DRB5_0112	HLA-A24:125	HLA-B27:35
DRB1_0314	HLA-A24:126	HLA-B27:36
DRB1_0327	HLA-A24:127	HLA-B27:37
DRB1_0408	HLA-A24:128	HLA-B27:38
DRB1_0419	HLA-A24:129	HLA-B27:39
DRB1_0449	HLA-A24:13	HLA-B27:40
DRB1_0461	HLA-A24:130	HLA-B27:41
DRB1_0462	HLA-A24:131	HLA-B27:42
DRB1_0825	HLA-A24:133	HLA-B27:43
DRB1_1180	HLA-A24:134	HLA-B27:44
DRB1_1187	HLA-A24:135	HLA-B27:45
DRB1_1339	HLA-A24:136	HLA-B27:46
DRB1_1341	HLA-A24:137	HLA-B27:47

DRB1_1376	HLA-A24:138	HLA-B27:48
DRB1_1471	HLA-A24:139	HLA-B27:49
DRB1_1473	HLA-A24:14	HLA-B27:50
DRB1_1525	HLA-A24:140	HLA-B27:51
DRB1_1605	HLA-A24:141	HLA-B27:52
DRB1_1607	HLA-A24:142	HLA-B27:53
DRB3_0205	HLA-A24:143	HLA-B27:54
DRB1_0438	HLA-A24:144	HLA-B27:55
DRB1_0443	HLA-A24:15	HLA-B27:56
DRB1_0444	HLA-A24:17	HLA-B27:57
DRB1_0451	HLA-A24:18	HLA-B27:58
DRB1_0473	HLA-A24:19	HLA-B27:60
DRB1_0704	HLA-A24:20	HLA-B27:61
DRB1_0811	HLA-A24:21	HLA-B27:62
DRB1_1131	HLA-A24:22	HLA-B27:63
DRB1_1137	HLA-A24:23	HLA-B27:67
DRB1_1189	HLA-A24:24	HLA-B27:68
DRB1_1307	HLA-A24:25	HLA-B27:69
DRB1_1401	HLA-A24:26	HLA-B35:01
DRB1_1403	HLA-A24:27	HLA-B35:02
DRB1_1426	HLA-A24:28	HLA-B35:03
DRB1_1435	HLA-A24:29	HLA-B35:04
DRB1_1440	HLA-A24:30	HLA-B35:05
DRB1_1454	HLA-A24:31	HLA-B35:06
DRB1_1458	HLA-A24:32	HLA-B35:07
DRB1_1460	HLA-A24:33	HLA-B35:08
DRB1_1470	HLA-A24:34	HLA-B35:09
DRB1_1472	HLA-A24:35	HLA-B35:10
DRB1_1477	HLA-A24:37	HLA-B35:100
DRB1_1486	HLA-A24:38	HLA-B35:101
DRB1_1487	HLA-A24:39	HLA-B35:102
DRB1_1488	HLA-A24:41	HLA-B35:103
DRB1_1490	HLA-A24:42	HLA-B35:104
DRB1_1497	HLA-A24:43	HLA-B35:105
DRB1_1609	HLA-A24:44	HLA-B35:106
DRB3_0211	HLA-A24:46	HLA-B35:107
DRB3_0222	HLA-A24:47	HLA-B35:108
DRB3_0225	HLA-A24:49	HLA-B35:109
DRB5_0104	HLA-A24:50	HLA-B35:11
DRB1_0302	HLA-A24:51	HLA-B35:110
DRB1_0313	HLA-A24:52	HLA-B35:111
DRB1_0341	HLA-A24:53	HLA-B35:112

DRB1_0353	HLA-A24:54	HLA-B35:113
DRB1_0403	HLA-A24:55	HLA-B35:114
DRB1_0406	HLA-A24:56	HLA-B35:115
DRB1_0418	HLA-A24:57	HLA-B35:116
DRB1_0427	HLA-A24:58	HLA-B35:117
DRB1_0439	HLA-A24:59	HLA-B35:118
DRB1_0441	HLA-A24:61	HLA-B35:119
DRB1_0447	HLA-A24:62	HLA-B35:12
DRB1_0452	HLA-A24:63	HLA-B35:120
DRB1_0460	HLA-A24:64	HLA-B35:121
DRB1_0471	HLA-A24:66	HLA-B35:122
DRB1_0485	HLA-A24:67	HLA-B35:123
DRB1_0486	HLA-A24:68	HLA-B35:124
DRB1_0905	HLA-A24:69	HLA-B35:125
DRB1_1152	HLA-A24:70	HLA-B35:126
DRB1_1222	HLA-A24:71	HLA-B35:127
DRB1_1326	HLA-A24:72	HLA-B35:128
DRB1_1396	HLA-A24:73	HLA-B35:13
DRB1_1408	HLA-A24:74	HLA-B35:131
DRB1_1442	HLA-A24:75	HLA-B35:132
DRB1_1475	HLA-A24:76	HLA-B35:133
DRB1_1499	HLA-A24:77	HLA-B35:135
DRB1_1530	HLA-A24:78	HLA-B35:136
DRB3_0105	HLA-A24:79	HLA-B35:137
DRB3_0216	HLA-A24:80	HLA-B35:138
DRB1_0338	HLA-A24:81	HLA-B35:139
DRB1_0340	HLA-A24:82	HLA-B35:14
DRB1_0342	HLA-A24:85	HLA-B35:140
DRB1_0456	HLA-A24:87	HLA-B35:141
DRB1_0459	HLA-A24:88	HLA-B35:142
DRB1_0802	HLA-A24:89	HLA-B35:143
DRB1_0807	HLA-A24:91	HLA-B35:144
DRB1_0809	HLA-A24:92	HLA-B35:15
DRB1_0819	HLA-A24:93	HLA-B35:16
DRB1_0821	HLA-A24:94	HLA-B35:17
DRB1_0830	HLA-A24:95	HLA-B35:18
DRB1_0834	HLA-A24:96	HLA-B35:19
DRB1_1127	HLA-A24:97	HLA-B35:20
DRB1_1132	HLA-A24:98	HLA-B35:21
DRB1_1145	HLA-A24:99	HLA-B35:22
DRB1_1169	HLA-A25:01	HLA-B35:23
DRB1_1196	HLA-A25:02	HLA-B35:24

DRB1_1414	HLA-A25:03	HLA-B35:25
DRB1_1422	HLA-A25:04	HLA-B35:26
DRB1_1425	HLA-A25:05	HLA-B35:27
DRB1_1436	HLA-A25:06	HLA-B35:28
DRB1_1439	HLA-A25:07	HLA-B35:29
DRB1_1444	HLA-A25:08	HLA-B35:30
DRB1_1445	HLA-A25:09	HLA-B35:31
DRB1_1450	HLA-A25:10	HLA-B35:32
DRB1_1451	HLA-A25:11	HLA-B35:33
DRB1_1468	HLA-A25:13	HLA-B35:34
DRB3_0101	HLA-A26:01	HLA-B35:35
DRB3_0104	HLA-A26:02	HLA-B35:36
DRB3_0108	HLA-A26:03	HLA-B35:37
DRB3_0111	HLA-A26:04	HLA-B35:38
DRB3_0112	HLA-A26:05	HLA-B35:39
DRB3_0113	HLA-A26:06	HLA-B35:41
DRB3_0303	HLA-A26:07	HLA-B35:42
DRB1_0305	HLA-A26:08	HLA-B35:43
DRB1_0329	HLA-A26:09	HLA-B35:44
DRB1_0465	HLA-A26:10	HLA-B35:45
DRB1_0478	HLA-A26:12	HLA-B35:46
DRB1_1153	HLA-A26:13	HLA-B35:47
DRB1_1407	HLA-A26:14	HLA-B35:48
DRB1_1482	HLA-A26:15	HLA-B35:49
DRB1_1529	HLA-A26:16	HLA-B35:50
DRB1_1537	HLA-A26:17	HLA-B35:51
DRB1_1601	HLA-A26:18	HLA-B35:52
DRB1_1603	HLA-A26:19	HLA-B35:54
DRB1_1608	HLA-A26:20	HLA-B35:55
DRB3_0109	HLA-A26:21	HLA-B35:56
DRB3_0114	HLA-A26:22	HLA-B35:57
DRB3_0219	HLA-A26:23	HLA-B35:58
DRB1_0310	HLA-A26:24	HLA-B35:59
DRB1_0319	HLA-A26:26	HLA-B35:60
DRB1_0402	HLA-A26:27	HLA-B35:61
DRB1_0431	HLA-A26:28	HLA-B35:62
DRB1_0808	HLA-A26:29	HLA-B35:63
DRB1_1438	HLA-A26:30	HLA-B35:64
DRB1_1457	HLA-A26:31	HLA-B35:66
DRB1_1467	HLA-A26:32	HLA-B35:67
DRB1_1489	HLA-A26:33	HLA-B35:68
DRB1_1502	HLA-A26:34	HLA-B35:69

DRB1_1508	HLA-A26:35	HLA-B35:70
DRB1_1511	HLA-A26:36	HLA-B35:71
DRB1_1514	HLA-A26:37	HLA-B35:72
DRB1_1515	HLA-A26:38	HLA-B35:74
DRB1_1519	HLA-A26:39	HLA-B35:75
DRB1_1526	HLA-A26:40	HLA-B35:76
DRB1_1539	HLA-A26:41	HLA-B35:77
DRB1_1544	HLA-A26:42	HLA-B35:78
DRB1_1547	HLA-A26:43	HLA-B35:79
DRB1_0407	HLA-A26:45	HLA-B35:80
DRB1_0414	HLA-A26:46	HLA-B35:81
DRB1_0454	HLA-A26:47	HLA-B35:82
DRB1_0469	HLA-A26:48	HLA-B35:83
DRB1_0474	HLA-A26:49	HLA-B35:84
DRB1_0482	HLA-A26:50	HLA-B35:85
DRB1_0488	HLA-A29:01	HLA-B35:86
DRB1_0815	HLA-A29:02	HLA-B35:87
DRB1_1347	HLA-A29:03	HLA-B35:88
DRB1_1410	HLA-A29:04	HLA-B35:89
DRB1_1427	HLA-A29:05	HLA-B35:90
DRB1_1493	HLA-A29:06	HLA-B35:91
DRB1_1498	HLA-A29:07	HLA-B35:92
DRB1_1538	HLA-A29:09	HLA-B35:93
DRB1_1604	HLA-A29:10	HLA-B35:94
DRB3_0217	HLA-A29:11	HLA-B35:95
	HLA-A29:12	HLA-B35:96
	HLA-A29:13	HLA-B35:97
	HLA-A29:14	HLA-B35:98
	HLA-A29:15	HLA-B35:99
	HLA-A29:16	HLA-B37:01
	HLA-A29:17	HLA-B37:02
	HLA-A29:18	HLA-B37:04
	HLA-A29:19	HLA-B37:05
	HLA-A29:20	HLA-B37:06
	HLA-A29:21	HLA-B37:07
	HLA-A29:22	HLA-B37:08
	HLA-A30:01	HLA-B37:09
	HLA-A30:02	HLA-B37:10
	HLA-A30:03	HLA-B37:11
	HLA-A30:04	HLA-B37:12
	HLA-A30:06	HLA-B37:13
	HLA-A30:07	HLA-B37:14

HLA-A30:08	HLA-B37:15
HLA-A30:09	HLA-B37:17
HLA-A30:10	HLA-B37:18
HLA-A30:11	HLA-B37:19
HLA-A30:12	HLA-B37:20
HLA-A30:13	HLA-B37:21
HLA-A30:15	HLA-B37:22
HLA-A30:16	HLA-B37:23
HLA-A30:17	HLA-B38:01
HLA-A30:18	HLA-B38:02
HLA-A30:19	HLA-B38:03
HLA-A30:20	HLA-B38:04
HLA-A30:22	HLA-B38:05
HLA-A30:23	HLA-B38:06
HLA-A30:24	HLA-B38:07
HLA-A30:25	HLA-B38:08
HLA-A30:26	HLA-B38:09
HLA-A30:28	HLA-B38:10
HLA-A30:29	HLA-B38:11
HLA-A30:30	HLA-B38:12
HLA-A30:31	HLA-B38:13
HLA-A30:32	HLA-B38:14
HLA-A30:33	HLA-B38:15
HLA-A30:34	HLA-B38:16
HLA-A30:35	HLA-B38:17
HLA-A30:36	HLA-B38:18
HLA-A30:37	HLA-B38:19
HLA-A30:38	HLA-B38:20
HLA-A30:39	HLA-B38:21
HLA-A30:40	HLA-B38:22
HLA-A30:41	HLA-B38:23
HLA-A31:01	HLA-B39:01
HLA-A31:02	HLA-B39:02
HLA-A31:03	HLA-B39:03
HLA-A31:04	HLA-B39:04
HLA-A31:05	HLA-B39:05
HLA-A31:06	HLA-B39:06
HLA-A31:07	HLA-B39:07
HLA-A31:08	HLA-B39:08
HLA-A31:09	HLA-B39:09
HLA-A31:10	HLA-B39:10
HLA-A31:11	HLA-B39:11

HLA-A31:12	HLA-B39:12
HLA-A31:13	HLA-B39:13
HLA-A31:15	HLA-B39:14
HLA-A31:16	HLA-B39:15
HLA-A31:17	HLA-B39:16
HLA-A31:18	HLA-B39:17
HLA-A31:19	HLA-B39:18
HLA-A31:20	HLA-B39:19
HLA-A31:21	HLA-B39:20
HLA-A31:22	HLA-B39:22
HLA-A31:23	HLA-B39:23
HLA-A31:24	HLA-B39:24
HLA-A31:25	HLA-B39:26
HLA-A31:26	HLA-B39:27
HLA-A31:27	HLA-B39:28
HLA-A31:28	HLA-B39:29
HLA-A31:29	HLA-B39:30
HLA-A31:30	HLA-B39:31
HLA-A31:31	HLA-B39:32
HLA-A31:32	HLA-B39:33
HLA-A31:33	HLA-B39:34
HLA-A31:34	HLA-B39:35
HLA-A31:35	HLA-B39:36
HLA-A31:36	HLA-B39:37
HLA-A31:37	HLA-B39:39
HLA-A32:01	HLA-B39:41
HLA-A32:02	HLA-B39:42
HLA-A32:03	HLA-B39:43
HLA-A32:04	HLA-B39:44
HLA-A32:05	HLA-B39:45
HLA-A32:06	HLA-B39:46
HLA-A32:07	HLA-B39:47
HLA-A32:08	HLA-B39:48
HLA-A32:09	HLA-B39:49
HLA-A32:10	HLA-B39:50
HLA-A32:12	HLA-B39:51
HLA-A32:13	HLA-B39:52
HLA-A32:14	HLA-B39:53
HLA-A32:15	HLA-B39:54
HLA-A32:16	HLA-B39:55
HLA-A32:17	HLA-B39:56
HLA-A32:18	HLA-B39:57

HLA-A32:20
HLA-A32:21
HLA-A32:22
HLA-A32:23
HLA-A32:24
HLA-A32:25
HLA-A33:01
HLA-A33:03
HLA-A33:04
HLA-A33:05
HLA-A33:06
HLA-A33:07
HLA-A33:08
HLA-A33:09
HLA-A33:10
HLA-A33:11
HLA-A33:12
HLA-A33:13
HLA-A33:14
HLA-A33:15
HLA-A33:16
HLA-A33:17
HLA-A33:18
HLA-A33:19
HLA-A33:20
HLA-A33:21
HLA-A33:22
HLA-A33:23
HLA-A33:24
HLA-A33:25
HLA-A33:26
HLA-A33:27
HLA-A33:28
HLA-A33:29
HLA-A33:30
HLA-A33:31
HLA-A34:01
HLA-A34:02
HLA-A34:03
HLA-A34:04
HLA-A34:05
HLA-A34:06
HLA-B39:58
HLA-B39:59
HLA-B39:60
HLA-B40:01
HLA-B40:02
HLA-B40:03
HLA-B40:04
HLA-B40:05
HLA-B40:06
HLA-B40:07
HLA-B40:08
HLA-B40:09
HLA-B40:10
HLA-B40:100
HLA-B40:101
HLA-B40:102
HLA-B40:103
HLA-B40:104
HLA-B40:105
HLA-B40:106
HLA-B40:107
HLA-B40:108
HLA-B40:109
HLA-B40:11
HLA-B40:110
HLA-B40:111
HLA-B40:112
HLA-B40:113
HLA-B40:114
HLA-B40:115
HLA-B40:116
HLA-B40:117
HLA-B40:119
HLA-B40:12
HLA-B40:120
HLA-B40:121
HLA-B40:122
HLA-B40:123
HLA-B40:124
HLA-B40:125
HLA-B40:126
HLA-B40:127

HLA-A34:07	HLA-B40:128
HLA-A34:08	HLA-B40:129
HLA-A36:01	HLA-B40:13
HLA-A36:02	HLA-B40:130
HLA-A36:03	HLA-B40:131
HLA-A36:04	HLA-B40:132
HLA-A36:05	HLA-B40:134
HLA-A43:01	HLA-B40:135
HLA-A66:01	HLA-B40:136
HLA-A66:02	HLA-B40:137
HLA-A66:03	HLA-B40:138
HLA-A66:04	HLA-B40:139
HLA-A66:05	HLA-B40:14
HLA-A66:06	HLA-B40:140
HLA-A66:07	HLA-B40:141
HLA-A66:08	HLA-B40:143
HLA-A66:09	HLA-B40:145
HLA-A66:10	HLA-B40:146
HLA-A66:11	HLA-B40:147
HLA-A66:12	HLA-B40:15
HLA-A66:13	HLA-B40:16
HLA-A66:14	HLA-B40:18
HLA-A66:15	HLA-B40:19
HLA-A68:01	HLA-B40:20
HLA-A68:02	HLA-B40:21
HLA-A68:03	HLA-B40:23
HLA-A68:04	HLA-B40:24
HLA-A68:05	HLA-B40:25
HLA-A68:06	HLA-B40:26
HLA-A68:07	HLA-B40:27
HLA-A68:08	HLA-B40:28
HLA-A68:09	HLA-B40:29
HLA-A68:10	HLA-B40:30
HLA-A68:12	HLA-B40:31
HLA-A68:13	HLA-B40:32
HLA-A68:14	HLA-B40:33
HLA-A68:15	HLA-B40:34
HLA-A68:16	HLA-B40:35
HLA-A68:17	HLA-B40:36
HLA-A68:19	HLA-B40:37
HLA-A68:20	HLA-B40:38
HLA-A68:21	HLA-B40:39

HLA-A68:22	HLA-B40:40
HLA-A68:23	HLA-B40:42
HLA-A68:24	HLA-B40:43
HLA-A68:25	HLA-B40:44
HLA-A68:26	HLA-B40:45
HLA-A68:27	HLA-B40:46
HLA-A68:28	HLA-B40:47
HLA-A68:29	HLA-B40:48
HLA-A68:30	HLA-B40:49
HLA-A68:31	HLA-B40:50
HLA-A68:32	HLA-B40:51
HLA-A68:33	HLA-B40:52
HLA-A68:34	HLA-B40:53
HLA-A68:35	HLA-B40:54
HLA-A68:36	HLA-B40:55
HLA-A68:37	HLA-B40:56
HLA-A68:38	HLA-B40:57
HLA-A68:39	HLA-B40:58
HLA-A68:40	HLA-B40:59
HLA-A68:41	HLA-B40:60
HLA-A68:42	HLA-B40:61
HLA-A68:43	HLA-B40:62
HLA-A68:44	HLA-B40:63
HLA-A68:45	HLA-B40:64
HLA-A68:46	HLA-B40:65
HLA-A68:47	HLA-B40:66
HLA-A68:48	HLA-B40:67
HLA-A68:50	HLA-B40:68
HLA-A68:51	HLA-B40:69
HLA-A68:52	HLA-B40:70
HLA-A68:53	HLA-B40:71
HLA-A68:54	HLA-B40:72
HLA-A69:01	HLA-B40:73
HLA-A74:01	HLA-B40:74
HLA-A74:02	HLA-B40:75
HLA-A74:03	HLA-B40:76
HLA-A74:04	HLA-B40:77
HLA-A74:05	HLA-B40:78
HLA-A74:06	HLA-B40:79
HLA-A74:07	HLA-B40:80
HLA-A74:08	HLA-B40:81
HLA-A74:09	HLA-B40:82

HLA-A74:10
HLA-A74:11
HLA-A74:13
HLA-A80:01
HLA-A80:02

HLA-B40:83
HLA-B40:84
HLA-B40:85
HLA-B40:86
HLA-B40:87
HLA-B40:88
HLA-B40:89
HLA-B40:90
HLA-B40:91
HLA-B40:92
HLA-B40:93
HLA-B40:94
HLA-B40:95
HLA-B40:96
HLA-B40:97
HLA-B40:98
HLA-B40:99
HLA-B41:01
HLA-B41:02
HLA-B41:03
HLA-B41:04
HLA-B41:05
HLA-B41:06
HLA-B41:07
HLA-B41:08
HLA-B41:09
HLA-B41:10
HLA-B41:11
HLA-B41:12
HLA-B42:01
HLA-B42:02
HLA-B42:04
HLA-B42:05
HLA-B42:06
HLA-B42:07
HLA-B42:08
HLA-B42:09
HLA-B42:10
HLA-B42:11
HLA-B42:12
HLA-B42:13
HLA-B42:14

HLA-B44:02
HLA-B44:03
HLA-B44:04
HLA-B44:05
HLA-B44:06
HLA-B44:07
HLA-B44:08
HLA-B44:09
HLA-B44:10
HLA-B44:100
HLA-B44:101
HLA-B44:102
HLA-B44:103
HLA-B44:104
HLA-B44:105
HLA-B44:106
HLA-B44:107
HLA-B44:109
HLA-B44:11
HLA-B44:110
HLA-B44:12
HLA-B44:13
HLA-B44:14
HLA-B44:15
HLA-B44:16
HLA-B44:17
HLA-B44:18
HLA-B44:20
HLA-B44:21
HLA-B44:22
HLA-B44:24
HLA-B44:25
HLA-B44:26
HLA-B44:27
HLA-B44:28
HLA-B44:29
HLA-B44:30
HLA-B44:31
HLA-B44:32
HLA-B44:33
HLA-B44:34
HLA-B44:35

HLA-B44:36
HLA-B44:37
HLA-B44:38
HLA-B44:39
HLA-B44:40
HLA-B44:41
HLA-B44:42
HLA-B44:43
HLA-B44:44
HLA-B44:45
HLA-B44:46
HLA-B44:47
HLA-B44:48
HLA-B44:49
HLA-B44:50
HLA-B44:51
HLA-B44:53
HLA-B44:54
HLA-B44:55
HLA-B44:57
HLA-B44:59
HLA-B44:60
HLA-B44:62
HLA-B44:63
HLA-B44:64
HLA-B44:65
HLA-B44:66
HLA-B44:67
HLA-B44:68
HLA-B44:69
HLA-B44:70
HLA-B44:71
HLA-B44:72
HLA-B44:73
HLA-B44:74
HLA-B44:75
HLA-B44:76
HLA-B44:77
HLA-B44:78
HLA-B44:79
HLA-B44:80
HLA-B44:81

HLA-B44:82
HLA-B44:83
HLA-B44:84
HLA-B44:85
HLA-B44:86
HLA-B44:87
HLA-B44:88
HLA-B44:89
HLA-B44:90
HLA-B44:91
HLA-B44:92
HLA-B44:93
HLA-B44:94
HLA-B44:95
HLA-B44:96
HLA-B44:97
HLA-B44:98
HLA-B44:99
HLA-B45:01
HLA-B45:02
HLA-B45:03
HLA-B45:04
HLA-B45:05
HLA-B45:06
HLA-B45:07
HLA-B45:08
HLA-B45:09
HLA-B45:10
HLA-B45:11
HLA-B45:12
HLA-B46:01
HLA-B46:02
HLA-B46:03
HLA-B46:04
HLA-B46:05
HLA-B46:06
HLA-B46:08
HLA-B46:09
HLA-B46:10
HLA-B46:11
HLA-B46:12
HLA-B46:13

HLA-B46:14
HLA-B46:16
HLA-B46:17
HLA-B46:18
HLA-B46:19
HLA-B46:20
HLA-B46:21
HLA-B46:22
HLA-B46:23
HLA-B46:24
HLA-B47:01
HLA-B47:02
HLA-B47:03
HLA-B47:04
HLA-B47:05
HLA-B47:06
HLA-B47:07
HLA-B48:01
HLA-B48:02
HLA-B48:03
HLA-B48:04
HLA-B48:05
HLA-B48:06
HLA-B48:07
HLA-B48:08
HLA-B48:09
HLA-B48:10
HLA-B48:11
HLA-B48:12
HLA-B48:13
HLA-B48:14
HLA-B48:15
HLA-B48:16
HLA-B48:17
HLA-B48:18
HLA-B48:19
HLA-B48:20
HLA-B48:21
HLA-B48:22
HLA-B48:23
HLA-B49:01
HLA-B49:02

HLA-B49:03
HLA-B49:04
HLA-B49:05
HLA-B49:06
HLA-B49:07
HLA-B49:08
HLA-B49:09
HLA-B49:10
HLA-B50:01
HLA-B50:02
HLA-B50:04
HLA-B50:05
HLA-B50:06
HLA-B50:07
HLA-B50:08
HLA-B50:09
HLA-B51:01
HLA-B51:02
HLA-B51:03
HLA-B51:04
HLA-B51:05
HLA-B51:06
HLA-B51:07
HLA-B51:08
HLA-B51:09
HLA-B51:12
HLA-B51:13
HLA-B51:14
HLA-B51:15
HLA-B51:16
HLA-B51:17
HLA-B51:18
HLA-B51:19
HLA-B51:20
HLA-B51:21
HLA-B51:22
HLA-B51:23
HLA-B51:24
HLA-B51:26
HLA-B51:28
HLA-B51:29
HLA-B51:30

HLA-B51:31
HLA-B51:32
HLA-B51:33
HLA-B51:34
HLA-B51:35
HLA-B51:36
HLA-B51:37
HLA-B51:38
HLA-B51:39
HLA-B51:40
HLA-B51:42
HLA-B51:43
HLA-B51:45
HLA-B51:46
HLA-B51:48
HLA-B51:49
HLA-B51:50
HLA-B51:51
HLA-B51:52
HLA-B51:53
HLA-B51:54
HLA-B51:55
HLA-B51:56
HLA-B51:57
HLA-B51:58
HLA-B51:59
HLA-B51:60
HLA-B51:61
HLA-B51:62
HLA-B51:63
HLA-B51:64
HLA-B51:65
HLA-B51:66
HLA-B51:67
HLA-B51:68
HLA-B51:69
HLA-B51:70
HLA-B51:71
HLA-B51:72
HLA-B51:73
HLA-B51:74
HLA-B51:75

HLA-B51:76
HLA-B51:77
HLA-B51:78
HLA-B51:79
HLA-B51:80
HLA-B51:81
HLA-B51:82
HLA-B51:83
HLA-B51:84
HLA-B51:85
HLA-B51:86
HLA-B51:87
HLA-B51:88
HLA-B51:89
HLA-B51:90
HLA-B51:91
HLA-B51:92
HLA-B51:93
HLA-B51:94
HLA-B51:95
HLA-B51:96
HLA-B52:01
HLA-B52:02
HLA-B52:03
HLA-B52:04
HLA-B52:05
HLA-B52:06
HLA-B52:07
HLA-B52:08
HLA-B52:09
HLA-B52:10
HLA-B52:11
HLA-B52:12
HLA-B52:13
HLA-B52:14
HLA-B52:15
HLA-B52:16
HLA-B52:17
HLA-B52:18
HLA-B52:19
HLA-B52:20
HLA-B52:21

HLA-B53:01
HLA-B53:02
HLA-B53:03
HLA-B53:04
HLA-B53:05
HLA-B53:06
HLA-B53:07
HLA-B53:08
HLA-B53:09
HLA-B53:10
HLA-B53:11
HLA-B53:12
HLA-B53:13
HLA-B53:14
HLA-B53:15
HLA-B53:16
HLA-B53:17
HLA-B53:18
HLA-B53:19
HLA-B53:20
HLA-B53:21
HLA-B53:22
HLA-B53:23
HLA-B54:01
HLA-B54:02
HLA-B54:03
HLA-B54:04
HLA-B54:06
HLA-B54:07
HLA-B54:09
HLA-B54:10
HLA-B54:11
HLA-B54:12
HLA-B54:13
HLA-B54:14
HLA-B54:15
HLA-B54:16
HLA-B54:17
HLA-B54:18
HLA-B54:19
HLA-B54:20
HLA-B54:21

HLA-B54:22
HLA-B54:23
HLA-B55:01
HLA-B55:02
HLA-B55:03
HLA-B55:04
HLA-B55:05
HLA-B55:07
HLA-B55:08
HLA-B55:09
HLA-B55:10
HLA-B55:11
HLA-B55:12
HLA-B55:13
HLA-B55:14
HLA-B55:15
HLA-B55:16
HLA-B55:17
HLA-B55:18
HLA-B55:19
HLA-B55:20
HLA-B55:21
HLA-B55:22
HLA-B55:23
HLA-B55:24
HLA-B55:25
HLA-B55:26
HLA-B55:27
HLA-B55:28
HLA-B55:29
HLA-B55:30
HLA-B55:31
HLA-B55:32
HLA-B55:33
HLA-B55:34
HLA-B55:35
HLA-B55:36
HLA-B55:37
HLA-B55:38
HLA-B55:39
HLA-B55:40
HLA-B55:41

HLA-B55:42
HLA-B55:43
HLA-B56:01
HLA-B56:02
HLA-B56:03
HLA-B56:04
HLA-B56:05
HLA-B56:06
HLA-B56:07
HLA-B56:08
HLA-B56:09
HLA-B56:10
HLA-B56:11
HLA-B56:12
HLA-B56:13
HLA-B56:14
HLA-B56:15
HLA-B56:16
HLA-B56:17
HLA-B56:18
HLA-B56:20
HLA-B56:21
HLA-B56:22
HLA-B56:23
HLA-B56:24
HLA-B56:25
HLA-B56:26
HLA-B56:27
HLA-B56:29
HLA-B57:01
HLA-B57:02
HLA-B57:03
HLA-B57:04
HLA-B57:05
HLA-B57:06
HLA-B57:07
HLA-B57:08
HLA-B57:09
HLA-B57:10
HLA-B57:11
HLA-B57:12
HLA-B57:13

HLA-B57:14
HLA-B57:15
HLA-B57:16
HLA-B57:17
HLA-B57:18
HLA-B57:19
HLA-B57:20
HLA-B57:21
HLA-B57:22
HLA-B57:23
HLA-B57:24
HLA-B57:25
HLA-B57:26
HLA-B57:27
HLA-B57:29
HLA-B57:30
HLA-B57:31
HLA-B57:32
HLA-B58:01
HLA-B58:02
HLA-B58:04
HLA-B58:05
HLA-B58:06
HLA-B58:07
HLA-B58:08
HLA-B58:09
HLA-B58:11
HLA-B58:12
HLA-B58:13
HLA-B58:14
HLA-B58:15
HLA-B58:16
HLA-B58:18
HLA-B58:19
HLA-B58:20
HLA-B58:21
HLA-B58:22
HLA-B58:23
HLA-B58:24
HLA-B58:25
HLA-B58:26
HLA-B58:27

HLA-B58:28
HLA-B58:29
HLA-B58:30
HLA-B59:01
HLA-B59:02
HLA-B59:03
HLA-B59:04
HLA-B59:05
HLA-B67:01
HLA-B67:02
HLA-B73:01
HLA-B73:02
HLA-B78:01
HLA-B78:02
HLA-B78:03
HLA-B78:04
HLA-B78:05
HLA-B78:06
HLA-B78:07
HLA-B81:01
HLA-B81:02
HLA-B81:03
HLA-B81:05
HLA-B82:01
HLA-B82:02
HLA-B82:03
HLA-B83:01
