# Supplementary material for: Equivalent T Cell Epitope Promiscuity in Ecologically Diverse Human Pathogens
Source: PLoS One. 2013 Aug 9;8(8):e73124. doi: 10.1371/journal.pone.0073124 (PMC3739752; doi:10.1371/journal.pone.0073124)
Supplement: Table S3 — (PDF) [file pone.0073124.s006.pdf]

Europe and the Americas	East Asia	Sub-Saharan Africa	India and East Africa	Oceania
HLA-A01:01	HLA-A02:01	HLA-A01:01	HLA-A01:01	HLA-A11:01
HLA-A02:01	HLA-A02:06	HLA-A02:01	HLA-A02:01	HLA-A24:02
HLA-A24:02	HLA-A02:07	HLA-A02:02	HLA-A03:01	HLA-A34:01
HLA-A31:01	HLA-A11:01	HLA-A03:01	HLA-A11:01	
	HLA-A24:02	HLA-A23:01	HLA-A24:02	
	HLA-A33:03	HLA-A29:02	HLA-A26:01	
		HLA-A30:01	HLA-A32:01	
		HLA-A30:02	HLA-A33:03	
		HLA-A68:02		
HLA-B40:02	HLA-B40:01	HLA-B53:01	HLA-B40:06	HLA-B40:01
HLA-B35:01	HLA-B46:01	HLA-B15:03	HLA-B51:01	HLA-B56:02
HLA-B07:02	HLA-B58:01	HLA-B42:01	HLA-B52:01	HLA-B15:02
HLA-B08:01	HLA-B40:02	HLA-B58:02	HLA-B08:01	HLA-B40:02
HLA-B39:06	HLA-B51:01	HLA-B58:01	HLA-B35:03	HLA-B38:02
HLA-B44:02	HLA-B13:01	HLA-B45:01	HLA-B35:01	HLA-B56:01
HLA-B27:05	HLA-B15:01	HLA-B35:01	HLA-B58:01	HLA-B35:05
HLA-B15:04	HLA-B35:01	HLA-B15:10	HLA-B18:01	HLA-B13:01
HLA-B35:05	HLA-B48:01	HLA-B18:01	HLA-B44:03	HLA-B48:01
HLA-B51:01	HLA-B39:01	HLA-B08:01	HLA-B51:01	HLA-B15:06
HLA-B48:01	HLA-B44:03	HLA-B07:02	HLA-B50:01	
HLA-B39:05	HLA-B15:02		HLA-B57:01	
HLA-B15:01	HLA-B54:01		HLA-B35:08	
HLA-B40:04			HLA-B55:01	
			HLA-B37:01	
HLA-DRB1*03:01	HLA-DRB1*04:03	HLA-DRB1*01:02	HLA-DRB1*01:01	HLA-DRB1*11:01
HLA-DRB1*04:01	HLA-DRB1*04:05	HLA-DRB1*03:01	HLA-DRB1*03:01	HLA-DRB1*12:02
HLA-DRB1*04:07	HLA-DRB1*07:01	HLA-DRB1*03:02	HLA-DRB1*04:03	HLA-DRB1*15:01
HLA-DRB1*04:11	HLA-DRB1*08:03	HLA-DRB1*07:01	HLA-DRB1*07:01	HLA-DRB1*15:02
HLA-DRB1*07:01	HLA-DRB1*09:01	HLA-DRB1*11:01	HLA-DRB1*10:01	HLA-DRB1*16:02
HLA-DRB1*08:02	HLA-DRB1*11:01	HLA-DRB1*11:02	HLA-DRB1*11:01	
HLA-DRB1*08:07	HLA-DRB1*12:01	HLA-DRB1*13:01	HLA-DRB1*11:04	
HLA-DRB1*14:02	HLA-DRB1*12:02	HLA-DRB1*13:02	HLA-DRB1*13:01	
HLA-DRB1*15:01	HLA-DRB1*13:02	HLA-DRB1*15:03	HLA-DRB1*14:01	
HLA-DRB1*16:02	HLA-DRB1*14:01		HLA-DRB1*15:01	
	HLA-DRB1*15:01			
	HLA-DRB1*16:02			
