# Supplementary material for: Equivalent T Cell Epitope Promiscuity in Ecologically Diverse Human Pathogens
Source: PLoS One. 2013 Aug 9;8(8):e73124. doi: 10.1371/journal.pone.0073124 (PMC3739752; doi:10.1371/journal.pone.0073124)
Supplement: Table S4 — (PDF) [file pone.0073124.s007.pdf]

A01	A02	A03	A24	B07	B08	B27	B44	B58	B62
HLA-A01:01	HLA-A02:01	HLA-A02:65	HLA-A23:01	HLA-B07:02	HLA-B08:01	HLA-B14:01	HLA-B15:46	HLA-B15:16	HLA-B13:09
HLA-A01:03	HLA-A02:02	HLA-A02:80	HLA-A23:02	HLA-B07:03	HLA-B08:02	HLA-B14:02	HLA-B15:53	HLA-B15:17	HLA-B13:13
HLA-A01:06	HLA-A02:03	HLA-A03:01	HLA-A23:03	HLA-B07:04	HLA-B08:03	HLA-B14:03	HLA-B18:01	HLA-B15:67	HLA-B15:01
HLA-A01:07	HLA-A02:04	HLA-A03:02	HLA-A23:04	HLA-B07:05	HLA-B08:07	HLA-B14:05	HLA-B18:02	HLA-B15:95	HLA-B15:02
HLA-A01:08	HLA-A02:05	HLA-A03:04	HLA-A23:05	HLA-B07:06	HLA-B08:09	HLA-B14:06	HLA-B18:03	HLA-B57:01	HLA-B15:04
HLA-A01:09	HLA-A02:06	HLA-A03:05	HLA-A23:06	HLA-B07:07	HLA-B08:11	HLA-B15:03	HLA-B18:05	HLA-B57:02	HLA-B15:05
HLA-A01:10	HLA-A02:07	HLA-A03:06	HLA-A23:10	HLA-B07:09	HLA-B08:12	HLA-B15:09	HLA-B18:06	HLA-B57:03	HLA-B15:07
HLA-A01:12	HLA-A02:09	HLA-A03:07	HLA-A23:12	HLA-B07:12	HLA-B08:13	HLA-B15:10	HLA-B18:10	HLA-B57:04	HLA-B15:12
HLA-A01:14	HLA-A02:11	HLA-A03:08	HLA-A24:02	HLA-B07:14	HLA-B08:15	HLA-B15:18	HLA-B18:11	HLA-B57:05	HLA-B15:13
HLA-A25:01	HLA-A02:12	HLA-A03:10	HLA-A24:03	HLA-B07:16	HLA-B08:16	HLA-B15:23	HLA-B18:13	HLA-B57:06	HLA-B15:14
HLA-A25:02	HLA-A02:13	HLA-A03:12	HLA-A24:05	HLA-B07:17	HLA-B08:18	HLA-B15:37	HLA-B18:14	HLA-B57:07	HLA-B15:15
HLA-A25:04	HLA-A02:16	HLA-A03:13	HLA-A24:06	HLA-B07:18	HLA-B08:20	HLA-B15:47	HLA-B18:15	HLA-B57:08	HLA-B15:19
HLA-A26:01	HLA-A02:18	HLA-A03:14	HLA-A24:08	HLA-B07:19	HLA-B08:21	HLA-B15:49	HLA-B18:19	HLA-B57:09	HLA-B15:20
HLA-A26:02	HLA-A02:19	HLA-A03:16	HLA-A24:10	HLA-B07:20	HLA-B08:22	HLA-B15:51	HLA-B18:20	HLA-B58:01	HLA-B15:24
HLA-A26:03	HLA-A02:20	HLA-A03:17	HLA-A24:13	HLA-B07:21	HLA-B08:23	HLA-B15:52	HLA-B37:04	HLA-B58:02	HLA-B15:25
HLA-A26:04	HLA-A02:21	HLA-A11:01	HLA-A24:17	HLA-B07:22	HLA-B08:24	HLA-B15:54	HLA-B40:01	HLA-B58:04	HLA-B15:28
HLA-A26:05	HLA-A02:22	HLA-A11:02	HLA-A24:18	HLA-B07:23	HLA-B08:25	HLA-B15:61	HLA-B40:02	HLA-B58:05	HLA-B15:30
HLA-A26:06	HLA-A02:24	HLA-A11:03	HLA-A24:20	HLA-B07:24		HLA-B15:62	HLA-B40:03	HLA-B58:06	HLA-B15:31
HLA-A26:07	HLA-A02:25	HLA-A11:04	HLA-A24:21	HLA-B07:25		HLA-B15:68	HLA-B40:04	HLA-B58:07	HLA-B15:33
HLA-A26:08	HLA-A02:26	HLA-A11:05	HLA-A24:22	HLA-B07:26		HLA-B15:69	HLA-B40:05	HLA-B58:08	HLA-B15:34
HLA-A26:09	HLA-A02:27	HLA-A11:06	HLA-A24:23	HLA-B07:31		HLA-B15:72	HLA-B40:06	HLA-B58:09	HLA-B15:35
HLA-A26:10	HLA-A02:28	HLA-A11:07	HLA-A24:25	HLA-B07:33		HLA-B15:74	HLA-B40:09	HLA-B58:11	HLA-B15:38
HLA-A26:12	HLA-A02:30	HLA-A11:08	HLA-A24:26	HLA-B07:34		HLA-B15:80	HLA-B40:10		HLA-B15:39
HLA-A26:13	HLA-A02:31	HLA-A11:09	HLA-A24:27	HLA-B07:35		HLA-B15:90	HLA-B40:11		HLA-B15:40
HLA-A26:14	HLA-A02:36	HLA-A11:10	HLA-A24:28	HLA-B07:36		HLA-B15:91	HLA-B40:14		HLA-B15:42
HLA-A26:15	HLA-A02:37	HLA-A11:12	HLA-A24:29	HLA-B07:37		HLA-B15:93	HLA-B40:15		HLA-B15:45
HLA-A26:17	HLA-A02:38	HLA-A11:13	HLA-A24:30	HLA-B07:39		HLA-B15:98	HLA-B40:16		HLA-B15:48
HLA-A26:18	HLA-A02:39	HLA-A11:14	HLA-A24:33	HLA-B07:40		HLA-B15:99	HLA-B40:18		HLA-B15:50
HLA-A26:19	HLA-A02:40	HLA-A11:15	HLA-A24:34	HLA-B07:41		HLA-B27:01	HLA-B40:19		HLA-B15:55
HLA-A26:21	HLA-A02:41	HLA-A11:16	HLA-A24:35	HLA-B07:42		HLA-B27:02	HLA-B40:20		HLA-B15:58
HLA-A26:22	HLA-A02:42	HLA-A11:20	HLA-A24:37	HLA-B07:43		HLA-B27:03	HLA-B40:23		HLA-B15:60
HLA-A26:23	HLA-A02:44	HLA-A11:22	HLA-A24:38	HLA-B35:01		HLA-B27:04	HLA-B40:24		HLA-B15:63
HLA-A26:24	HLA-A02:45	HLA-A11:23	HLA-A24:39	HLA-B35:02		HLA-B27:05	HLA-B40:26		HLA-B15:65
HLA-A26:26	HLA-A02:46	HLA-A31:01	HLA-A24:41	HLA-B35:03		HLA-B27:06	HLA-B40:28		HLA-B15:70
HLA-A30:02	HLA-A02:47	HLA-A31:03	HLA-A24:42	HLA-B35:04		HLA-B27:07	HLA-B40:29		HLA-B15:73
HLA-A30:03	HLA-A02:48	HLA-A31:04	HLA-A24:43	HLA-B35:05		HLA-B27:09	HLA-B40:30		HLA-B15:75
HLA-A30:04	HLA-A02:49	HLA-A31:05	HLA-A24:44	HLA-B35:06		HLA-B27:10	HLA-B40:33		HLA-B15:78
HLA-A30:06	HLA-A02:50	HLA-A31:06	HLA-A24:46	HLA-B35:07		HLA-B27:11	HLA-B40:34		HLA-B15:81
HLA-A30:09	HLA-A02:51	HLA-A31:09	HLA-A24:47	HLA-B35:08		HLA-B27:13	HLA-B40:35		HLA-B15:82
HLA-A30:12	HLA-A02:54	HLA-A31:11	HLA-A24:49	HLA-B35:09		HLA-B27:14	HLA-B40:36		HLA-B15:83
HLA-A31:10	HLA-A02:56	HLA-A31:12	HLA-A24:52	HLA-B35:11		HLA-B27:15	HLA-B40:38		HLA-B15:85
HLA-A32:01	HLA-A02:57	HLA-A33:01	HLA-A29:01	HLA-B35:12		HLA-B27:17	HLA-B40:39		HLA-B15:86
HLA-A32:02	HLA-A02:58	HLA-A33:03	HLA-A29:02	HLA-B35:14		HLA-B27:19	HLA-B40:40		HLA-B15:88
HLA-A32:03	HLA-A02:59	HLA-A33:04	HLA-A29:03	HLA-B35:15		HLA-B27:20	HLA-B40:42		HLA-B15:92
HLA-A32:04	HLA-A02:60	HLA-A33:05	HLA-A29:05	HLA-B35:17		HLA-B27:21	HLA-B40:43		HLA-B15:96
HLA-A32:05	HLA-A02:61	HLA-A33:06	HLA-A29:06	HLA-B35:18		HLA-B27:24	HLA-B40:44		HLA-B15:97
HLA-A32:06	HLA-A02:62	HLA-A33:07	HLA-A29:09	HLA-B35:21		HLA-B27:25	HLA-B40:45		HLA-B35:28
HLA-A32:07	HLA-A02:63	HLA-A34:02	HLA-A29:10	HLA-B35:22		HLA-B27:27	HLA-B40:47		HLA-B40:21
HLA-A32:08	HLA-A02:66	HLA-A34:03	HLA-A29:11	HLA-B35:24		HLA-B27:28	HLA-B40:48		HLA-B44:08
HLA-A32:09	HLA-A02:67	HLA-A34:04	HLA-A29:12	HLA-B35:29		HLA-B27:30	HLA-B40:49		HLA-B46:01
HLA-A32:10	HLA-A02:68	HLA-A34:06	HLA-A29:13	HLA-B35:30		HLA-B38:01	HLA-B40:50		HLA-B46:02
HLA-A36:03	HLA-A02:69	HLA-A66:01		HLA-B35:31		HLA-B38:05	HLA-B40:51		HLA-B46:03
HLA-A74:10	HLA-A02:70	HLA-A66:02		HLA-B35:32		HLA-B38:09	HLA-B40:52		HLA-B46:04
HLA-A80:01	HLA-A02:71	HLA-A66:03		HLA-B35:33		HLA-B38:10	HLA-B40:53		HLA-B46:05
HLA-A02:52	HLA-A02:72	HLA-A66:04		HLA-B35:34		HLA-B38:11	HLA-B40:54		HLA-B52:01
HLA-A30:01	HLA-A02:73	HLA-A68:01		HLA-B35:35		HLA-B39:01	HLA-B40:55		HLA-B52:02
HLA-A30:08	HLA-A02:74	HLA-A68:03		HLA-B35:36		HLA-B39:02	HLA-B40:56		HLA-B52:03
HLA-A30:11	HLA-A02:75	HLA-A68:04		HLA-B35:37		HLA-B39:03	HLA-B40:57		HLA-B52:04
HLA-A30:13	HLA-A02:77	HLA-A68:05		HLA-B35:38		HLA-B39:04	HLA-B40:58		HLA-B52:05
HLA-A30:15	HLA-A02:78	HLA-A68:08		HLA-B35:39		HLA-B39:05	HLA-B40:59		HLA-B52:07
HLA-A68:06	HLA-A02:79	HLA-A68:09		HLA-B35:41		HLA-B39:06	HLA-B41:02		HLA-B52:08

HLA-A68:07	HLA-A02:84	HLA-A68:10	HLA-B35:42	HLA-B39:07	HLA-B41:03	HLA-B78:05
HLA-A29:01	HLA-A02:85	HLA-A68:12	HLA-B35:43	HLA-B39:08	HLA-B41:04	
HLA-A29:02	HLA-A02:86	HLA-A68:13	HLA-B35:44	HLA-B39:09	HLA-B41:06	
HLA-A29:03	HLA-A68:02	HLA-A68:14	HLA-B35:45	HLA-B39:11	HLA-B41:07	
HLA-A29:05	HLA-A68:15	HLA-A68:16	HLA-B35:46	HLA-B39:13	HLA-B44:02	
HLA-A29:06	HLA-A68:27	HLA-A68:19	HLA-B35:51	HLA-B39:14	HLA-B44:03	
HLA-A29:09	HLA-A68:28	HLA-A68:20	HLA-B35:54	HLA-B39:15	HLA-B44:04	
HLA-A29:10	HLA-A69:01	HLA-A68:21	HLA-B35:55	HLA-B39:18	HLA-B44:05	
HLA-A29:11		HLA-A68:22	HLA-B35:57	HLA-B39:23	HLA-B44:07	
HLA-A29:12		HLA-A68:23	HLA-B35:58	HLA-B39:24	HLA-B44:13	
HLA-A29:13		HLA-A68:24	HLA-B35:60	HLA-B39:26	HLA-B44:14	
		HLA-A68:25	HLA-B35:61	HLA-B39:27	HLA-B44:16	
		HLA-A68:26	HLA-B38:06	HLA-B39:28	HLA-B44:20	
		HLA-A74:01	HLA-B38:07	HLA-B39:29	HLA-B44:21	
		HLA-A74:02	HLA-B39:10	HLA-B39:30	HLA-B44:22	
		HLA-A74:03	HLA-B39:16	HLA-B39:32	HLA-B44:24	
		HLA-A74:04	HLA-B39:17	HLA-B39:33	HLA-B44:25	
		HLA-A74:05	HLA-B42:01	HLA-B39:34	HLA-B44:26	
		HLA-A74:06	HLA-B42:04	HLA-B40:12	HLA-B44:27	
		HLA-A74:07	HLA-B42:05	HLA-B44:40	HLA-B44:28	
		HLA-A74:08	HLA-B42:06	HLA-B48:01	HLA-B44:29	
		HLA-A74:09	HLA-B51:01	HLA-B48:02	HLA-B44:30	
		HLA-A74:11	HLA-B51:02	HLA-B48:03	HLA-B44:31	
		HLA-A02:52	HLA-B51:04	HLA-B48:04	HLA-B44:32	
		HLA-A30:01	HLA-B51:05	HLA-B48:05	HLA-B44:33	
		HLA-A30:08	HLA-B51:06	HLA-B48:07	HLA-B44:34	
		HLA-A30:11	HLA-B51:08	HLA-B48:08	HLA-B44:35	
		HLA-A30:13	HLA-B51:09	HLA-B48:09	HLA-B44:36	
		HLA-A30:15	HLA-B51:12	HLA-B48:10	HLA-B44:37	
		HLA-A68:06	HLA-B51:13	HLA-B48:11	HLA-B44:38	
		HLA-A68:07	HLA-B51:14	HLA-B48:12	HLA-B44:39	
			HLA-B51:16	HLA-B48:13	HLA-B44:41	
			HLA-B51:17	HLA-B55:18	HLA-B44:42	
			HLA-B51:18	HLA-B73:01	HLA-B45:01	
			HLA-B51:19		HLA-B45:02	
			HLA-B51:21		HLA-B45:03	
			HLA-B51:23		HLA-B45:04	
			HLA-B51:24		HLA-B45:05	
			HLA-B51:26		HLA-B45:07	
			HLA-B51:28		HLA-B47:04	
			HLA-B51:29		HLA-B47:05	
			HLA-B51:30		HLA-B49:04	
			HLA-B51:31		HLA-B50:01	
			HLA-B51:32		HLA-B50:02	
			HLA-B51:33		HLA-B50:04	
			HLA-B51:34			
			HLA-B51:35			
			HLA-B51:36			
			HLA-B51:37			
			HLA-B51:38			
			HLA-B53:01			
			HLA-B53:02			
			HLA-B53:04			
			HLA-B53:06			
			HLA-B53:08			
			HLA-B53:10			
			HLA-B54:01			
			HLA-B54:03			
			HLA-B54:04			
			HLA-B54:06			
			HLA-B54:07			
			HLA-B55:01			

HLA-B55:03
HLA-B55:04
HLA-B55:05
HLA-B55:07
HLA-B55:08
HLA-B55:09
HLA-B55:10
HLA-B55:11
HLA-B55:13
HLA-B55:14
HLA-B55:15
HLA-B55:17
HLA-B55:19
HLA-B56:01
HLA-B56:02
HLA-B56:03
HLA-B56:04
HLA-B56:05
HLA-B56:09
HLA-B56:10
HLA-B56:11
HLA-B56:12
HLA-B56:13
HLA-B56:15
HLA-B56:16
HLA-B67:01
HLA-B78:01
HLA-B78:02
HLA-B78:04
HLA-B81:01
